# Copper‐Catalysed Synthesis of (*E*)‐Allylic Organophosphorus Derivatives: A Low Toxic, Mild, Economical, and Ligand‐Free Method

**DOI:** 10.1002/cssc.202401450

**Published:** 2024-10-25

**Authors:** Lucas Pagès, Gracjan Kurpik, Rosa Mollfulleda, Racha Abed Ali Abdine, Anna Walczak, Florian Monnier, Marcel Swart, Artur R. Stefankiewicz, Marc Taillefer

**Affiliations:** ^1^ ICGM Université Montpellier, CNRS, ENSCM Montpellier France; ^2^ Center for Advanced Technologies Adam Mickiewicz University in Poznań Uniwersytetu Poznańskiego 10 61-614 Poznań Poland; ^3^ Faculty of Chemistry Adam Mickiewicz University in Poznań Uniwersytetu Poznańskiego 8 61-614 Poznań Poland; ^4^ IQCC and Department of Chemistry Universitat de Girona c/M.A. Capmany 69 17003 Girona Spain; ^5^ ICREA Passeig Lluís Companys 23 08010 Barcelona Spain

**Keywords:** Copper, Catalysis, Hydrophosphorylation, Allenes, DFT calculations

## Abstract

Organophosphorus compounds are fundamental for the chemical industry due to their broad applications across multiple sectors, including pharmaceuticals, agrochemicals, and materials science. Despite their high importance, the sustainable and cost‐effective synthesis of organophoshoryl derivatives remains very challenging. Here, we report the first successful regio‐ and stereoselective hydrophosphorylation of terminal allenamides using an affordable copper catalyst system. This reaction offers an efficient protocol for the synthesis of (*E*)‐allylic organophosphorus derivatives from various types of *P*‐nucleophiles, such as *H*‐phosphonates, *H*‐phosphinates, and secondary phosphine oxides. Key advantages of this ligand‐free and atom‐economic strategy include low toxicity of the Cu‐based catalyst, cost effectiveness, mild reaction conditions, and experimental simplicity, making it competitive with methods that use toxic and expensive Pd‐based catalysts. In an effort to comprehend this process, we conducted extensive DFT calculations on this system to uncover the mechanistic insights of this process.

## Graphical abstract

We pre*s*ent the first example of copper‐catalysed intermolecular hydrophosphorylation of N‐allenyl derivatives. Low toxicity, cost‐effectiveness, mildness and experimental simplicity are the hallmarks of this ligand‐free and atom‐economic strategy, which lead to various (E)‐allylic organophosphorus compounds.

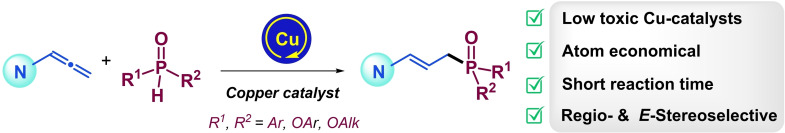

## Introduction

Organophosphorus compounds have attracted significant attention of the scientific community due to their high application potential in pharmacy, biochemistry, materials, and medicinal chemistry.^[1]^ Indeed, a phosphoryl P(O) group is found in a wide variety of biologically active molecules, pharmaceuticals, herbicides, and a number of phosphines are commonly used as ligands.^[5]^ However, the ongoing development of more efficient, selective, and sustainable synthetic strategies for C−P bond formation remains a major challenge.^[6]^ In this context, one of the simplest synthetic routes involves the direct addition of a P(O)−H nucleophile to C−C multiple bonds, *i. e*., the hydrophosphorylation reaction, which is usually performed in the presence of transition metal based catalysts, mainly Pd catalysts.^[14]^ This approach allows a total economy of atoms and contrasts with more traditional methods such as the Michaelis‐Arbuzov reaction or those involving phosphoryl radicals.^[20]^

**Scheme 1:**
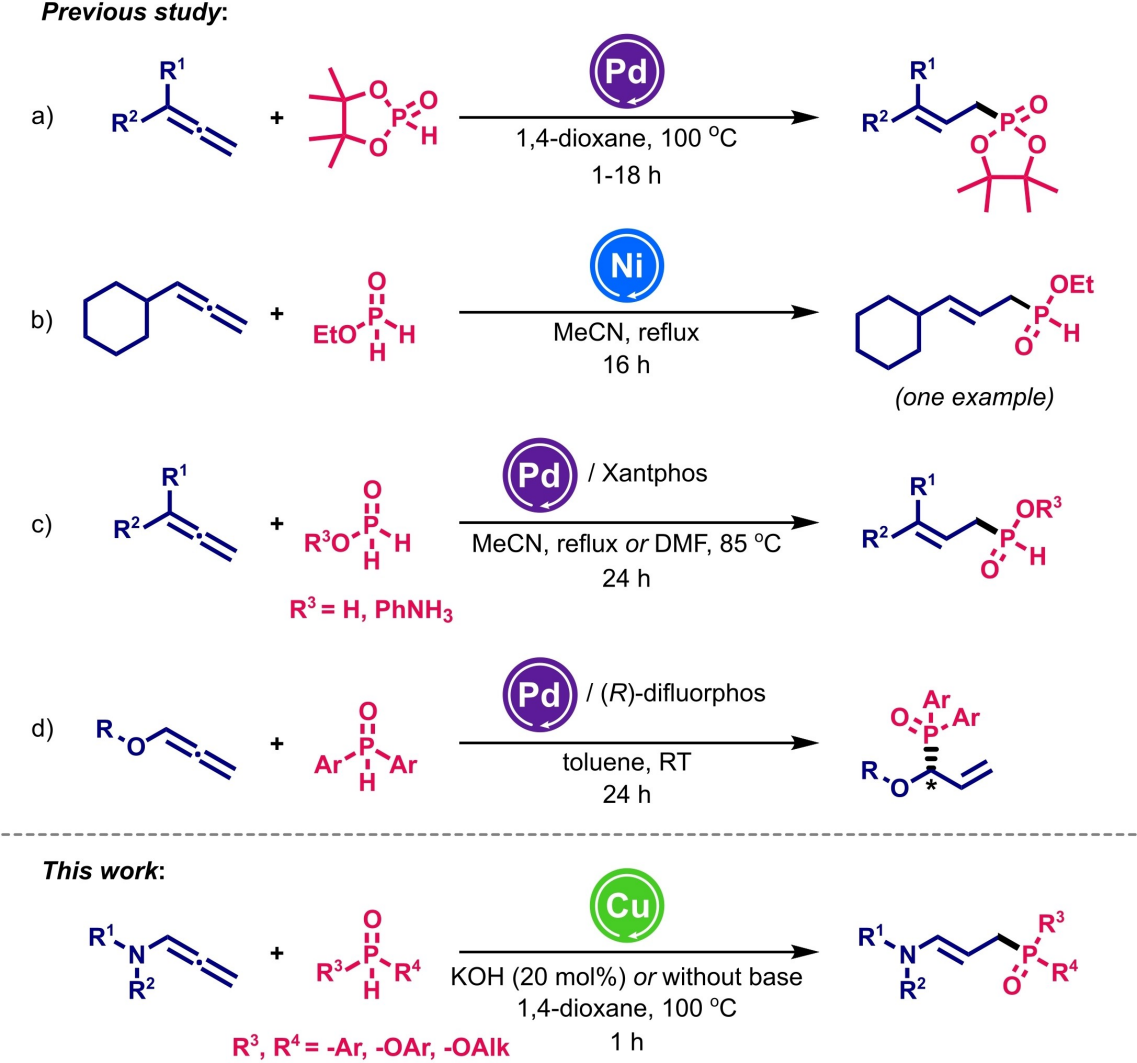
Metal‐catalysed hydrophosphorylation of allenes – known methods and a novel pathway described herein.

Although numerous reports on the catalytic addition of phosphorus nucleophiles to alkenes and alkynes are available in the literature,^[24]^ the methodologies for allene hydrophosphorylation remain highly limited. To the best of our knowledge, there are only a few synthetic protocols known, each tailored for very narrow and specific classes of reagents (Scheme 1). For instance, Tanaka and Han elaborated an efficient system for the Pd‐catalysed synthesis of allylphosphonates from terminal allenes with good regio‐ and stereoselectivity (Scheme 1a).^[30]^ Despite the high reactivity observed with the five‐membered cyclic *H*‐phosphonates, other *P*‐nucleophiles such as alkyl hydrogen phosphonates turned out to be totally unreactive, which significantly confines the broad utility of the method. In 2005, Montchamp *et al*. reported the NiCl_2_‐catalysed hydrophosphorylation of alkynes, which was also applied in an isolated example to the cyclohexylallene and ethyl phosphinate (Scheme 1b). Although a low isolated yield (27 %) was obtained, the method represents the sole case of functionalisation by a catalytic system other than those based on Pd.^[31]^ Furthermore, the same authors developed allene hydrophosphorylation with hypophosphorous acid and its anilinium salt, catalysed by the Pd_2_dba_3_/Xantphos system, obtaining a range of allylic *H*‐phosphinates (Scheme 1c).^[18]^ More recently, the Pd‐catalysed addition of phosphine oxides to alkyl and aryl‐oxyallenes was reported, producing chiral allylic phosphine oxides with high yield and enantioselectivity using (*R*)‐difluorphos as a chiral ligand (Scheme 1d).^[32]^

Despite the considerable advances, these methodologies have certain limitations, such as efficiency, substrate specificity and, above all, the use of very expensive and toxic Pd‐based catalysts. Indeed, the maximum metal concentration limit for the oral absorption of drugs & excipients products (*Permitted Daily Exposure ‐ PDE*) is only 100 μg for Pd (200 μg for Ni).^[33]^ Therefore, developing a new route for synthesising organophosphorus compounds via allene hydrophosphorylation using low toxic but also cost‐effective and efficient catalytic systems, is urgently needed. Additionally, comprehensive mechanistic studies on the catalytic systems based on metal other than Pd have yet to be conducted, leaving many questions unanswered.

In this report, we present the first example of intermolecular hydrophosphorylation of *N*‐allenyl derivatives catalysed by a straightforward low‐toxicity and low‐cost Cu‐based catalyst. This ligand‐free and atom‐economic strategy offers easy access to a wide range of structurally distinct (*E*)‐allylic organophosphorus compounds in moderate to excellent yields. In favour of this method are not only its high selectivity, efficiency and broad tolerance for organophosphorus nucleophiles, but also elimination of highly toxic and costly Pd‐catalysts. Thus, the use of Cu‐based catalysts, which distinguish it from other protocols of the literature, is a crucial and decisive advantage for the synthesis of drugs. Indeed, the maximum metal concentration limit for the oral absorption of drugs & excipients products (PDE) is 3400 μg for Cu and no more than 100 μg for Pd.^[33]^ The results demonstrated the synthetic versatility of the proposed approach and address the mechanistic questions associated with the reaction.

## Results and Discussion

A first series of experiments was carried out using *N*‐allenyl‐2‐pyrrolidinone **1** **a** and diethylphosphite **2** **a** as model substrates. Initially, we tested the reaction in 1,4‐dioxane at 100 °C in the presence of [Cu(MeCN)_4_]PF_6_ (10 mol %) as a catalyst precursor. After 18 h, we obtained the hydrophosphorylation product **3** **a** **a** resulting from the addition of the phosphorus moiety on the terminal carbon of the allene **1** **a** in a 27 % yield (Table 1, entry 1). The addition of a catalytic amount of base (20 mol %) proved crucial for improving the system's performance (Table 1, entries 2–7). Specifically, KOH and *t‐*BuOK led to the formation of the (*E*)‐allylic phosphonate **3** **a** **a** in 95 % and 98 % yields, respectively. The use of other bases, such as Cs_2_CO_3_, (*i*Pr)_2_NH, K_2_CO_3_, or K_3_PO_4_ was less effective. Lower yields were also observed when replacing 1,4‐dioxane with other solvents, such as THF (60 %) or 2‐MeTHF (57 %). Due to the cost effectiveness and greater availability of KOH compared to *t‐*BuOK, we used the former in further studies. We found that reducing the catalyst loading (from 10 to 5 mol %) or the amount of base (from 20 to 10 mol %) still afforded high, but slightly lower conversions (Table 1, entries 8 and 9). A very low yield of 9 % of **3** **a** **a** was obtained in a blank experiment performed in the absence of [Cu(MeCN)_4_]PF_6_ (Table 1, entry 10). Using CuI as an alternative Cu(I) source reduced the yield by around 40 % (Table 1, entry 11), and a moderate yield (41 %) was obtained by decreasing the temperature from 100 to 50 °C (Table 1, entry 12). Finally, monitoring the reaction progress over time (Table 1, entry 13), showed almost quantitative conversion after just 1 h. These last conditions, which allowed the formation of the (*E*)‐allylic phosphonate **3** **a** **a** in 98 % NMR yield, were chosen to study the scope of the reaction.

**Table 1:** Cu‐catalysed hydrophosphorylation reaction between allenamide **1** **a** and diethylphosphite **2** **a** : optimisation of the reaction conditions.^[a]^

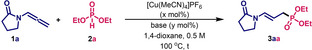				
	x (mol %)	base (y mol %)	time (h)	yield (%)^[b]^
1	10	–	18	27
2	10	Cs_2_CO_3_ (20)	18	32
3	10	(*i*Pr)_2_NH (20)	18	56
4	10	K_2_CO_3_ (20)	18	57
5	10	K_3_PO_4_ (20)	18	75
6	10	*t‐*BuOK (20)	18	98
7	10	KOH (20)	18	95
8	5	KOH (20)	18	88
9	10	KOH (10)	18	86
10	–	KOH (20)	18	9
11	10 (CuI)	KOH (20)	18	55
12	10	KOH (20)	18	41^[c]^
13	10	KOH (20)	1	98

[a] Reactions conditions: *N*‐allenyl‐2‐pyrrolidinone **1** **a** (0.2 mmol, 1.0 equiv.), diethyl phosphite **2** **a** (0.24 mmol, 1.2 equiv.), [Cu]‐catalyst (0.01–0.02 mmol), and base (0.04–0.2 mmol) were placed in a screw tube under nitrogen atmosphere in 1,4‐dioxane (0.4 mL) at 100 °C. [b] NMR yields using 1,3,5‐trimethoxybenzene as an internal standard. [c] At 50 °C.

Insight into the reaction mechanism was obtained by a DFT study at the S12g/TZ2P (COSMO, ZORA) level on this model system. As first step we recomputed the intermediates and transition states of a recent study on a related reaction (carried out with different DFT methods). This was done for the hydroamination of allenes,^[34]^ to validate the use of S12g for these reactions. As similar structures and energy barriers were obtained, confirming the reliability of S12g for these catalytic cycles, we assumed a similar mechanism might be in play for our reaction.

We therefore started with the Cu(I) ion bound to four MeCN molecules in 1,4‐dioxane and assumed that upon adding diethylphosphite **2** **a** , these MeCN molecules are replaced by two molecules of **2** **a** , which is corroborated by our DFT data. Note that due to the importance of base for the reaction to occur, we explored all possible scenarios for the protonation states of the phosphites, with either two protonated [P(O)(OEt)_2_(H)]^0^ (POH) bound to Cu(I) ion, two deprotonated [P(O)(OEt)_2_]^1−^ (PO), or one protonated and one deprotonated.

Our initial calculations indicate that the binding of two POHs to Cu(I) is endergonic, while the other two combinations are exergonic. This Cu(I) species [Cu(POH)_x_(PO)_y_]^+/0/−^ (x,y={2,0}, {1,1}, {0,2}) then binds to allenamide **1** **a** , after which a third phosphite (POH or PO) performs a nucleophilic addition to the double bond. We therefore have six possible scenarios for the reaction mechanism, ranging from all three protonated to all deprotonated. Furthermore, the nucleophilic attack by the third phosphite could occur in *anti* or *syn* fashion with respect to the Cu(I) species, and in *Z* or *E* orientation relative to the pyrrolidone ring. Overall, we explored 36 routes for the transition state of the nucleophilic attack. We observed that the most favourable barriers and most exergonic product formation were obtained with two of the phosphites deprotonated, one of which acts as a nucleophile.

**Figure 1:**
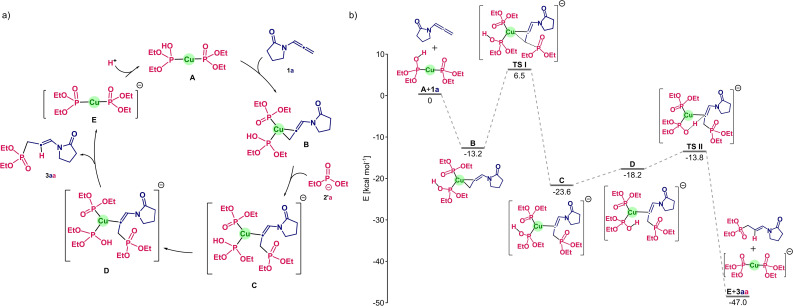
a) Proposed mechanism for the Cu‐catalysed hydrophosphorylation, illustrated with allenamide **1** **a** and diethylphosphite **2** **a** . b) Gibbs free energy profile (S12g/TZ2P, COSMO, ZORA; see SI for details) at 100 °C for the Cu‐catalysed hydrophosphorylation of allenamide **1** **a** and diethylphosphite **2** **a** , where the active species **A** consists of one protonated and one deprotonated phosphite and the nucleophile is deprotonated (see text).

Hence, the binding of phosphites on the Cu(I) ion occurs with one deprotonated and one protonated phosphite, leading overall to a charge‐neutral Cu(I) complex **A** (Figure 1a). This outcome corroborates the experimental results, which indicated that two equivalents of base were needed (*vide supra*). In the most favoured conformation of the Cu(I) complex, the metal centre is bound to both carbon atoms of the terminal (formally) double bond of the allene, with Cu−C distances of 1.97 Å (**B**, Figure 1a). The nucleophilic phosphite then attacks the carbon double bond from below, leading to a breaking of one of these Cu−C bonds, and the formation of a C−P bond (**C**, Figure 1a). This step requires crossing a barrier of *ca*. 20 kcal mol^−1^ (TS I), which is the rate‐determining step of the mechanism. Afterwards, at the protonated phosphite nucleophile an internal rotation takes place that positions the proton near the Cu−C bond, *i. e*., in an optimal position for proton transfer towards the distal carbon (**D**, Figure 1a). The transition state to go from **D** to **E** has a reaction barrier of *ca*. 5 kcal mol^−1^, leading to the product. Depending on the orientation of the nucleophile and the phosphite ligands on the Cu(I) ion, different pathways have been located, but they involve crossing transition state structures that are higher in energy by 3.5 to 13.5 kcal mol^−1^. The last step is the protonation of the phosphite to close the catalytic cycle and regenerate the starting point; the proton could come from a protonated phosphite to generate the active species, or from the medium, with base present, allowing two phosphites can be deprotonated (see Figure 1b).

We then undertook studies of the scope of the method. Three classes of allenamides, *i. e*., amides, carbamates, and sulfonamides, were tested with a number of readily available *H*‐phosphonates containing alkyl (**2** **a** – **c** ), aryl (**2** **d** ), and benzyl (**2** **e** ) moieties (Table 2). In the case of *N*‐allenyl‐2‐pyrrolidinone **1** **a** , the products **3** **a** **a** – **a** **e** were obtained in moderate to excellent isolated yields, ranging from 55 % to 90 %, not significantly dependent on the nature of the phosphite used. In the following set of experiments, *N*‐allenyl‐2‐oxazolidinone **1** **b** was successfully engaged in the Cu‐catalysed hydrophosphorylation, affording the corresponding products **3** **b** **a** – **b** **e** in good to high yields (50–80 %). Under standard reaction conditions, *N*‐allenyl‐sulfonamide **1** **c** also gave the corresponding compounds (**3** **c** **a** – **c** **e** ) in isolated yields ranging from 43 to 80 %. It is noteworthy that all the isolated products present exclusively the (*E*)‐configuration of the double bond as evidenced *via* ^1^H NMR analysis (see Supporting Information). The synthetic usefulness of the method was amply highlighted by a gram scale synthesis involving model substrates **1** **a** and **2** **a** . The reaction, carried out on a 2 mmol scale, gave the corresponding hydrophosphorylation product **3** **a** **a** in similar yield (85 %) to that obtained on a 0.2 mmol scale.

**Table 2:**
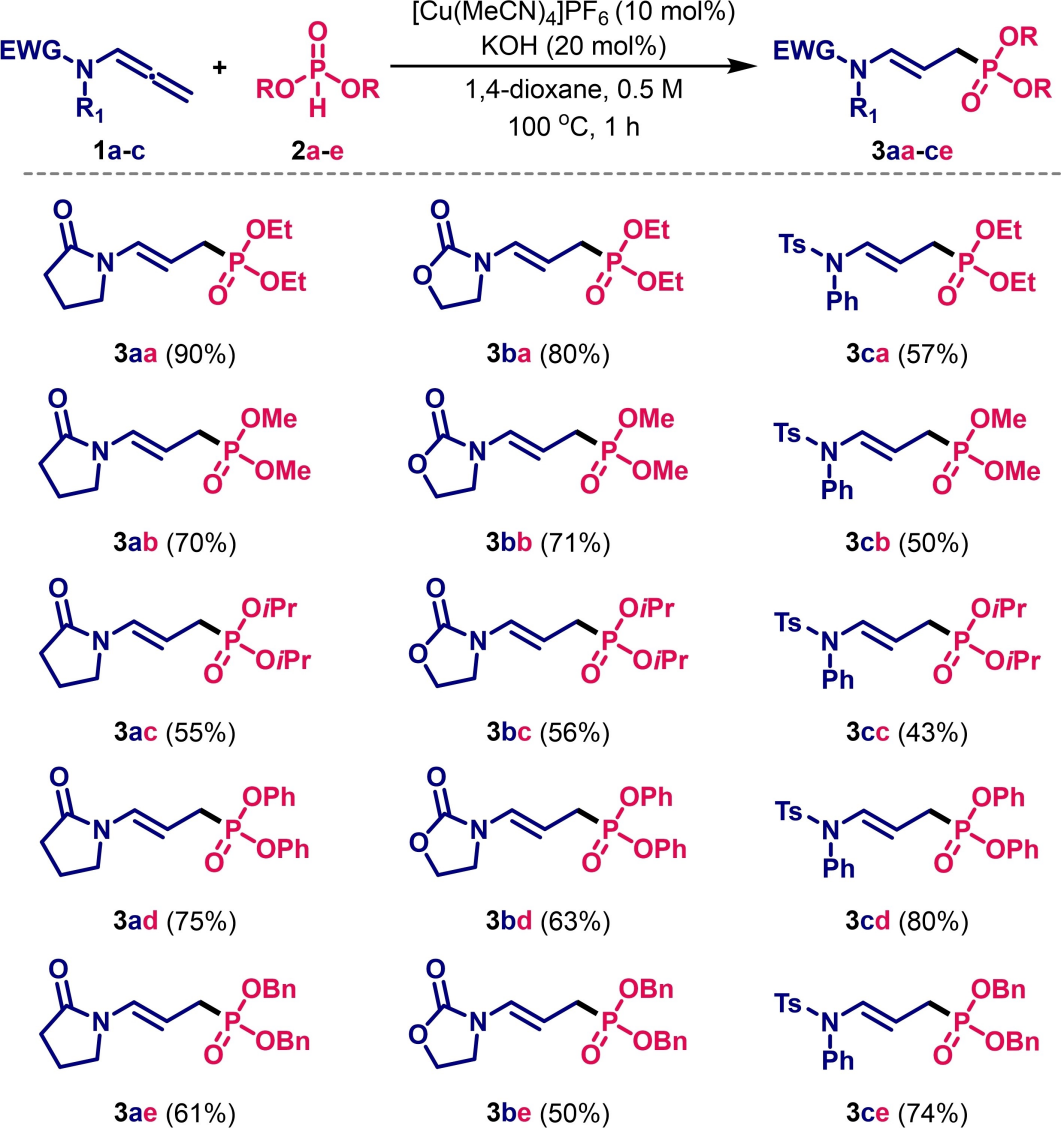
Cu‐catalysed hydrofunctionalisation of allenes **1** **a–c** with structurally distinct phosphites **2** **a–e** .^[a,b]^

[a] Reactions conditions: **1** **a** – **c** (0.2 mmol), **2** **a** – **e** (0.24 mmol), [Cu(MeCN)_4_]PF_6_ (10 mol %), and KOH (20 mol %) were placed in a screw tube under nitrogen atmosphere in 1,4‐dioxane (0.5 M) at 100 °C for 1 h. [b] NMR yields using 1,3,5‐trimethoxybenzene as an internal standard.

We then investigated this system with other *P*‐nucleophiles, *i. e*., secondary phosphine oxides and *H*–phosphinates. By carrying out blank experiments on the reaction of *N*‐allenyl‐2‐pyrrolidinone **1** **a** with diphenylphosphine oxide **4** **a** , we observed that the presence of the base was not necessary. Indeed, under the previous conditions but in the absence of KOH, we obtained the expected product **5** **a** **a** in 70 % isolated yield (Table 3). Moreover, diphenyl phosphine oxide **4** **a** reacted with *N*‐allenyl‐2‐oxazolidinone **1** **b** and *N*‐allenyl sulfonamide **1** **c** to give the corresponding hydrofunctionalisation products in 68 % and 44 % reaction yields, respectively. Another class of organophosphorus derivatives, namely *H*‐phosphinates with ethyl phenylphosphinate **6a** as a representative, was engaged under similar conditions with allenamide **1a**, affording the corresponding compound **7a** (Table 3). Secondary phosphine oxides, or heteroatom‐substituted secondary phosphine oxides are weak acids that can tautomerize in solution to trivalent P(III) phosphinous acids.^[36]^ For phosphinylidenes, the prototropic tautomerism is generally strongly shifted to the P(V) form. When our catalytic system operates in the absence of a base, the ability to reach the P(III) tautomer, which is then probably involved as a nucleophile, is essential. Since the presence of a strong donor group such as alkoxy (compared to phenyl) is known to destabilise the P(III) form, this could explain why, in the absence of a base, the reaction with phosphites is less efficient than with diphenylphosphine oxides (Tables 2&3).^[38]^ However, we must be cautious with this type of explanation, as the tautomeric equilibrium depends on many factors, such as thermodynamic stabilisation and the kinetic rate of P(III) formation, as well as the reaction with the Cu‐based catalyst.

**Table 3:**
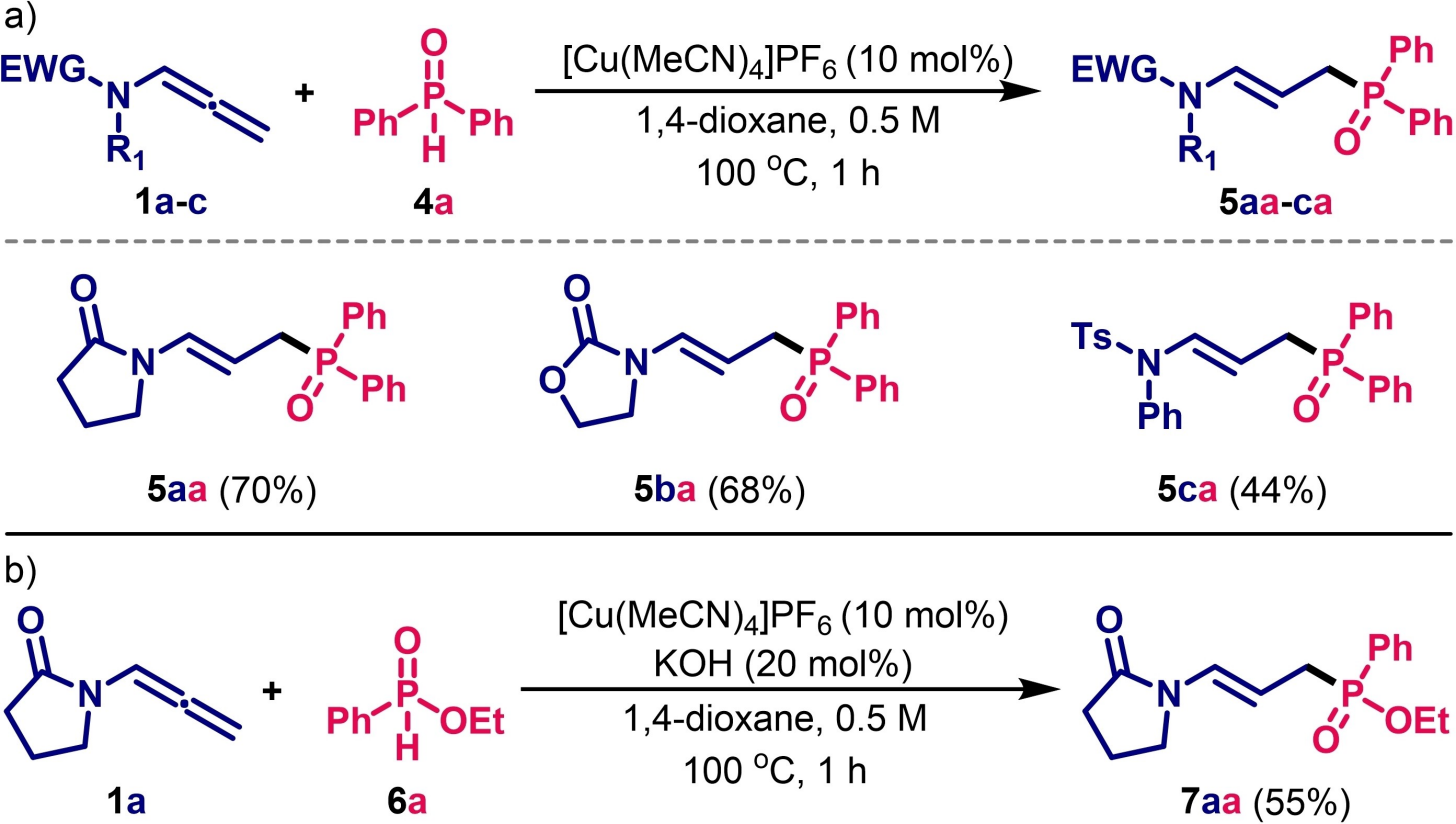
Cu‐catalysed hydrofunctionalisation of allenamides with: a) diphenyl phosphine oxide **4** **a** ; b) ethyl phenylphosphinate **6** **a** .^[a,b]^

[a] Reactions conditions: **1** **a** – **c** (0.2 mmol), **4** **a** or **6** **a** (0.24 mmol), [Cu(MeCN)_4_]PF_6_ (10 mol %), and KOH (only in *b*, 20 mol %) were placed in a screw tube under nitrogen atmosphere in 1,4‐dioxane (0.5 M) at 100 °C for 1 h. [b] NMR yields using 1,3,5‐trimethoxybenzene as an internal standard.

## Conclusions

In conclusion, we have reported the first example of a Cu‐catalysed C−P bond formation *via* total regio‐ and stereoselective hydrophosphorylation of terminal allenamides, thus providing an efficient access to phosphorylated (*E*)‐enamides in a completely atom‐ and step‐economical fashion. This synthetic strategy incorporates a diverse array of *N*‐allenyl derivatives and *P*‐nucleophiles, such as *H*‐phosphonates, *H*‐phosphinates, and secondary phosphine oxides, yielding a broad scope of products in moderate to high yields. Mechanistic insights provided by DFT calculation studies allowed us to propose a mechanism with a charge‐neutral Cu(I) complex as the catalytically active species, with the metal ion bound to one deprotonated and one protonated phosphite. The low toxicity of the Cu catalyst, cost effectiveness, mild conditions, and experimental simplicity are the hallmarks of this ligand‐free strategy, making it competitive with those using toxic and expensive Pd catalysts, often limited by substrate specificity. Given all these considerations, the proposed strategy may achieve practical applications in the efficient and sustainable synthesis of fine chemicals, pharmaceuticals, and materials.

## Conflict of Interests

The authors declare no conflict of interest.

1

## Supporting information

As a service to our authors and readers, this journal provides supporting information supplied by the authors. Such materials are peer reviewed and may be re‐organized for online delivery, but are not copy‐edited or typeset. Technical support issues arising from supporting information (other than missing files) should be addressed to the authors.

Supporting Information

## Data Availability

The data that support the findings of this study are available in the supplementary material of this article.
