# Supplementary material for: Copper‐Catalysed Synthesis of (E)‐Allylic Organophosphorus Derivatives: A Low Toxic, Mild, Economical, and Ligand‐Free Method
Source: ChemSusChem. 2024 Oct 25;18(2):e202401450. doi: 10.1002/cssc.202401450 (PMC11739839; doi:10.1002/cssc.202401450)
Supplement: Supplementary file 1 — Supporting Information [file CSSC-18-e202401450-s001.pdf]

ChemSusChem

Supporting Information

Copper-Catalysed Synthesis of (*E*)-Allylic Organophosphorus Derivatives: A Low Toxic, Mild, Economical, and Ligand-Free Method

Lucas Pagès, Gracjan Kurpik, Rosa Mollfulleda, Racha Abed Ali Abdine, Anna Walczak, Florian Monnier, Marcel Swart,* Artur R. Stefankiewicz,* and Marc Taillefer*

Supporting Information for

Copper-Catalysed Synthesis of (*E*)-Allylic Organophosphorus Derivatives: A Low Toxic, Mild, Economical, and Ligand-Free Method

Lucas Pagès,[‡a] Gracjan Kurpik,[‡b,c] Rosa Mollfulleda,[d] Racha Abed Ali Abdine,[a] Anna Walczak,[b,c] Florian Monnier,[a] Marcel Swart,[d,e] Artur R. Stefankiewicz,*[b,c] and Marc Taillefer*[a]*

Table of contents

1. General considerations 2
2. Reaction development for the hydrophosphorylation reaction 2
3. General procedure for the hydrophosphorylation reaction of allenes 2
4. General procedure for the hydrophosphinylation reaction of allenes 3
5. General procedure for large scale synthesis 3
6. Characterization data for the hydrophosporylation reaction products 3
7. Kinetic studies 11
8. Computational details 12
9. NMR spectra 12
10. References 41

1. General considerations

All reactions were performed in oven-dried Schlenk flasks closed with a Rodaviss screw cap and PTFE liner (approx. 25 mL). All reagents were purchased from commercial sources (Sigma-Aldrich, Alfa Aesar, or Acros Organic) and were used without further purification and weighed to air. All allenes were synthesized according to previously described methods, and analyses were in accordance with the literature.

^{1}H, ^{13}C and ^{31}P NMR spectra were recorded with a Bruker AC-400 MHz spectrometer in $CDCl_3$, and the residual solvent protons (7.26 for ^{1}H) or carbons (77.16 for ^{13}C) were used as internal references. All ^{1}H NMR spectra were obtained with complete phosphorus decoupling, ^{13}C NMR and ^{31}P NMR spectra – with complete proton decoupling. Chemical shifts (δ) are reported in parts per million (ppm), and the coupling constants (J) are reported in Hertz (Hz). The following abbreviations are used: *s*, singlet; *d*, doublet; *dd*, doublet of doublets; *dt*, doublet of triplets; *t*, triplet; *qd*, quartet of doublets; *p*, pentet; *hept*, heptuplet; and *m*, multiplet.

A TOF-type mass analyzer was used for the HRMS measurements. Electrospray ionization (ESI) high-resolution mass spectra were recorded on a Waters SYNAPT G2-S (SN: UEB205) high-definition mass spectrometer in the positive ion mode from 50 to 2200 Da. Products were dissolved in MeOH, depending on the case, and were introduced directly into the spectrometer. The capillary voltage was 3000 V, and the cone voltage was 30 V. The source and desolvation temperatures were 100 and 150 °C respectively. The data were reprocessed by the Masslynx 4.1 software.

2. Reaction development for the hydrophosphorylation reaction

Table S1. Copper-catalyzed hydrophosphorylation reaction of allene **1a** with diethyl phosphite **2a**: Selected data for reaction development.[a]

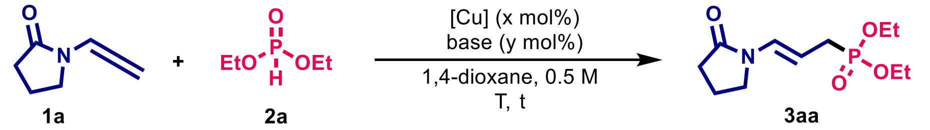

entry	[Cu] (x mol%)	base (y mol%)	T (°C)	time (h)	NMR yield (%)[b]
1.	$[Cu(MeCN)_4]PF_6$ (10)	-	100	18	27
2.	$[Cu(MeCN)_4]PF_6$ (10)	K_2CO_3 (20)	100	18	57
3.	$[Cu(MeCN)_4]PF_6$ (10)	K_3PO_4 (20)	100	18	75
4.	$[Cu(MeCN)_4]PF_6$ (10)	Cs_2CO_3 (20)	100	18	32
5.	$[Cu(MeCN)_4]PF_6$ (10)	DIPA (20)	100	18	56
6.	$[Cu(MeCN)_4]PF_6$ (10)	tBuOK (20)	100	18	98
7.	$[Cu(MeCN)_4]PF_6$ (10)	KOH (20)	100	18	95
8.	$[Cu(MeCN)_4]PF_6$ (10)	KOH (100)	100	18	42
9.	-	KOH (20)	100	18	9
10.	$[Cu(MeCN)_4]PF_6$ (10)	KOH (20)	50	18	41
11.	CuI (10)	KOH (20)	100	18	55
12.	$[Cu(MeCN)_4]PF_6$ (5)	KOH (20)	100	18	88
13.	$[Cu(MeCN)_4]PF_6$ (10)	KOH (10)	100	18	86
14.	$[Cu(MeCN)_4]PF_6$ (10)	KOH (20)	100	6	97
**15.**	**$[Cu(MeCN)_4]PF_6$ (10)**	**KOH (20)**	**100**	**1**	**98**

[a] Reactions conditions: *N*-allenyl-2-pyrrolidinone **1a** (0.2 mmol, 1.0 equiv.), diethyl phosphite (0.24 mmol, 1.2 equiv.), [Cu]-catalyst (0.01 to 0.02 mmol) and base (0.04 to 0.2 mmol) were placed in a screw tube under nitrogen atmosphere in 1,4-dioxane (0.4 mL). [b] NMR yields using 1,3,5-trimethoxybenzene as an internal standard.

3. General procedure for the hydrophosphorylation reaction of allenes

An oven-dried Schlenk flask of appropriate size was charged with allene (if solid, 0.2 mmol, 1.0 equiv.), $[Cu(MeCN)_4]PF_6$ (0.02 mmol, 0.1 equiv.), KOH (0.04 mmol, 0.2 equiv.) and closed with a rubber septum. After evacuation and backfilling with nitrogen gas three times, dry 1,4-dioxane (0.5 M, 0.4 mL), phosphite (0.24 mmol, 1.2 equiv.) and allene (if liquid, 0.2 mmol, 1.0 equiv) were sequentially added under a stream of nitrogen. The

vessel was closed and stirred at 100 °C for 1 h. After allowing the reaction to cool to room temperature, 1,3,5-trimethoxybenzene (0.33 equiv.) dissolved in dichloromethane was added as an internal standard to estimate the NMR yield. After an aqueous workup, the organic phase was separated. The remaining aqueous layer was further extracted with dichloromethane and the combined organic phases were then dried over anhydrous $MgSO_4$, filtered and concentrated under vacuum. The residue was purified by Et_3N-treated silica gel column chromatography.

4. General procedure for the hydrophosphinylation reaction of allenes

An oven-dried Schlenk flask of appropriate size was charged with allene (if solid, 0.2 mmol, 1.0 equiv.), $[Cu(MeCN)_4]PF_6$ (0.02 mmol, 0.1 equiv.), and closed with a rubber septum. After evacuation and backfilling with nitrogen gas three times, dry 1,4-dioxane (0.5 M, 0.4 mL), phosphine oxide (0.24 mmol, 1.2 equiv.) and allene (if liquid, 0.2 mmol, 1.0 equiv) were sequentially added under a stream of nitrogen. The vessel was closed and stirred at 100 °C for 1 h. After allowing the reaction to cool to room temperature, 1,3,5- trimethoxybenzene (0.33 equiv.) dissolved in dichloromethane was added as an internal standard to estimate the NMR yield. After an aqueous workup, the organic phase was separated. The remaining aqueous layer was further extracted with dichloromethane and the combined organic phases were then dried over anhydrous $MgSO_4$, filtered and concentrated under vacuum. The residue was purified by Et_3N-treated silica gel column chromatography.

5. General procedure for large scale synthesis

An oven-dried Schlenk flask of appropriate size was charged with $[Cu(MeCN)_4]PF_6$ (0.2 mmol, 0.1 equiv.), KOH (0.4 mmol, 0.2 equiv.) and closed with a rubber septum. After evacuation and backfilling with nitrogen gas three times, dry 1,4-dioxane (0.5 M, 4 mL), *N*-allenyl-2-pyrrolidinone **1a** (2.0 mmol, 1.0 equiv) and diethyl phosphite **2a** (2.4 mmol, 1.2 equiv.) were sequentially added under a stream of nitrogen. The vessel was closed and stirred at 100 °C for 1 h. After an aqueous workup, the organic phase was separated. The remaining aqueous layer was further extracted with dichloromethane and the combined organic phases were then dried over anhydrous $MgSO_4$, filtered and concentrated under vacuum. The residue was purified by Et_3N-treated silica gel column chromatography.

6. Characterization data for the hydrophosporylation reaction products

6.1. *Diethyl (E)-(3-(2-oxopyrrolidin-1-yl)allyl)phosphonate 3aa*

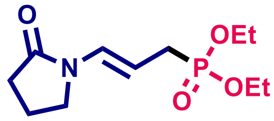

3aa

The reaction of *N*-allenyl-2-pyrrolidinone **1a** (0.2 mmol, 1 equiv.) with diethyl phosphite **2a** (0.24 mmol, 1.2 equiv.) afforded the corresponding hydrophosphorylation product **3aa** as a yellow oil according to the general procedure (flash chromatography: DCM/iPrOH, 95:5).

^{1}H {^{31}P} NMR (400 MHz, $CDCl_3$) δ = 6.98 (d, *J* = 14.4 Hz, 1H), 4.89 (dt, *J* = 14.6, 7.6 Hz, 1H), 4.09 (qd, *J* = 7.1, 1.3 Hz, 4H), 3.51 (t, *J* = 7.4, 1.3 Hz, 2H), 2.58 (dd, *J* = 7.6, 1.3 Hz, 2H), 2.47 (t, *J* = 8.2 Hz, 2H), 2.14 – 2.06 (m, 2H), 1.30 (t, *J* = 7.1 Hz, 6H).

^{13}C NMR {^{1}H} (101 MHz, $CDCl_3$) δ = 173.15, 127.36 (d, *J* = 15.7 Hz), 100.47 (d, *J* = 11.1 Hz), 62.12 (d, *J* = 6.8 Hz), 45.23, 31.20, 28.05 (d, *J* = 142.4 Hz), 17.50, 16.55 (d, *J* = 5.9 Hz).

^{31}P {^{1}H} NMR (162 MHz, $CDCl_3$) δ = 27.31.

HRMS (ESI) *m/z*: $[M+H]^+$ calcd. for $C_{11}H_{21}NO_4P$ 262.1203, found 262.1208.

6.2. *Dimethyl (E)-(3-(2-oxopyrrolidin-1-yl)allyl)phosphonate 3ab*

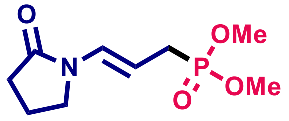

3ab

The reaction of *N*-allenyl-2-pyrrolidinone **1a** (0.2 mmol, 1 equiv.) with dimethyl phosphite **2b** (0.24 mmol, 1.2 equiv.) afforded the corresponding hydrophosphorylation product **3ab** as a light brown solid according to the general procedure (flash chromatography: DCM/iPrOH, 95:5).

^{1}H {^{31}P} NMR (400 MHz, $CDCl_3$) δ = 6.98 (d, *J* = 14.4 Hz, 1H), 4.87 (dt, *J* = 14.5, 7.6 Hz, 1H), 3.73 (s, 6H), 3.51 (t, *J* = 7.2 Hz, 2H), 2.60 (dd, *J* = 7.6, 1.3 Hz, 2H), 2.47 (t, *J* = 8.4 Hz, 2H), 2.14 – 2.05 (m, 2H).

^{13}C NMR {^{1}H} (101 MHz, $CDCl_3$) δ = 173.19, 127.56 (d, *J* = 15.6 Hz), 100.02 (d, *J* = 11.2 Hz), 52.89 (d, *J* = 6.8 Hz), 45.23, 31.21, 27.11 (d, *J* = 142.7 Hz), 17.53.

^{31}P {^{1}H} NMR (162 MHz, $CDCl_3$) δ = 29.77.

HRMS (ESI) *m/z*: $[M+H]^+$ calcd. for $C_9H_{17}NO_4P$ 234.0890, found 234.0894.

6.3. *Diisopropyl (E)-(3-(2-oxopyrrolidin-1-yl)allyl)phosphonate 3ac*

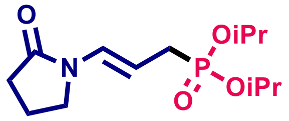

3ac

The reaction of *N*-allenyl-2-pyrrolidinone **1a** (0.2 mmol, 1 equiv.) with diisopropyl phosphite **2c** (0.24 mmol, 1.2 equiv.) afforded the corresponding hydrophosphorylation product **3ac** as a yellow oil according to the general procedure (flash chromatography: AcOEt/iPrOH, 90:10).

^{1}H {^{31}P} NMR (400 MHz, $CDCl_3$) δ = 6.92 (d, *J* = 14.4 Hz, 1H), 4.85 (dt, *J* = 14.4, 7.6 Hz, 1H), 4.63 (hept, *J* = 6.2 Hz, 2H), 3.47 (t, *J* = 7.2 Hz, 2H), 2.50 (dd, *J* = 7.6, 1.3 Hz, 2H), 2.43 (t, *J* = 8.6 Hz, 2H), 2.11 – 2.02 (m, 2H), 1.25 (t, *J* = 6.4 Hz, 12H).

^{13}C NMR {^{1}H} (101 MHz, $CDCl_3$) δ = 172.96, 127.12 (d, *J* = 15.7 Hz), 100.96 (d, *J* = 11.1 Hz), 70.42 (d, *J* = 6.9 Hz), 45.16, 31.15, 29.24 (d, *J* = 143.6 Hz), 24.08 (d, *J* = 4.3 Hz), 17.46.

^{31}P {^{1}H} NMR (162 MHz, $CDCl_3$) δ = 25.30.

HRMS (ESI) *m/z*: $[M+H]^+$ calcd. for $C_{13}H_{25}NO_4P$ 290.1516, found 290.1523.

6.4. *Diphenyl (E)-(3-(2-oxopyrrolidin-1-yl)allyl)phosphonate 3ad*

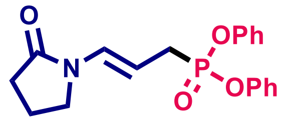

3ad

The reaction of *N*-allenyl-2-pyrrolidinone **1a** (0.2 mmol, 1 equiv.) with diphenyl phosphite **2d** (0.24 mmol, 1.2 equiv.) afforded the corresponding hydrophosphorylation product **3ad** as a yellow oil according to the general procedure (flash chromatography: AcOEt).

$^{1}H\ \{^{31}P\}$ NMR (400 MHz, $CDCl_3$) δ = 7.33 – 7.27 (m, 4H), 7.19 – 7.13 (m, 6H), 7.05 (d, *J* = 14.4, 1H), 4.94 (dt, *J* = 14.4, 7.6 Hz, 1H), 3.47 (t, *J* = 7.3 Hz, 2H), 2.93 (dd, *J* = 7.6, 1.3 Hz, 2H), 2.47 (t, *J* = 8.7 Hz, 2H), 2.13 – 2.04 (m, 2H).

^{13}C NMR $\{^{1}H\}$ (101 MHz, $CDCl_3$) δ = 173.17, 150.47 (d, *J* = 9.1 Hz), 129.88, 128.42 (d, *J* = 16.5 Hz), 125.29, 120.63 (d, *J* = 4.4 Hz), 98.86 (d, *J* = 11.7 Hz), 45.14, 31.14, 28.41 (d, *J* = 142.6 Hz), 17.51.

$^{31}P\ \{^{1}H\}$ NMR (162 MHz, $CDCl_3$) δ = 20.14.

HRMS (ESI) *m/z*: $[M+H]^+$ calcd. for $C_{19}H_{21}NO_4P$ 358.1203, found 358.1206.

6.5. *Dibenzyl (E)-(3-(2-oxopyrrolidin-1-yl)allyl)phosphonate 3ae*

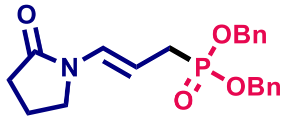

3ae

The reaction of *N*-allenyl-2-pyrrolidinone **1a** (0.2 mmol, 1 equiv.) with dibenzyl phosphite **2e** (0.24 mmol, 1.2 equiv.) afforded the corresponding hydrophosphorylation product **3ae** as a light yellow solid according to the general procedure (flash chromatography: AcOEt/iPrOH, 95:5).

$^{1}H\ \{^{31}P\}$ NMR (400 MHz, $CDCl_3$) δ = 7.33 – 7.27 (m, 10H), 6.86 (d, *J* = 14.4 Hz, 1H), 5.07 – 4.90 (m, 4H), 4.75 (dt, *J* = 14.7, 7.6 Hz, 1H), 3.35 (t, *J* = 7.2 Hz, 2H), 2.56 (dd, *J* = 7.6, 1.2 Hz, 2H), 2.41 (t, *J* = 8.4 Hz, 2H), 2.02 (p, *J* = 7.7 Hz, 2H).

^{13}C NMR $\{^{1}H\}$ (101 MHz, $CDCl_3$) δ = 172.80, 136.27 (d, *J* = 5.6 Hz), 128.53, 128.37, 127.93, 127.51 (d, *J* = 15.9 Hz), 99.71 (d, *J* = 11.2 Hz), 67.47 (d, *J* = 6.7 Hz), 44.94, 31.02, 28.36 (d, *J* = 142.0 Hz), 17.32.

$^{31}P\ \{^{1}H\}$ NMR (162 MHz, $CDCl_3$) δ = 28.50.

HRMS (ESI) *m/z*: $[M+H]^+$ calcd. for $C_{21}H_{25}NO_4P$ 386.1520, found 386.1516.

6.6. *Diethyl (E)-(3-(2-oxooxazolidin-3-yl)allyl)phosphonate 3ba*

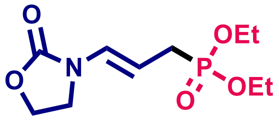

3ba

The reaction of *N*-allenyl-2-oxazolidinone **1b** (0.2 mmol, 1 equiv.) with diethyl phosphite **2a** (0.24 mmol, 1.2 equiv.) afforded the corresponding hydrophosphorylation product **3ba** as a yellow oil according to the general procedure (flash chromatography: AcOEt/iPrOH, 90:10).

$^{1}H\ \{^{31}P\}$ NMR (400 MHz, $CDCl_3$) δ = 6.67 (d, *J* = 14.3 Hz, 1H), 4.69 (dt, *J* = 14.3, 7.6 Hz, 1H), 4.38 – 4.33 (m, 2H), 4.01 (qd, *J* = 7.0, 1.5 Hz, 4H), 3.67 – 3.61 (m, 2H), 2.49 (dd, *J* = 7.6, 1.3 Hz, 2H), 1.22 (t, *J* = 7.1 Hz, 6H).

^{13}C NMR $\{^{1}H\}$ (101 MHz, $CDCl_3$) δ = 155.12, 127.42 (d, *J* = 15.4 Hz), 99.33 (d, *J* = 11.0 Hz), 62.20, 61.94 (d, *J* = 6.8 Hz), 42.36, 27.52 (d, *J* = 142.8 Hz), 16.37 (d, *J* = 5.9 Hz).

$^{31}P\ \{^{1}H\}$ NMR (162 MHz, $CDCl_3$) δ = 27.02.

HRMS (ESI) *m/z*: $[M+H]^+$ calcd. for $C_{10}H_{19}NO_5P$ 264.0995, found 264.1004.

6.7. *Dimethyl (E)-(3-(2-oxooxazolidin-3-yl)allyl)phosphonate 3bb*

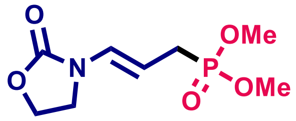

3bb

The reaction of *N*-allenyl-2-oxazolidinone **1b** (0.2 mmol, 1 equiv.) with diethyl phosphite **2b** (0.24 mmol, 1.2 equiv.) afforded the corresponding hydrophosphorylation product **3bb** as a pale yellow oil according to the general procedure (flash chromatography: AcOEt/iPrOH, 95:5).

^{1}H {^{31}P} NMR (400 MHz, $CDCl_3$) δ = 6.74 (d, *J* = 14.3 Hz, 1H), 4.73 (dt, *J* = 14.3, 7.6 Hz, 1H), 4.45 – 4.38 (m, 2H), 3.71 (s, 6H), 3.71 – 3.66 (m, 2H) 2.57 (dd, *J* = 7.6, 1.3 Hz, 2H).

^{13}C NMR {^{1}H} (101 MHz, $CDCl_3$) δ = 155.22, 127.75 (d, *J* = 15.6 Hz), 99.02 (d, *J* = 11.1 Hz), 62.30, 52.82 (d, *J* = 6.8 Hz), 42.46, 26.69 (d, *J* = 143.2 Hz).

^{31}P {^{1}H} NMR (162 MHz, $CDCl_3$) δ = 29.48.

HRMS (ESI) *m/z*: $[M+H]^+$ calcd. for $C_8H_{15}NO_5P$ 236.0682, found 236.0683.

6.8. *Diisopropyl (E)-(3-(2-oxooxazolidin-3-yl)allyl)phosphonate 3bc*

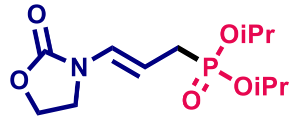

3bc

The reaction of *N*-allenyl-2-oxazolidinone **1b** (0.2 mmol, 1 equiv.) with diisopropyl phosphite **2c** (0.24 mmol, 1.2 equiv.) afforded the corresponding hydrophosphorylation product **3bc** as an off-white solid according to the general procedure (flash chromatography: AcOEt/iPrOH, 90:10).

^{1}H {^{31}P} NMR (400 MHz, $CDCl_3$) δ = 6.74 (d, *J* = 14.3 Hz, 1H), 4.77 (dt, *J* = 14.3, 7.6 Hz, 1H), 4.67 (hept, *J* = 6.2 Hz, 2H), 4.45 – 4.40 (m, 2H), 3.72 – 3.66 (m, 2H), 2.52 (dd, *J* = 7.6, 1.3 Hz, 2H), 1.29 (t, *J* = 6.1 Hz, 12H).

^{13}C NMR {^{1}H} (101 MHz, $CDCl_3$) δ = 155.28, 127.41 (d, *J* = 15.6 Hz), 100.12 (d, *J* = 11.0 Hz), 70.57 (d, *J* = 6.9 Hz), 62.29, 42.56, 28.99 (d, *J* = 144.1 Hz), 24.15 (d, *J* = 4.4 Hz).

^{31}P {^{1}H} NMR (162 MHz, $CDCl_3$) δ = 25.04.

HRMS (ESI) *m/z*: $[M+H]^+$ calcd. for $C_{12}H_{23}NO_5P$ 292.1308, found 292.1317.

6.9. *Diphenyl (E)-(3-(2-oxooxazolidin-3-yl)allyl)phosphonate 3bd*

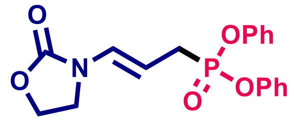

3bd

The reaction of *N*-allenyl-2-oxazolidinone **1b** (0.2 mmol, 1 equiv.) with diphenyl phosphite **2d** (0.24 mmol, 1.2 equiv.) afforded the corresponding hydrophosphorylation product **3bd** as a pale yellow oil according to the general procedure (flash chromatography: AcOEt/hexane, 75:25).

^{1}H {^{31}P} NMR (400 MHz, $CDCl_3$) δ = 7.35 – 7.28 (m, 4H), 7.20 – 7.13 (m, 6H), 6.84 (d, *J* = 14.2 Hz, 1H), 4.83 (dt, *J* = 14.7, 7.6 Hz, 1H), 4.45 – 4.38 (m, 2H), 3.68 – 3.62 (m, 2H), 2.93 (dd, *J* = 7.6, 1.3 Hz, 2H).

^{13}C NMR {^1H} (101 MHz, $CDCl_3$) δ = 155.19, 150.44 (d, J = 9.1 Hz), 129.92, 128.72 (d, J = 16.3 Hz), 125.38, 120.58, 97.98 (d, J = 11.6 Hz), 62.34, 42.44, 28.11 (d, J = 143.2 Hz).

^{31}P {^1H} NMR (162 MHz, $CDCl_3$) δ = 19.81.

HRMS (ESI) *m/z*: $[M+H]^+$ calcd. for $C_{18}H_{19}NO_5P$ 360.0995, found 360.0999.

6.10. *Dibenzyl (E)-(3-(2-oxooxazolidin-3-yl)allyl)phosphonate 3be*

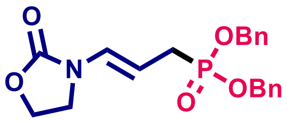

3be

The reaction of *N*-allenyl-2-oxazolidinone **1b** (0.2 mmol, 1 equiv.) with dibenzyl phosphite **2e** (0.24 mmol, 1.2 equiv.) afforded the corresponding hydrophosphorylation product **3be** as an off-white solid according to the general procedure (flash chromatography: AcOEt/iPrOH, 98:2).

^1H {^{31}P} NMR (400 MHz, $CDCl_3$) δ = 7.35 – 7.27 (m, 10H), 6.62 (d, J = 14.3 Hz, 1H), 5.06 – 4.93 (m, 4H), 4.62 (dt, J = 14.3, 7.6 Hz, 1H), 4.37 – 4.33 (m, 2H), 3.54 – 3.49 (m, 2H), 2.55 (dd, J = 7.6, 1.2 Hz, 2H).

^{13}C NMR {^1H} (101 MHz, $CDCl_3$) δ = 155.07, 136.25 (d, J = 5.5 Hz), 128.61, 128.48, 128.03, 127.80 (d, J = 15.7 Hz), 98.81 (d, J = 11.1 Hz), 67.60 (d, J = 6.7 Hz), 62.20, 42.28, 28.08 (d, J = 142.5 Hz).

^{31}P {^1H} NMR (162 MHz, $CDCl_3$) δ = 28.59.

HRMS (ESI) *m/z*: $[M+H]^+$ calcd. for $C_{20}H_{23}NO_5P$ 388.1308, found 388.1311.

6.11. *Diethyl (E)-(3-((4-methyl-N-phenylphenyl)sulfonamido)allyl)phosphonate 3ca*

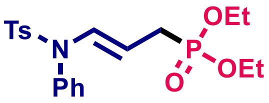

3ca

The reaction of *N*-phenyl-*N*-allenyl-*p*-toluenesulfonamide **1c** (0.2 mmol, 1 equiv.) with diethyl phosphite **2a** (0.24 mmol, 1.2 equiv.) afforded the corresponding hydrophosphorylation product **3ca** as a pale yellow oil according to the general procedure (flash chromatography: AcOEt).

^1H {^{31}P} NMR (400 MHz, $CDCl_3$) δ = 7.56 – 7.53 (m, 2H), 7.36 – 7.30 (m, 3H), 7.27 – 7.24 (m, 2H), 7.09 (dt, J = 14.0, 1.2 Hz, 1H), 6.97 – 6.93 (m, 2H), 4.32 (dt, J = 13.9, 7.7 Hz, 1H), 4.01 (q, J = 7.1 Hz, 4H), 2.47 (dd, J = 7.7, 1.2 Hz, 2H), 2.42 (s, 3H), 1.24 (t, J = 7.1 Hz, 6H).

^{13}C NMR {^1H} (101 MHz, $CDCl_3$) δ = 144.11, 136.40, 135.89, 132.69, 132.54, 130.23, 129.69 (d, J = 7.3 Hz), 129.22, 127.65, 100.59 (d, J = 11.0 Hz), 62.04 (d, J = 6.7 Hz), 27.77 (d, J = 142.6 Hz), 21.73, 16.57 (d, J = 5.9 Hz).

^{31}P {^1H} NMR (162 MHz, $CDCl_3$) δ = 27.03.

HRMS (ESI) *m/z*: $[M+H]^+$ calcd. for $C_{20}H_{27}NO_5PS$ 424.1342, found 424.1343.

6.12. *Dimethyl (E)-(3-((4-methyl-N-phenylphenyl)sulfonamido)allyl)phosphonate 3cb*

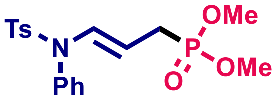

3cb

The reaction of *N*-phenyl-*N*-allenyl-*p*-toluenesulfonamide **1c** (0.2 mmol, 1 equiv.) with dimethyl phosphite **2b** (0.24 mmol, 1.2 equiv.) afforded the corresponding hydrophosphorylation product **3cb** as a pale yellow solid according to the general procedure (flash chromatography: AcOEt).

^{1}H {^{31}P} NMR (400 MHz, $CDCl_3$) δ = 7.54 – 7.51 (m, 2H), 7.35 – 7.30 (m, 3H), 7.27 – 7.24 (m, 2H), 7.10 (dt, *J* = 14.0, 1.2 Hz, 1H), 6.95 – 6.92 (m, 2H), 4.30 (dt, *J* = 13.9, 7.7 Hz, 1H), 3.65 (s, 6H), 2.48 (dd, *J* = 7.7, 1.2 Hz, 2H), 2.41 (s, 3H).

^{13}C NMR {^{1}H} (101 MHz, $CDCl_3$) δ = 144.14, 136.27, 135.75, 132.82 (d, *J* = 15.5 Hz), 130.15, 129.70, 129.65, 129.23, 127.60, 99.98 (d, *J* = 11.0 Hz), 52.81 (d, *J* = 6.9 Hz), 26.84 (d, *J* = 142.7 Hz), 21.69.

^{31}P {^{1}H} NMR (162 MHz, $CDCl_3$) δ = 29.44.

HRMS (ESI) *m/z*: $[M+H]^+$ calcd. for $C_{18}H_{23}NO_5PS$ 396.1029, found 396.1033.

6.13. *Diisopropyl (E)-(3-((4-methyl-N-phenylphenyl)sulfonamido)allyl)phosphonate 3cc*

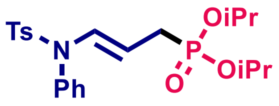

3cc

The reaction of *N*-phenyl-*N*-allenyl-*p*-toluenesulfonamide **1c** (0.2 mmol, 1 equiv.) with diisopropyl phosphite **2c** (0.24 mmol, 1.2 equiv.) afforded the corresponding hydrophosphorylation product **3cc** as a pale yellow oil according to the general procedure (flash chromatography: AcOEt).

^{1}H {^{31}P} NMR (400 MHz, $CDCl_3$) δ =7.56 – 7.53 (m, 2H), 7.35 – 7.30 (m, 3H), 7.27 – 7.24 (m, 2H), 7.07 (dt, *J* = 13.9, 1.2 Hz, 1H), 6.97 – 6.94 (m, 2H), 4.58 (hept, *J* = 6.2 Hz, 2H), 4.31 (dt, *J* = 14.0, 7.7 Hz, 1H), 2.44 – 2.41 (m, 5H), 1.22 (dd, *J* = 20.1, 6.2 Hz, 12H).

^{13}C NMR {^{1}H} (101 MHz, $CDCl_3$) δ = 144.05, 136.47, 135.96, 132.39 (d, *J* = 15.6 Hz), 130.27, 129.67 (d, *J* = 10.1 Hz), 129.18, 127.67, 101.20 (d, *J* = 11.0 Hz), 70.38 (d, *J* = 6.9 Hz), 28.82 (d, *J* = 143.8 Hz), 24.14 (m – 2C), 21.73.

^{31}P {^{1}H} NMR (162 MHz, $CDCl_3$) δ = 25.15.

HRMS (ESI) *m/z*: $[M+H]^+$ calcd. for $C_{22}H_{31}NO_5PS$ 452.1655, found 452.1657.

6.14. *Diphenyl (E)-(3-((4-methyl-N-phenylphenyl)sulfonamido)allyl)phosphonate 3cd*

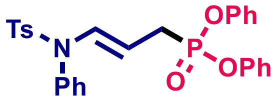

3cd

The reaction of *N*-phenyl-*N*-allenyl-*p*-toluenesulfonamide **1c** (0.2 mmol, 1 equiv.) with diphenyl phosphite **2d** (0.24 mmol, 1.2 equiv.) afforded the corresponding hydrophosphorylation product **3cd** as a yellow solid according to the general procedure (flash chromatography: hexane/AcOEt, 75:25).

^{1}H {^{31}P} NMR (400 MHz, $CDCl_3$) δ = 7.55 – 7.51 (m, 2H), 7.38 – 7.26 (m, 7H), 7.23 – 7.13 (m, 5H), 7.10 – 7.06 (m, 4H), 6.93 – 6.89 (m, 2H), 4.38 (dt, *J* = 13.9, 7.7 Hz, 1H), 2.83 (dd, *J* = 7.7, 1.2 Hz, 2H), 2.40 (s, 3H).

^{13}C NMR {^{1}H} (101 MHz, $CDCl_3$) δ = 150.42 (d, *J* = 9.1 Hz), 144.13, 136.14, 135.70, 133.74 (d, *J* = 16.2 Hz), 130.16, 129.84 (2C), 129.73 (d, *J* = 5.5 Hz), 129.30, 127.60, 125.24 (d, *J* = 1.2 Hz), 120.57 (d, *J* = 4.4 Hz), 98.75 (d, *J* = 11.6 Hz), 27.91 (d, *J* = 142.6 Hz), 21.70.

^{31}P {^{1}H} NMR (162 MHz, $CDCl_3$) δ = 20.04.

HRMS (ESI) *m/z*: $[M+H]^+$ calcd. for $C_{28}H_{27}NO_5PS$ 520.1342, found 520.1334.

6.15. *Dibenzyl (E)-(3-((4-methyl-N-phenylphenyl)sulfonamido)allyl)phosphonate 3ce*

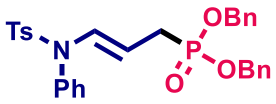

3ce

The reaction of *N*-phenyl-*N*-allenyl-*p*-toluenesulfonamide **1c** (0.2 mmol, 1 equiv.) with dibenzyl phosphite **2e** (0.24 mmol, 1.2 equiv.) afforded the corresponding hydrophosphorylation product **3ce** as a pale yellow oil according to the general procedure (flash chromatography: hexane/AcOEt, 60:40).

^{1}H {^{31}P} NMR (400 MHz, $CDCl_3$) δ = 7.52 – 7.48 (m, 2H), 7.38 – 7.23 (m, 13H), 7.21 – 7.17 (m, 2H), 7.08 (dt, *J* = 13.9, 1.3 Hz, 1H), 6.92 – 6.88 (m, 2H), 5.00 – 4.87 (m, 4H), 4.29 (dt, *J* = 14.0, 7.7 Hz, 1H), 2.49 (dd, *J* = 7.7, 1.2 Hz, 2H), 2.40 (s, 3H).

^{13}C NMR {^{1}H} (101 MHz, $CDCl_3$) δ = 144.04, 136.41, 136.35, 136.34, 135.82, 132.96, 132.81, 130.20, 129.67 (d, *J* = 7.9 Hz), 128.70, 128.50, 127.88, 127.60, 100.15 (d, *J* = 11.3 Hz), 67.52 (d, *J* = 6.7 Hz), 28.08 (d, *J* = 141.9 Hz), 21.70.

^{31}P {^{1}H} NMR (162 MHz, $CDCl_3$) δ = 28.05.

HRMS (ESI) *m/z*: $[M+H]^+$ calcd. for $C_{30}H_{31}NO_5PS$ 548.1655, found 548.1655.

6.16. *(E)-1-(3-(diphenylphosphoryl)prop-1-en-1-yl)pyrrolidin-2-one 5aa*

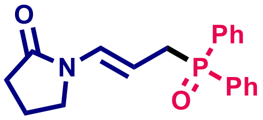

5aa

The reaction of *N*-allenyl-2-pyrrolidinone **1a** (0.2 mmol, 1 equiv.) with diphenylphosphine oxide **4a** (0.24 mmol, 1.2 equiv.) afforded the corresponding hydrophosphorylation product **5aa** as an off-white solid according to the general procedure (flash chromatography: AcOEt/iPrOH, 85:15).

^{1}H {^{31}P} NMR (400 MHz, $CDCl_3$) δ = 7.74 – 7.68 (m, 4H), 7.54 – 7.42 (m, 6H), 6.89 (d, *J* = 14.4 Hz, 1H), 4.96 (dt, *J* = 14.8, 7.6 Hz, 1H), 3.41 (t, *J* = 7.2 Hz, 2H), 3.13 (dd, *J* = 7.6, 1.3 Hz, 2H), 2.41 (t, *J* = 8.2 Hz, 2H), 2.06 – 1.96 (m, 2H).

^{13}C NMR {^{1}H} (101 MHz, $CDCl_3$) δ = 173.03, 132.96, 132.01 (d, *J* = 2.9 Hz), 131.03 (d, *J* = 9.1 Hz), 128.79 (d, *J* = 11.7 Hz), 127.87 (d, *J* = 12.7 Hz), 99.97 (d, *J* = 8.9 Hz), 45.22, 32.64 (d, *J* = 70.7 Hz), 31.18, 17.44.

^{31}P {^{1}H} NMR (162 MHz, $CDCl_3$) δ = 30.14.

HRMS (ESI) *m/z*: $[M+H]^+$ calcd. for $C_{19}H_{21}NO_2P$ 326.1304, found 326.1318.

6.17. *(E)-3-(3-(diphenylphosphoryl)prop-1-en-1-yl)oxazolidin-2-one 5ba*

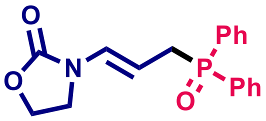

5ba

The reaction of *N*-allenyl-2-oxazolidinone **1b** (0.2 mmol, 1 equiv.) with diphenylphosphine oxide **4a** (0.24 mmol, 1.2 equiv.) afforded the corresponding hydrophosphorylation product **5ba** as an off-white solid according to the general procedure (flash chromatography: AcOEt/iPrOH, 85:15).

^{1}H {^{31}P} NMR (400 MHz, $CDCl_3$) δ = 7.74 – 7.69 (m, 4H), 7.56 – 7.45 (m, 6H), 6.66 (d, *J* = 14.3 Hz, 1H), 4.87 (dt, *J* = 14.7, 7.6 Hz, 1H), 4.40 – 4.34 (m, 2H), 3.65 – 3.59 (m, 2H), 3.13 (dd, *J* = 7.6, 1.3 Hz, 2H).

^{13}C NMR {^{1}H} (101 MHz, $CDCl_3$) δ = 155.21, 132.33 (d, *J* = 95.5 Hz), 132.13 (d, *J* = 2.8 Hz), 131.02 (d, *J* = 9.2 Hz), 128.87 (d, *J* = 11.7 Hz), 128.11 (d, *J* = 12.4 Hz), 99.04 (d, *J* = 8.9 Hz), 62.31, 42.54, 32.30 (d, *J* = 70.6 Hz).

^{31}P {^{1}H} NMR (162 MHz, $CDCl_3$) δ = 30.15.

HRMS (ESI) *m/z*: $[M+H]^+$ calcd. for $C_{18}H_{19}NO_3P$ 328.1097, found 328.1103.

6.18. *(E)-N-(3-(diphenylphosphoryl)prop-1-en-1-yl)-4-methyl-N-phenylbenzenesulfonamide 5ca*

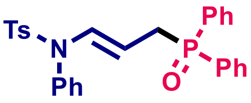

5ca

The reaction of *N*-phenyl-*N*-allenyl-*p*-toluenesulfonamide **1c** (0.2 mmol, 1 equiv.) with diphenylphosphine oxide **4a** (0.24 mmol, 1.2 equiv.) afforded the corresponding hydrophosphorylation product **5ca** as an off-white solid according to the general procedure (flash chromatography: AcOEt/hexane, 75:25).

^{1}H {^{31}P} NMR (400 MHz, $CDCl_3$) δ = 7.70 – 7.63 (m, 4H), 7.56 – 7.51 (m, 2H), 7.49 – 7.42 (m, 4H), 7.41 – 7.36 (m, 2H), 7.32 – 7.20 (m, 5H), 7.00 (d, *J* = 14.0 Hz, 1H), 6.80 – 6.75 (m, 2H), 4.35 (dt, *J* = 14.1, 7.8 Hz, 1H), 3.02 (dd, *J* = 7.8, 1.2 Hz, 2H), 2.45 (s, 3H).

^{13}C NMR {^{1}H} (101 MHz, $CDCl_3$) δ = 143.80, 136.68 (d, *J* = 46.8 Hz), 132.26 (d, *J* = 100.1 Hz), 132.22 (d, *J* = 2.8 Hz), 131.76, 130.91 (d, *J* = 9.5 Hz), 129.69, 129.37, 128.96 (d, *J* = 11.8 Hz), 128.70 (d, *J* = 11.1 Hz), 127.37, 125.27, 121.70, 36.04, 21.89 (d, *J* = 73.6 Hz), 21.66.

^{31}P {^{1}H} NMR (162 MHz, $CDCl_3$) δ = 30.19.

HRMS (ESI) *m/z*: $[M+H]^+$ calcd. for $C_{28}H_{27}NO_3PS$ 488.1449, found 488.1447.

6.19. *Ethyl (E)-(3-(2-oxopyrrolidin-1-yl)allyl)(phenyl)phosphinate 7aa*

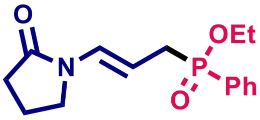

7aa

The reaction of *N*-allenyl-2-pyrrolidinone **1a** (0.2 mmol, 1 equiv.) with ethyl phenylphosphinate **6a** (0.24 mmol, 1.2 equiv.) afforded the corresponding hydrophosphorylation product **7aa** as a yellow oil according to the general procedure (flash chromatography: AcOEt/iPrOH, 85:15).

^{1}H {^{31}P} NMR (400 MHz, acetone-*d*$_6$) δ = 7.81 – 7.77 (m, 2H), 7.61 – 7.57 (m, 1H), 7.54 – 7.50 (m, 2H), 6.81 (dd, J = 14.4, 1.4 Hz, 1H), 4.79 (dt, J = 14.7, 7.6 Hz, 1H), 4.02 (dq, J = 10.3, 7.0 Hz, 1H), 3.87 (dq, J = 10.3, 7.0 Hz, 1H), 3.47 – 3.40 (m, 2H), 2.79 (dd, J = 7.7, 1.3 Hz, 2H), 2.35 – 2.28 (m, 2H), 2.10 – 2.06 (m, 2H), 1.25 (t, J = 7.0 Hz, 3H).

^{13}C NMR {^{1}H} (101 MHz, acetone-*d*$_6$) δ = 172.86, 133.15, 132.88 (d, *J* = 2.8 Hz), 132.57 (d, *J* = 9.4 Hz), 131.92, 129.27 (d, *J* = 12.2 Hz), 128.34, 128.21, 61.04, 60.97, 45.49, 18.03, 16.84 (d, *J* = 5.9 Hz).

^{31}P {^{1}H} NMR (162 MHz, acetone-*d*$_6$) δ = 38.63.

HRMS (ESI) *m/z*: $[M+H]^+$ calcd. for $C_{15}H_{21}NO_3P$ 294.1254, found 294.1258.

7. Kinetic studies

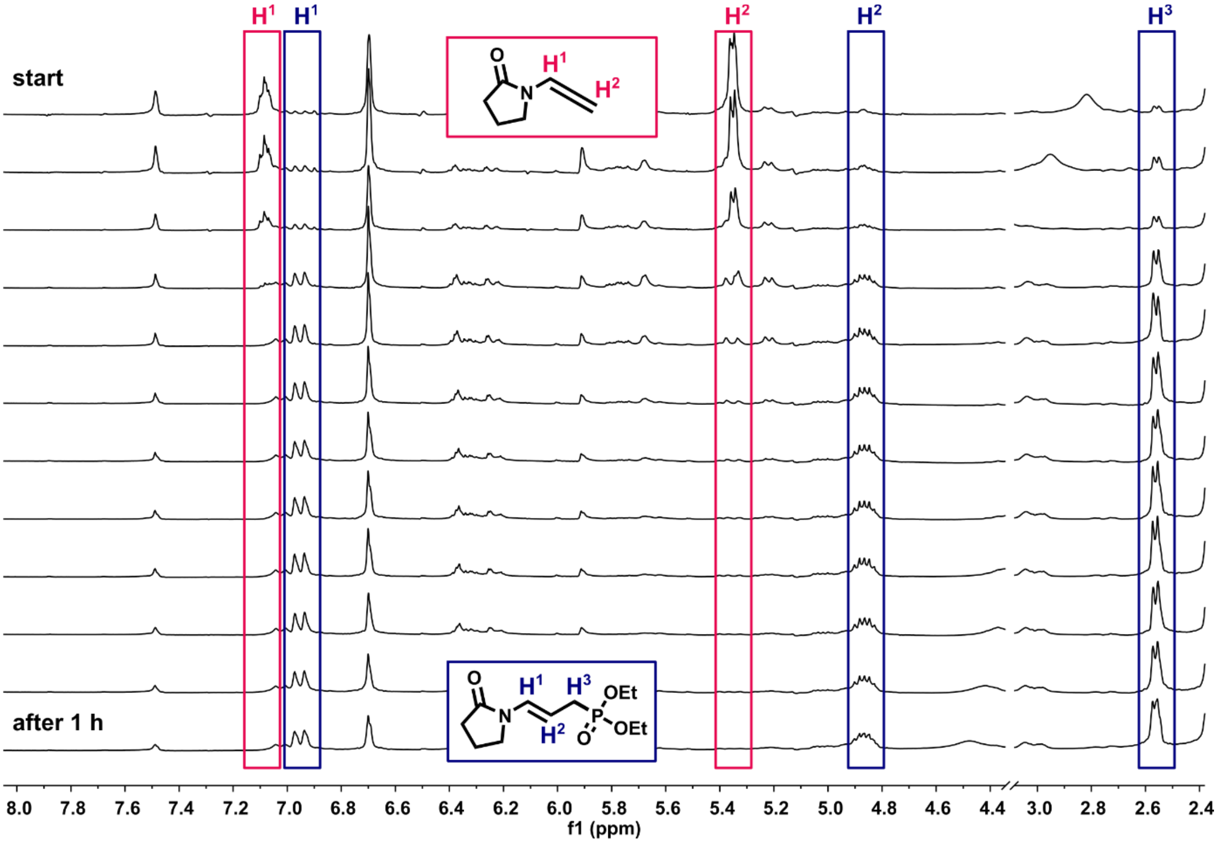

Figure S1. ^{1}H {^{31}P} NMR spectra (400 MHz, dioxane-d_8) showing the progress of the hydrophosphorylation reaction between *N*-allenyl-2-pyrrolidinone **1a** and diethyl phosphite **2a**.

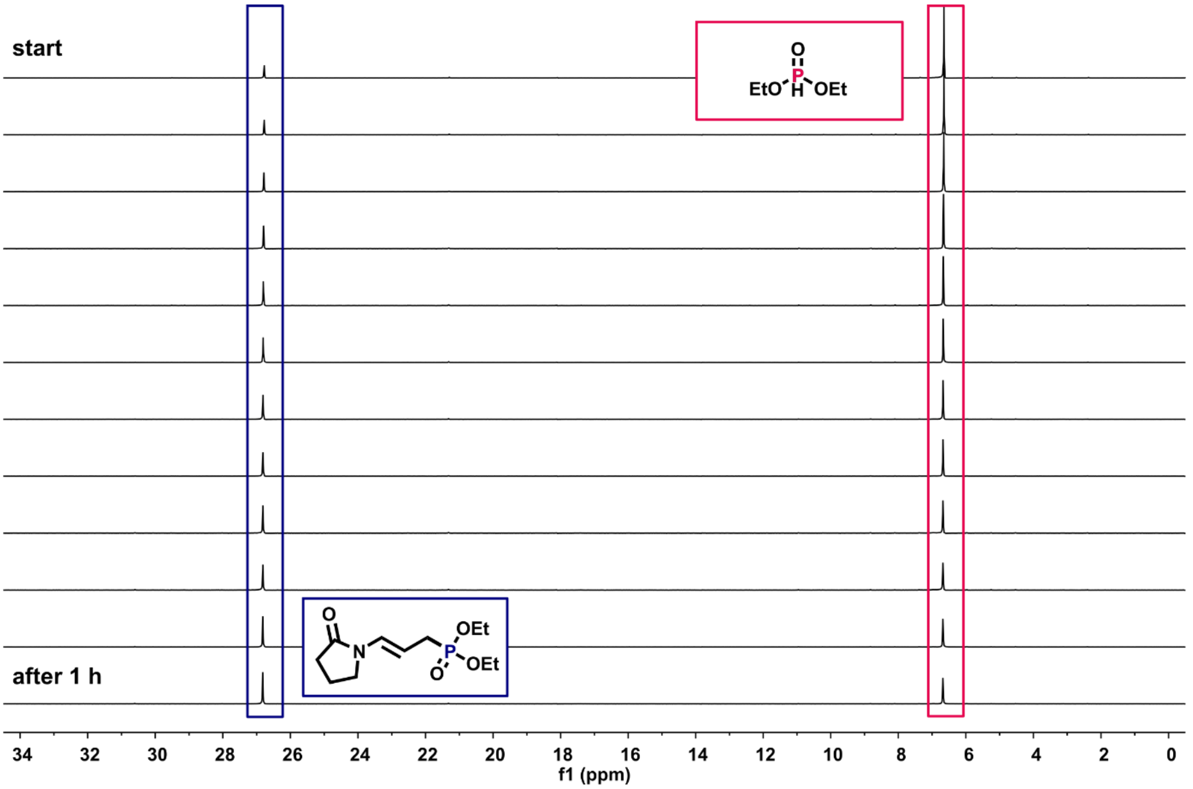

Figure S2. ^{31}P {^{1}H} NMR spectra (162 MHz, dioxane-d_8) showing the progress of the hydrophosphorylation reaction between *N*-allenyl-2-pyrrolidinone **1a** and diethyl phosphite **2a**.

8. Computational details

All DFT calculations were performed with the Amsterdam Density Functional (ADF),[1,2] and QUILD[3] programs. Molecular orbitals were expanded in an uncontracted set of Slater-type orbitals (STOs) of triple-ζ quality with double polarization functions (TZ2P).[4,5] Core electrons were not treated explicitly during the geometry optimizations (frozen core approximation[2]). An auxiliary set of s, p, d, f, and g STOs was used to fit the molecular density and to represent the Coulomb and exchange potentials accurately for each SCF cycle.

Geometries of all possible spin states were optimized with the QUILD[3] program using adapted delocalized coordinates until the maximum gradient component was less than 10^{-4} a.u. Energies, gradients, Hessians[6] (for vibrational frequencies) were calculated using S12g,[7] in all cases by including solvation effects through the COSMO[8] dielectric continuum model with appropriate parameters for the solvents.[9] For computing Gibbs free energies, all small frequencies were raised to 100 cm^{-1} in order to compensate for the breakdown of the harmonic oscillator model.[10,11] Scalar relativistic corrections have been included self-consistently in all calculations by using the zeroth-order regular approximation (ZORA).[12] Most S12g calculations were performed with a Becke grid[13,14] of VeryGood quality, except the frequencies which were computed with a Normal grid. All DFT calculations were performed using the unrestricted Kohn-Sham scheme.

All computational data have been uploaded onto the IOCHEM-BD platform (www.iochem-bd.org), DOI: https://doi.org/10.19061/iochem-bd-4-74, to facilitate data exchange and dissemination, according to the FAIR principles[15] of OpenData sharing.

9. NMR spectra

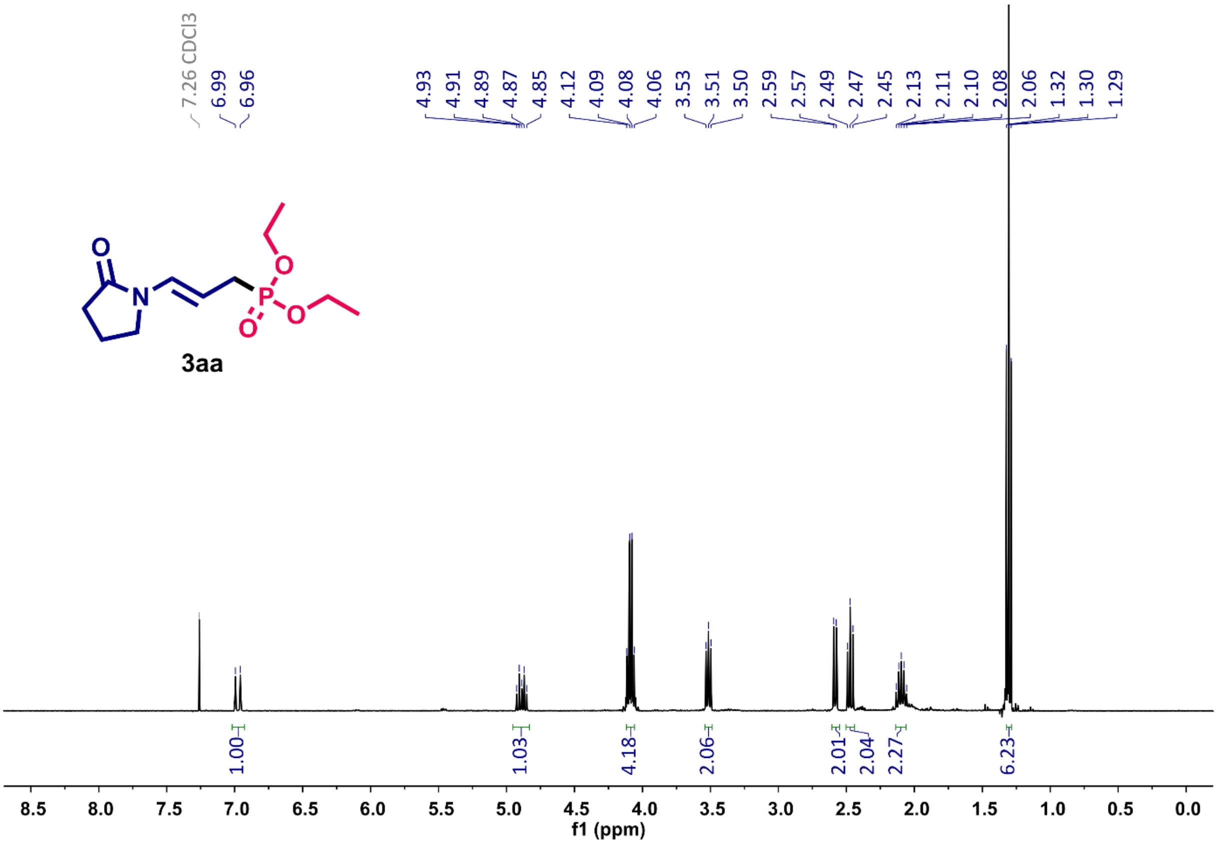

Figure S3. $^{1}H\{^{31}P\}$ NMR spectrum (400 MHz, $CDCl_3$) of *diethyl (E)-(3-(2-oxopyrrolidin-1-yl)allyl)phosphonate* **3aa**.

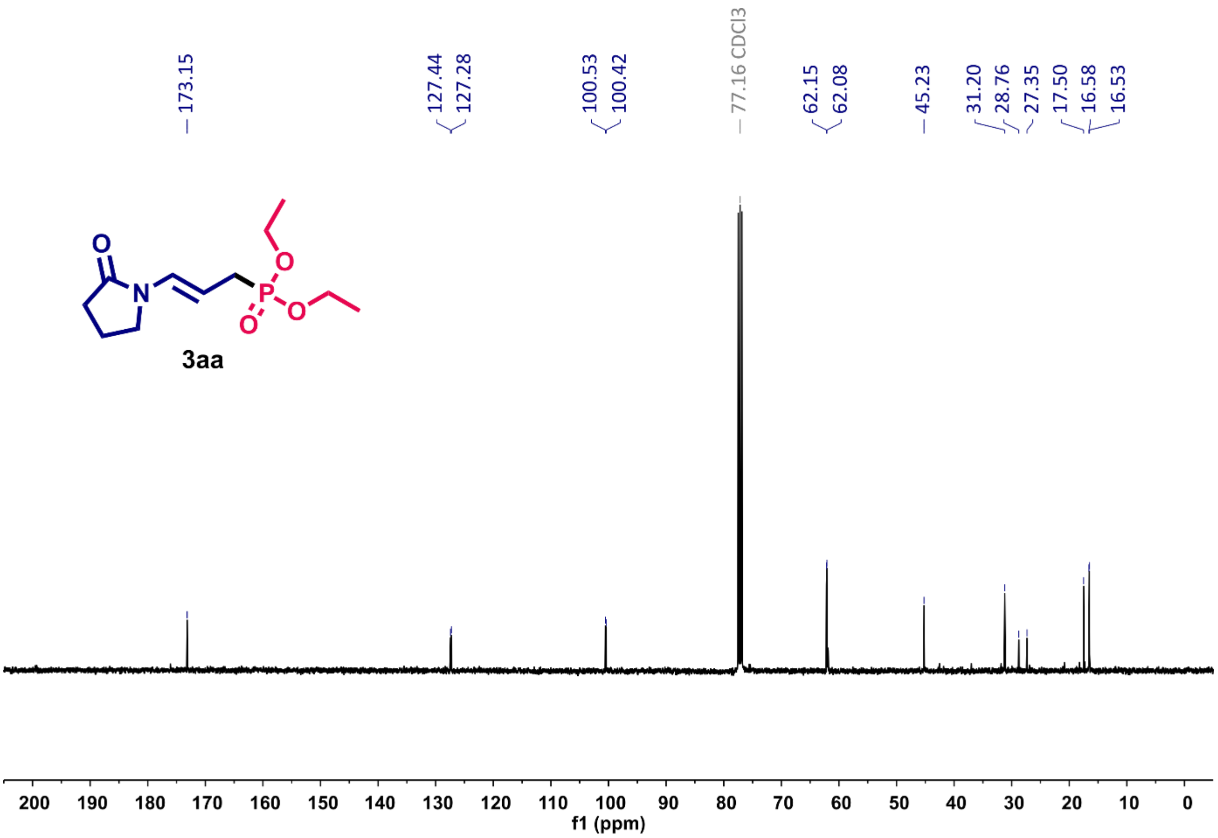

Figure S4. ^{13}C {^{1}H} NMR spectrum (101 MHz, $CDCl_3$) of *diethyl (E)-(3-(2-oxopyrrolidin-1-yl)allyl)phosphonate* **3aa**.

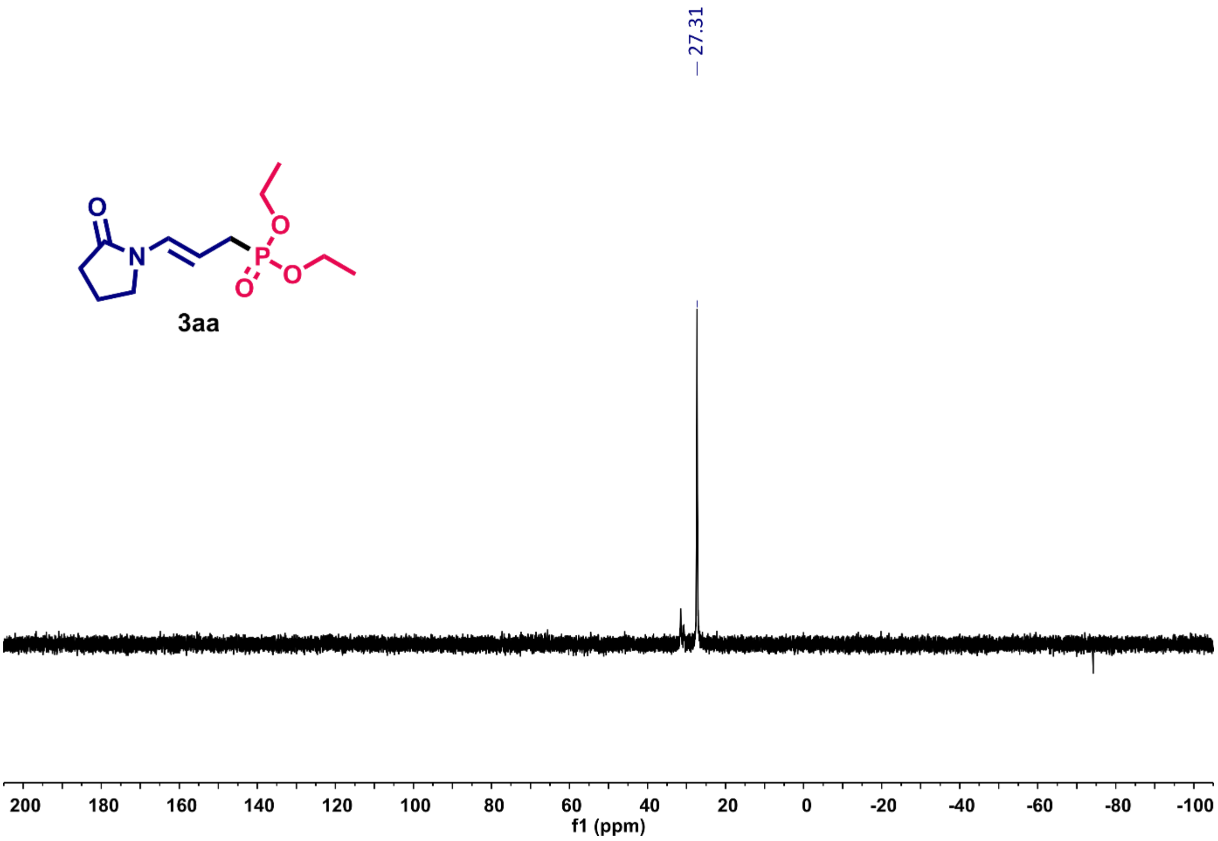

Figure S5. ^{31}P {^{1}H} NMR spectrum (162 MHz, $CDCl_3$) of *diethyl (E)-(3-(2-oxopyrrolidin-1-yl)allyl)phosphonate* **3aa**.

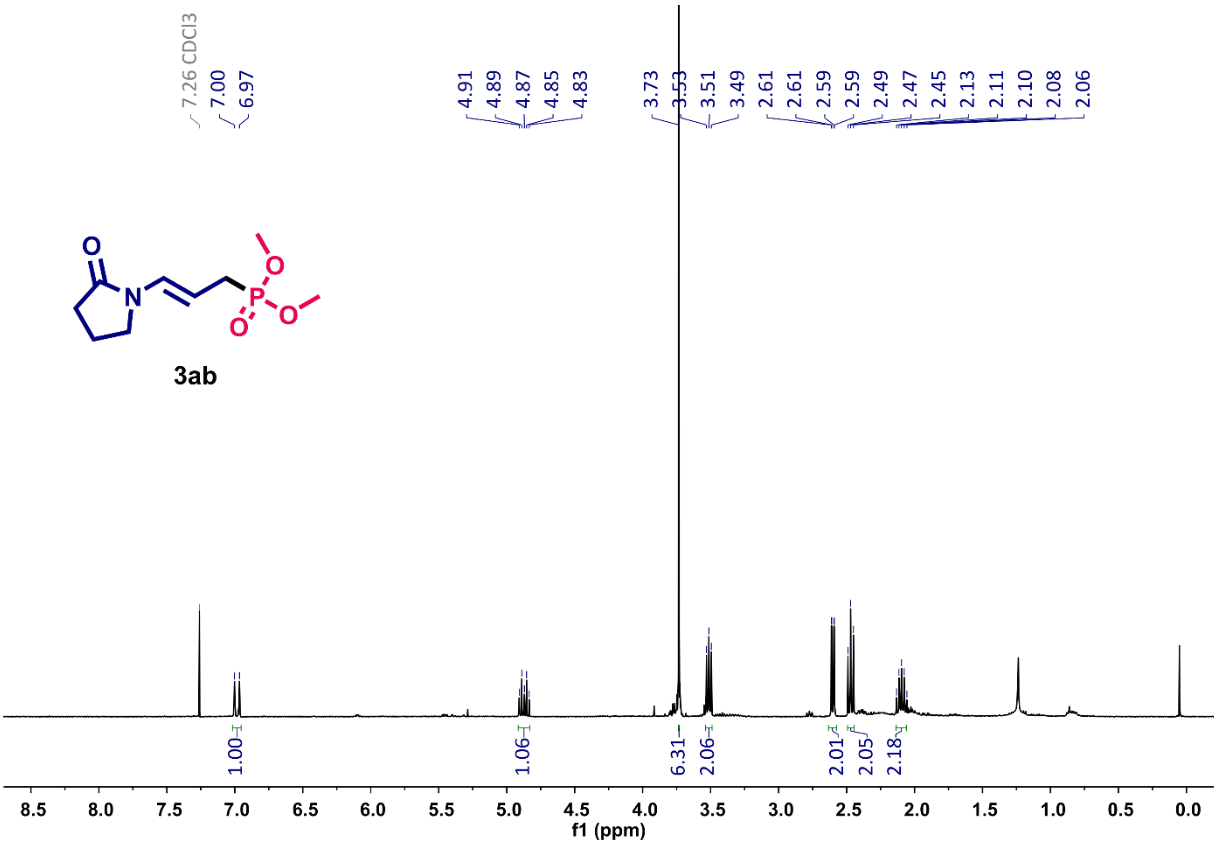

Figure S6. $^{1}H\{^{31}P\}$ NMR spectrum (400 MHz, $CDCl_3$) of *dimethyl (E)-(3-(2-oxopyrrolidin-1-yl)allyl)phosphonate* **3ab**.

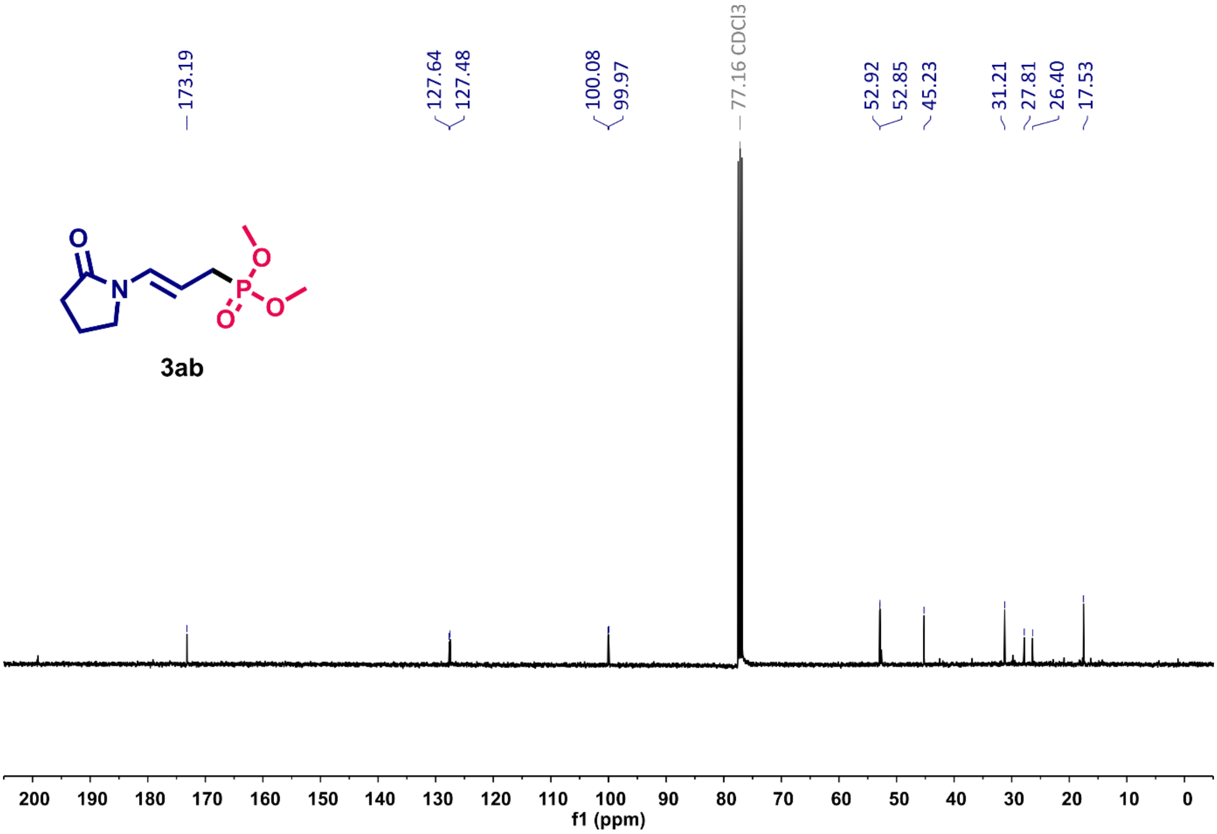

Figure S7. $^{13}C\{^{1}H\}$ NMR spectrum (101 MHz, $CDCl_3$) of *dimethyl (E)-(3-(2-oxopyrrolidin-1-yl)allyl)phosphonate* **3ab**.

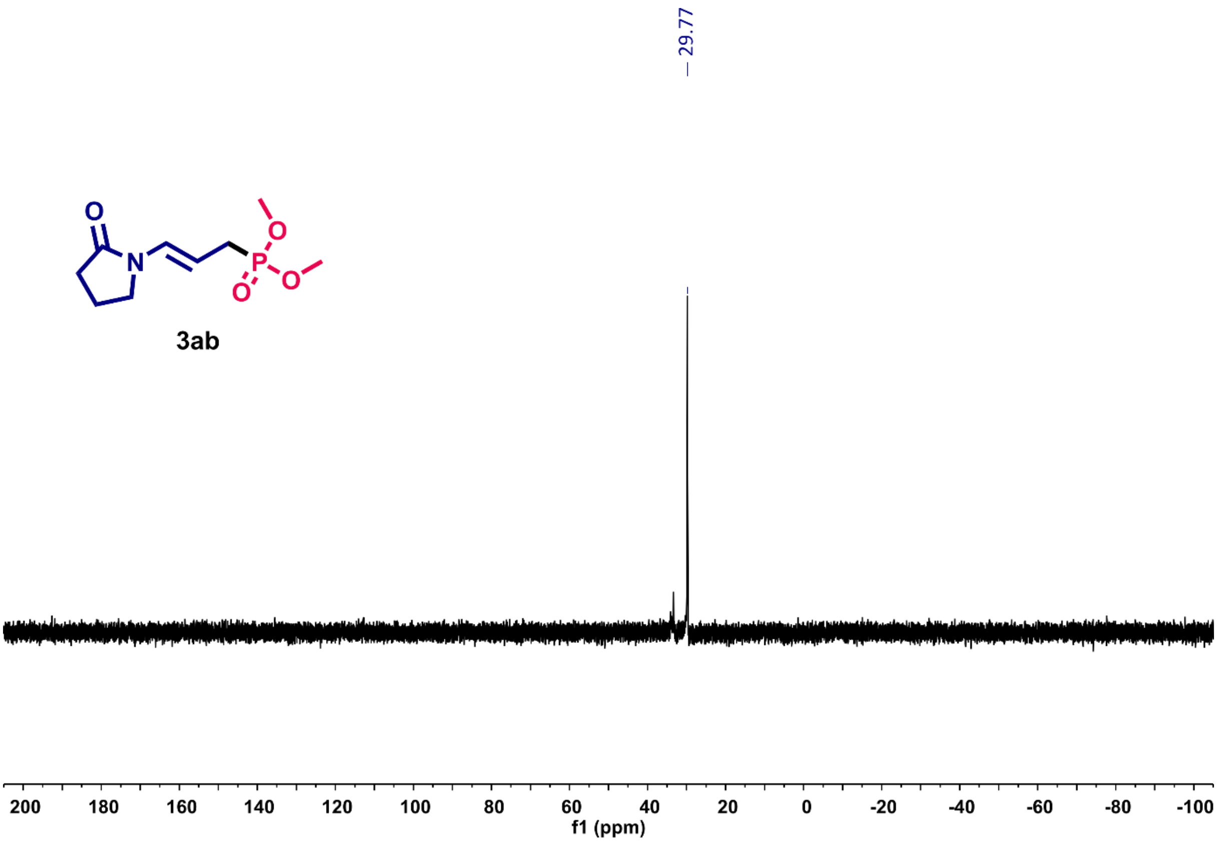

Figure S8. ^{31}P {^{1}H} NMR spectrum (162 MHz, $CDCl_3$) of *dimethyl (E)-(3-(2-oxopyrrolidin-1-yl)allyl)phosphonate* **3ab**.

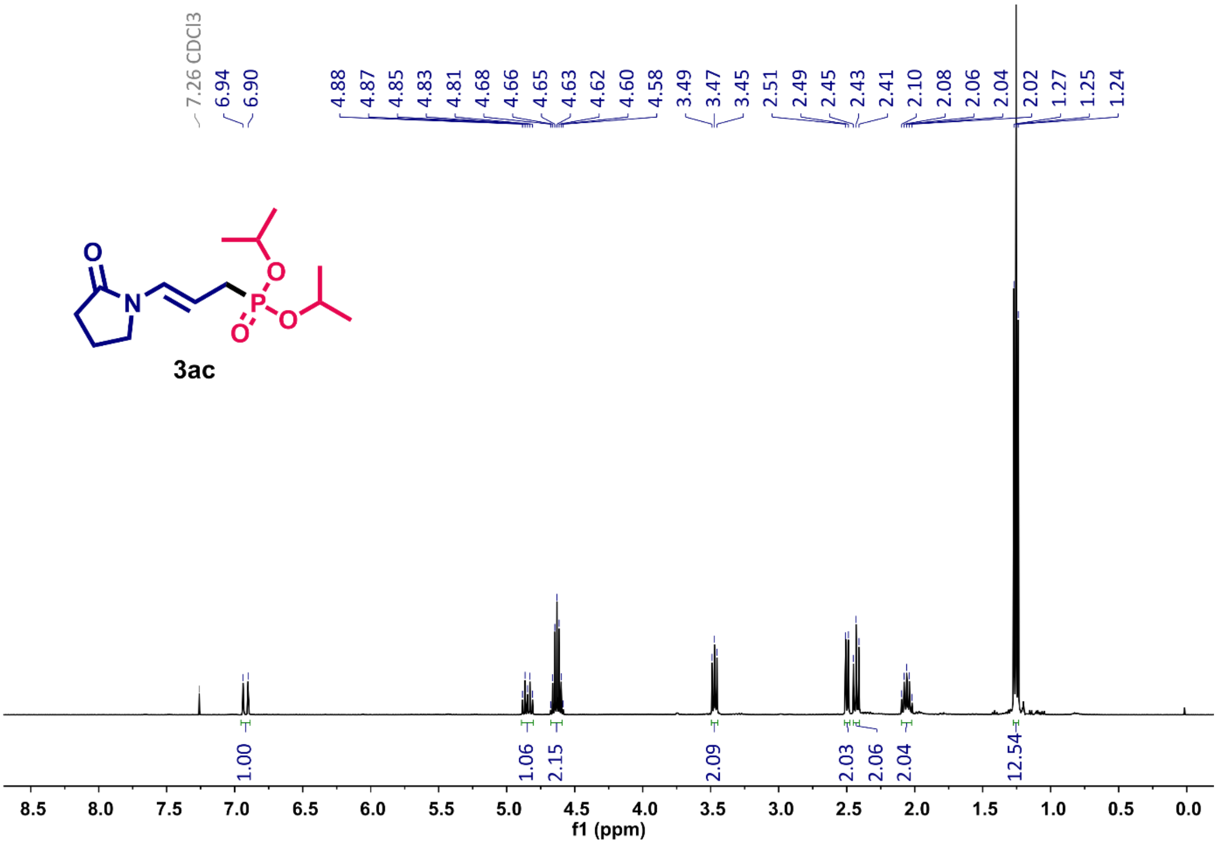

Figure S9. ^{1}H {^{31}P} NMR spectrum (400 MHz, $CDCl_3$) of *diisopropyl (E)-(3-(2-oxopyrrolidin-1-yl)allyl)phosphonate* **3ac**.

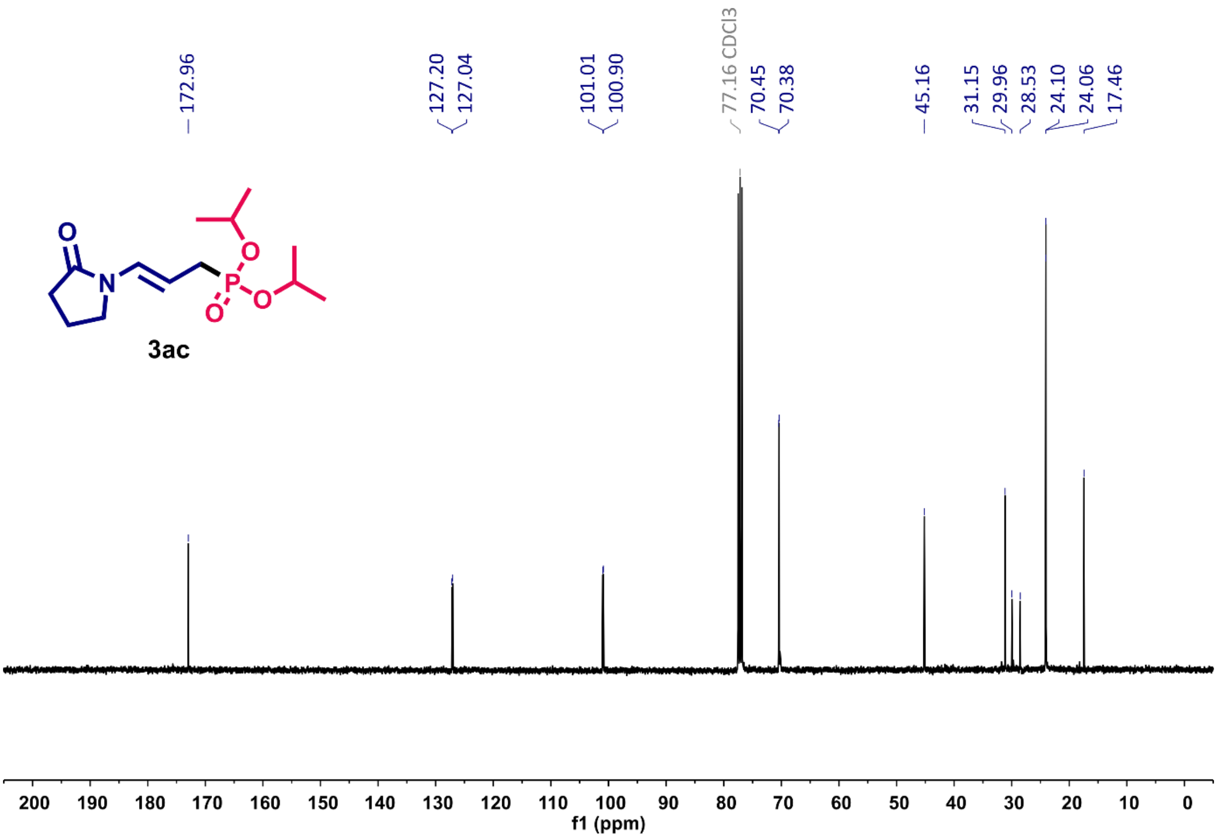

Figure S10. ^{13}C {^1H} NMR spectrum (101 MHz, $CDCl_3$) of *diisopropyl (E)-(3-(2-oxopyrrolidin-1-yl)allyl)phosphonate* **3ac**.

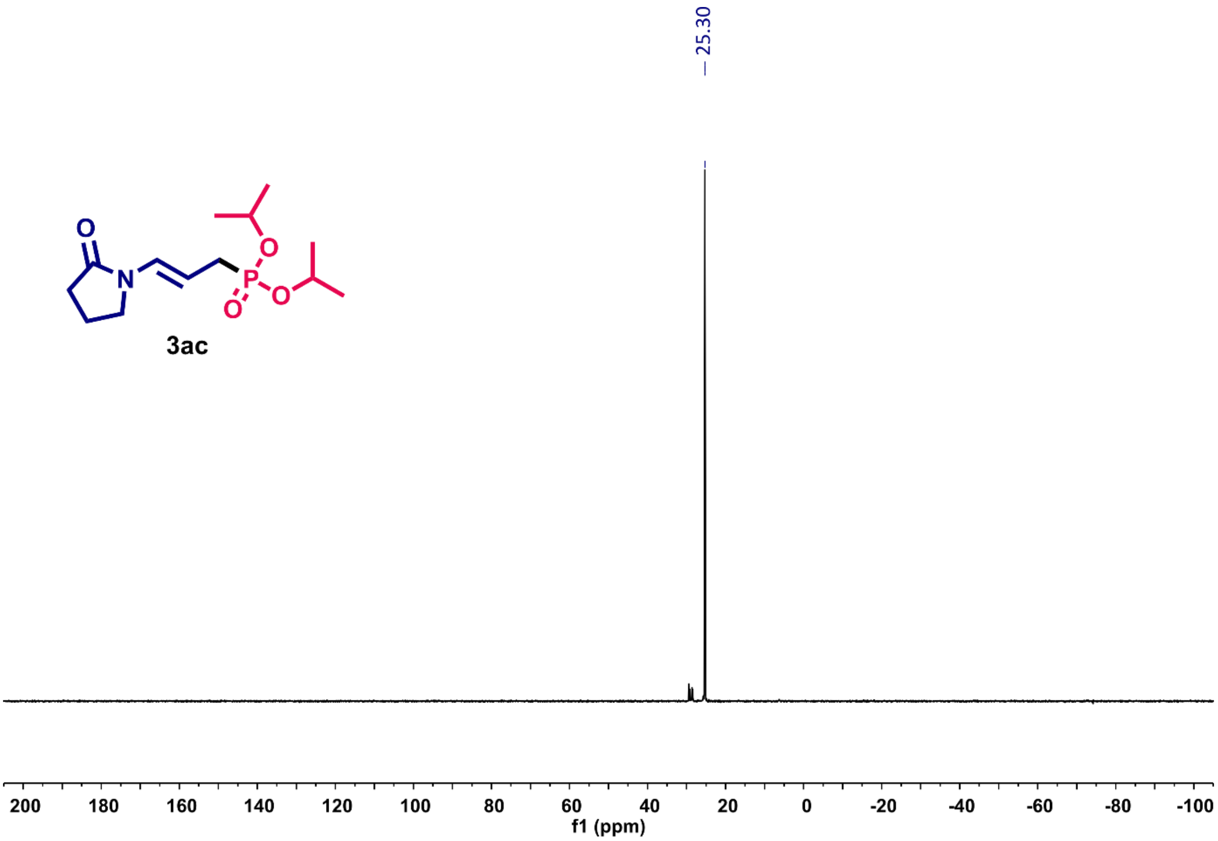

Figure S11. ^{31}P {^1H} NMR spectrum (162 MHz, $CDCl_3$) of *diisopropyl (E)-(3-(2-oxopyrrolidin-1-yl)allyl)phosphonate* **3ac**.

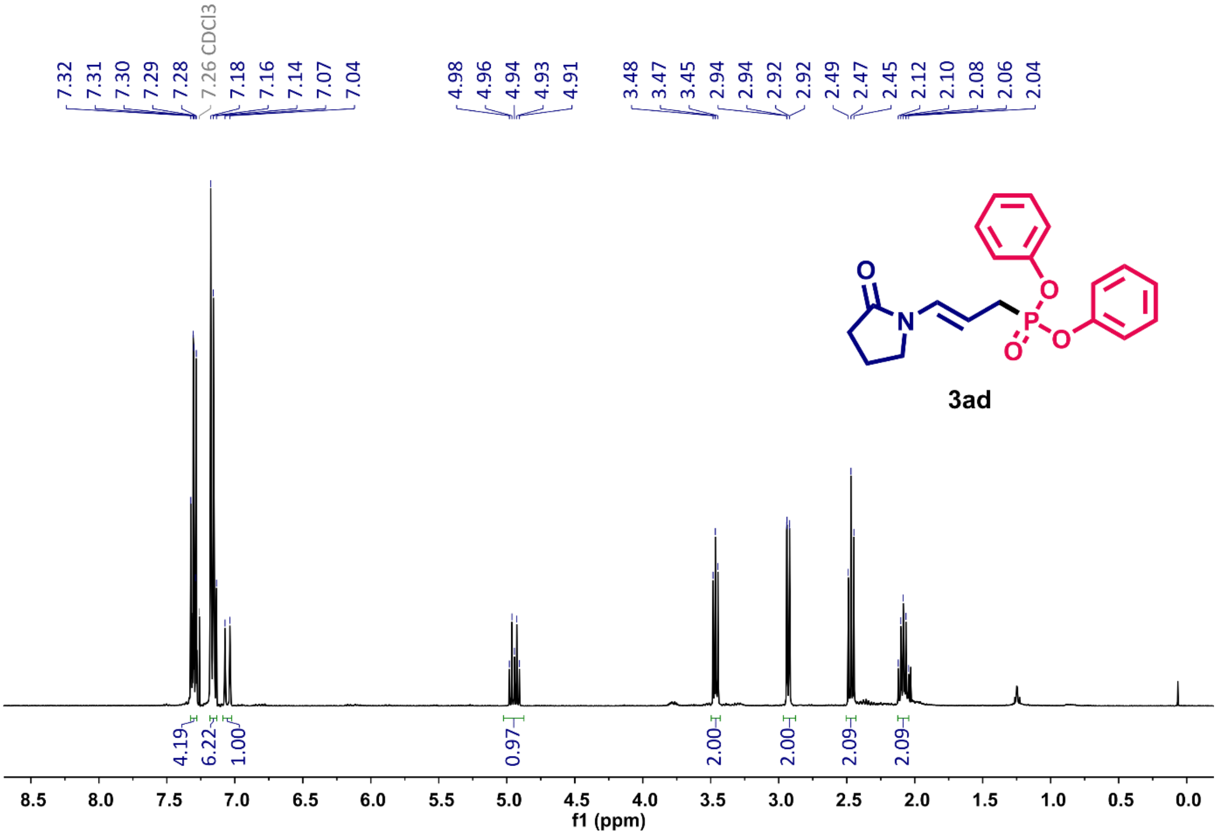

Figure S12. $^{1}H\ \{^{31}P\}$ NMR spectrum (400 MHz, $CDCl_3$) of *diphenyl (E)-(3-(2-oxopyrrolidin-1-yl)allyl)phosphonate* **3ad**.

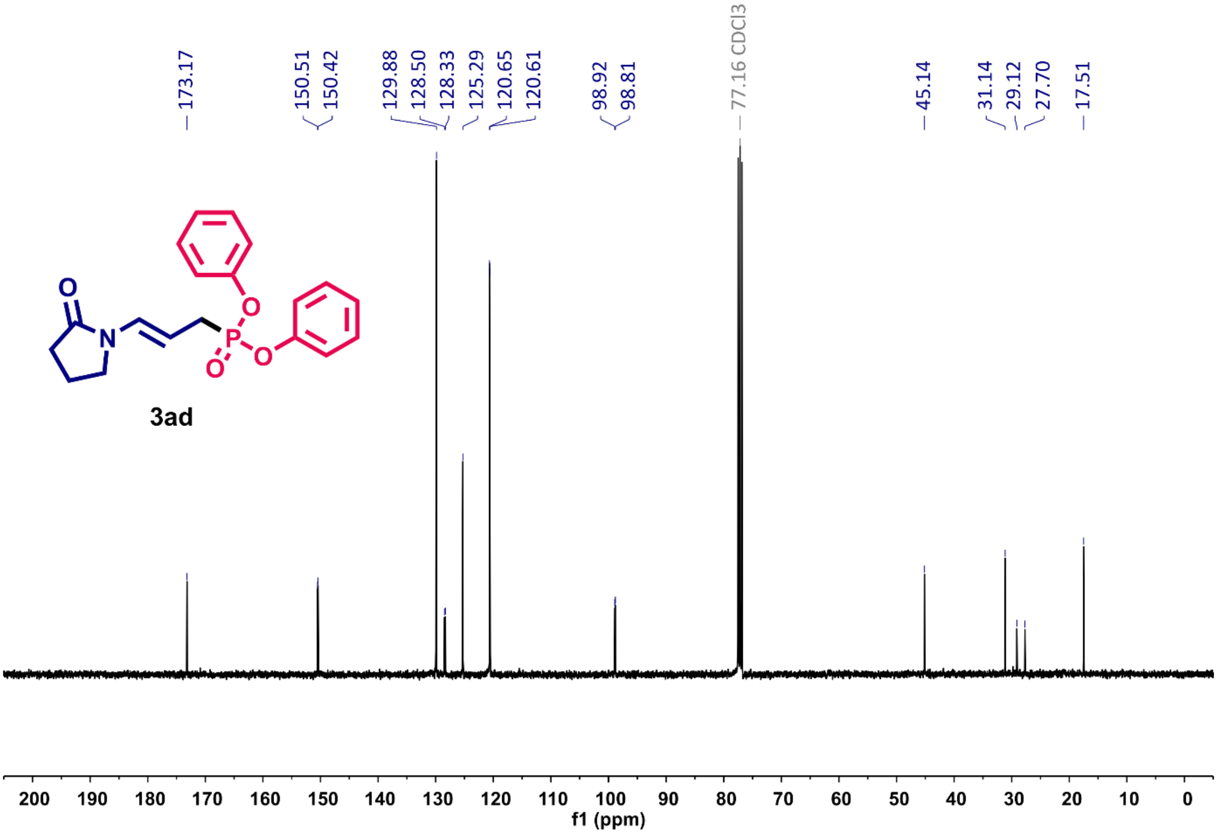

Figure S13. $^{13}C\ \{^{1}H\}$ NMR spectrum (101 MHz, $CDCl_3$) of *diphenyl (E)-(3-(2-oxopyrrolidin-1-yl)allyl)phosphonate* **3ad**.

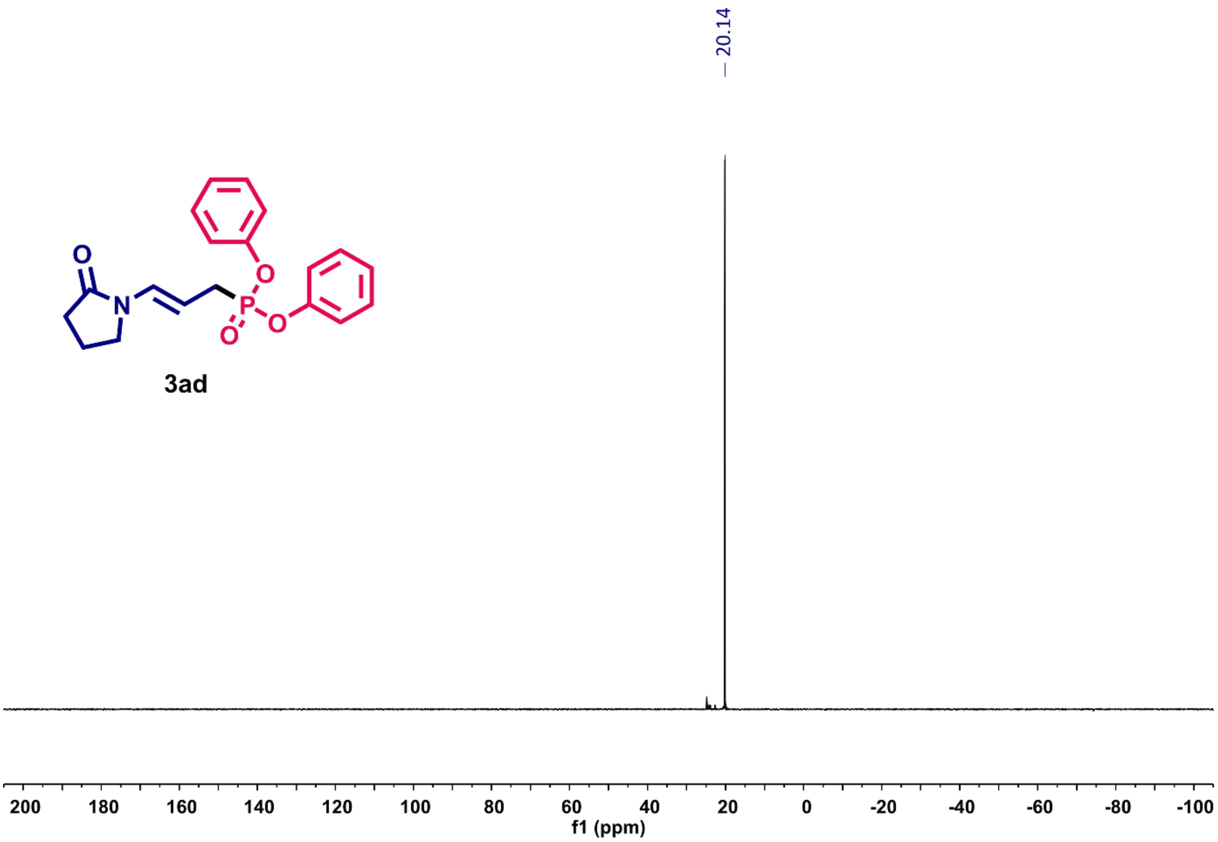

Figure S14. $^{31}P\ \{^1H\}$ NMR spectrum (162 MHz, $CDCl_3$) of *diphenyl (E)-(3-(2-oxopyrrolidin-1-yl)allyl)phosphonate* **3ad**.

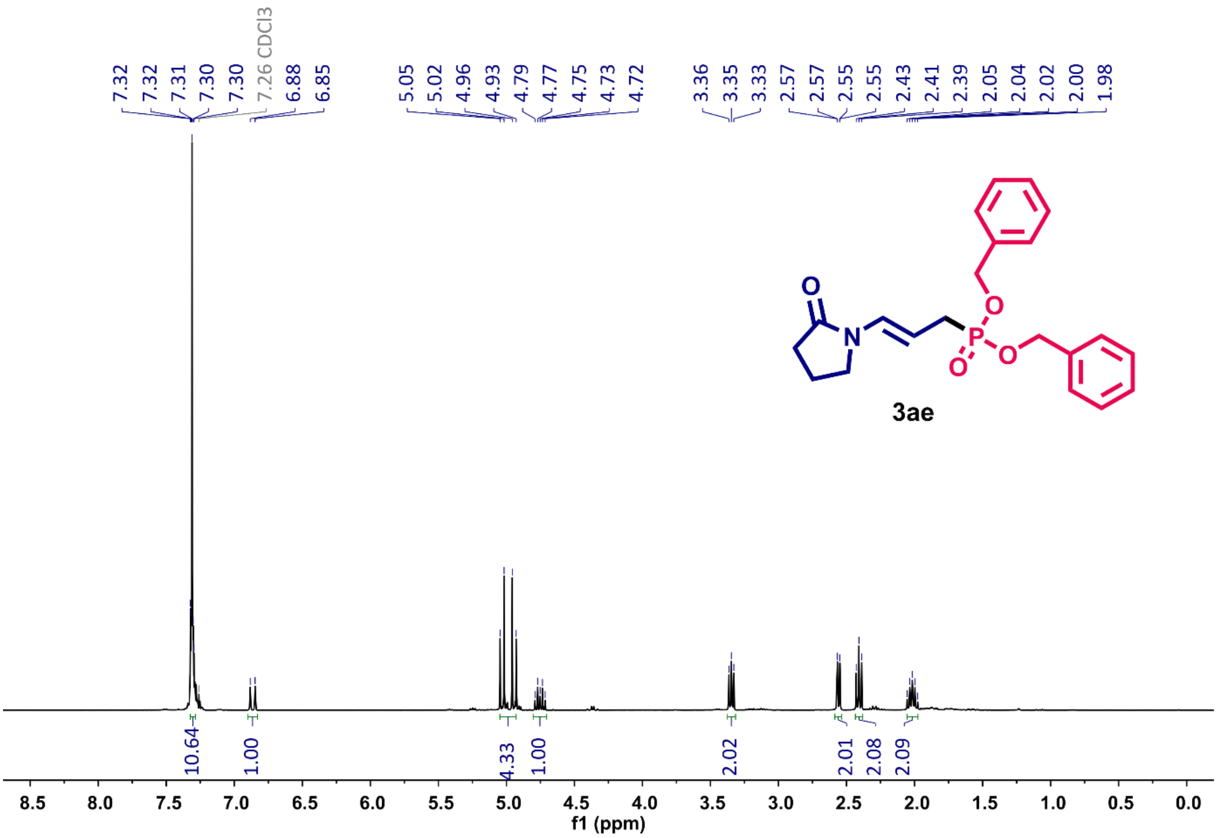

Figure S15. $^1H\ \{^{31}P\}$ NMR spectrum (400 MHz, $CDCl_3$) of *dibenzyl (E)-(3-(2-oxopyrrolidin-1-yl)allyl)phosphonate* **3ae**.

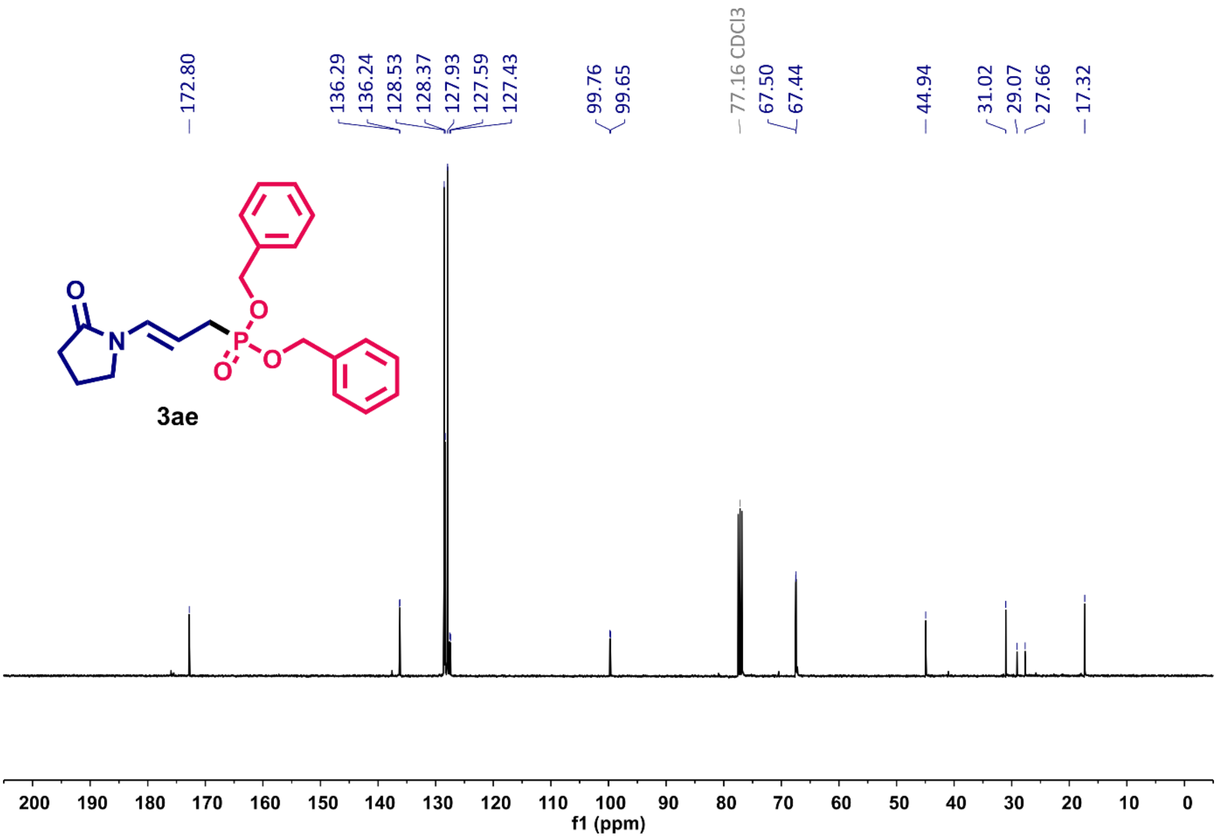

Figure S16. ^{13}C {^{1}H} NMR spectrum (101 MHz, $CDCl_3$) of *dibenzyl (E)-(3-(2-oxopyrrolidin-1-yl)allyl)phosphonate* **3ae**.

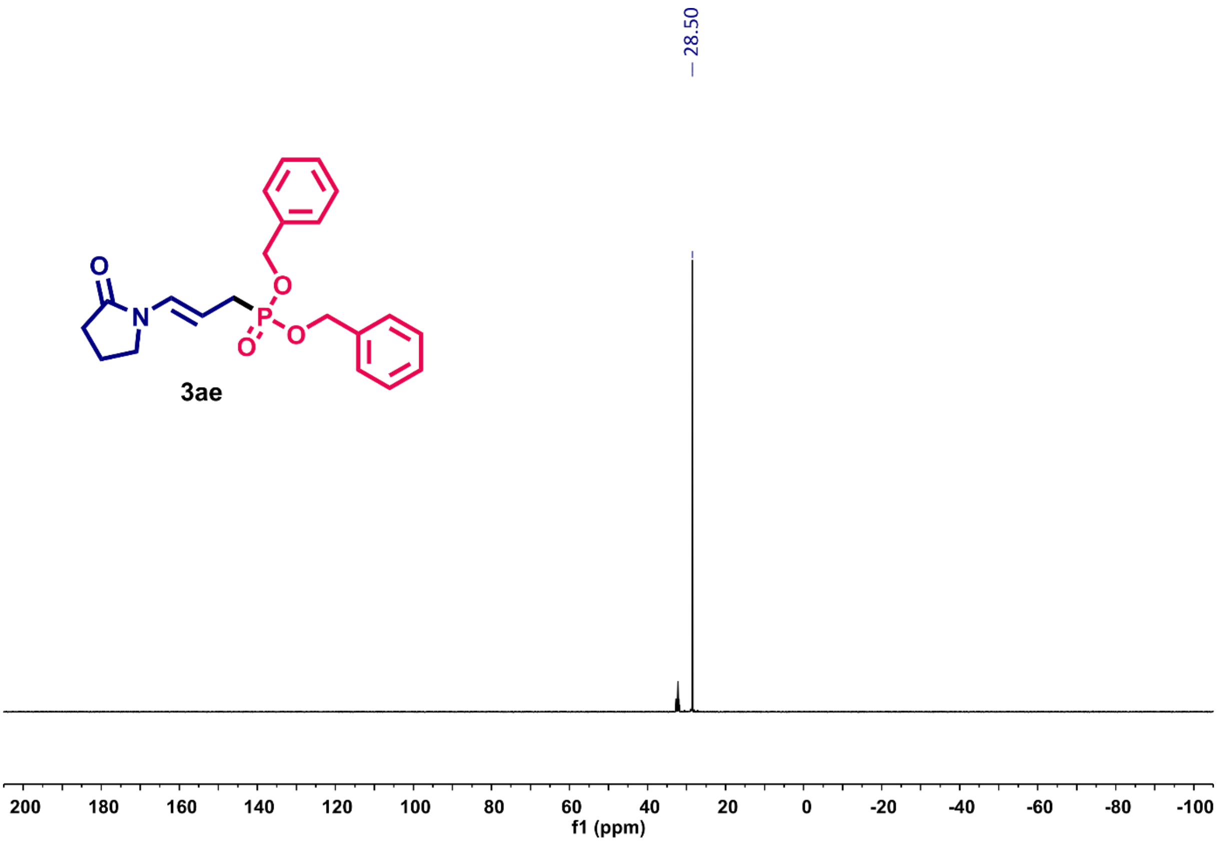

Figure S17. ^{31}P {^{1}H} NMR spectrum (162 MHz, $CDCl_3$) of *dibenzyl (E)-(3-(2-oxopyrrolidin-1-yl)allyl)phosphonate* **3ae**.

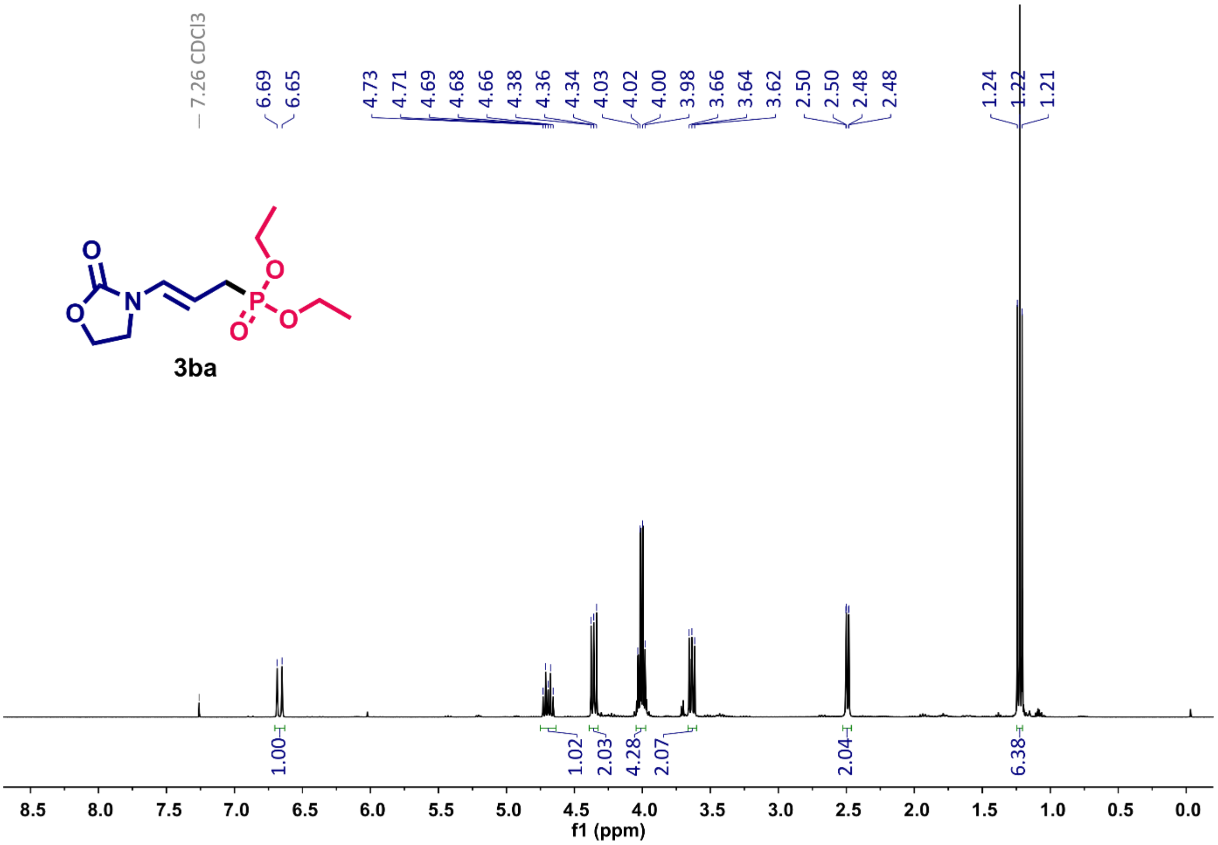

Figure S18. ^{1}H $\{^{31}P\}$ NMR spectrum (400 MHz, $CDCl_3$) of *diethyl (E)-(3-(2-oxooxazolidin-3-yl)allyl)phosphonate* **3ba**.

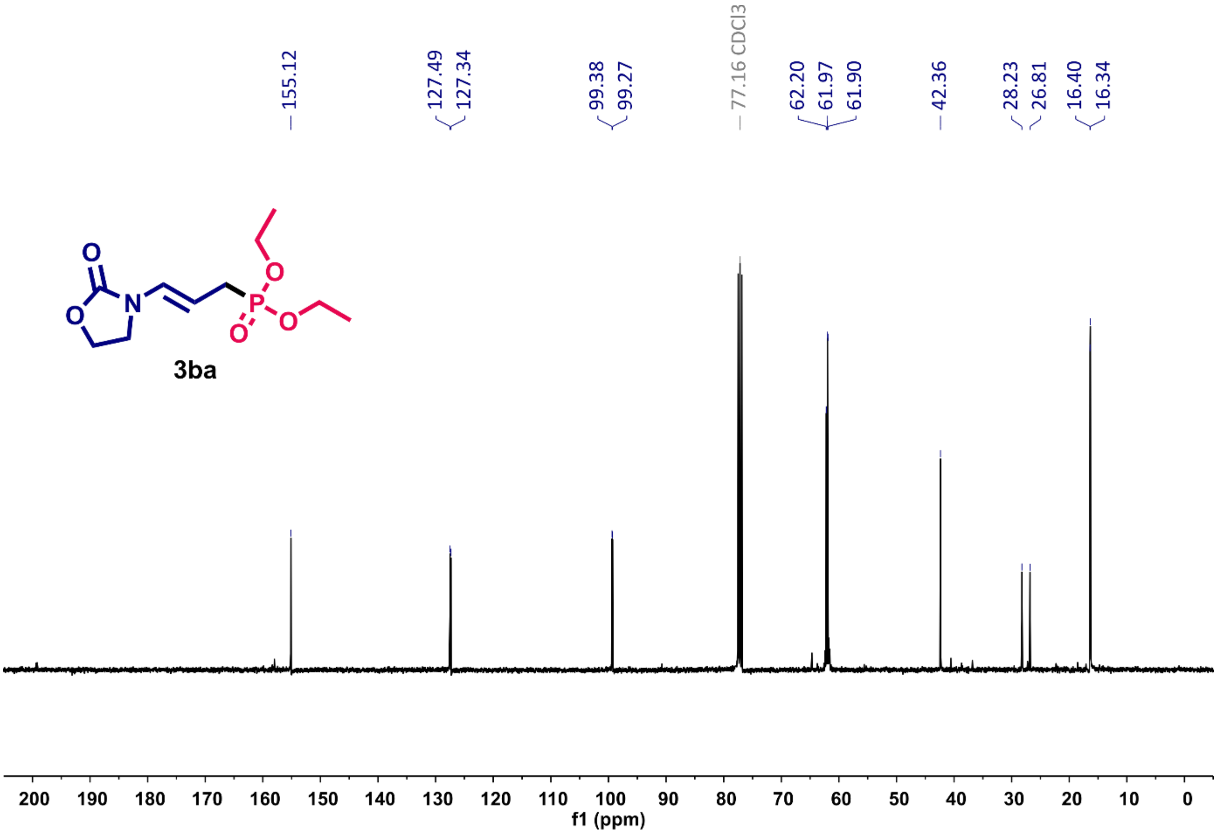

Figure S19. ^{13}C $\{^{1}H\}$ NMR spectrum (101 MHz, $CDCl_3$) of *diethyl (E)-(3-(2-oxooxazolidin-3-yl)allyl)phosphonate* **3ba**.

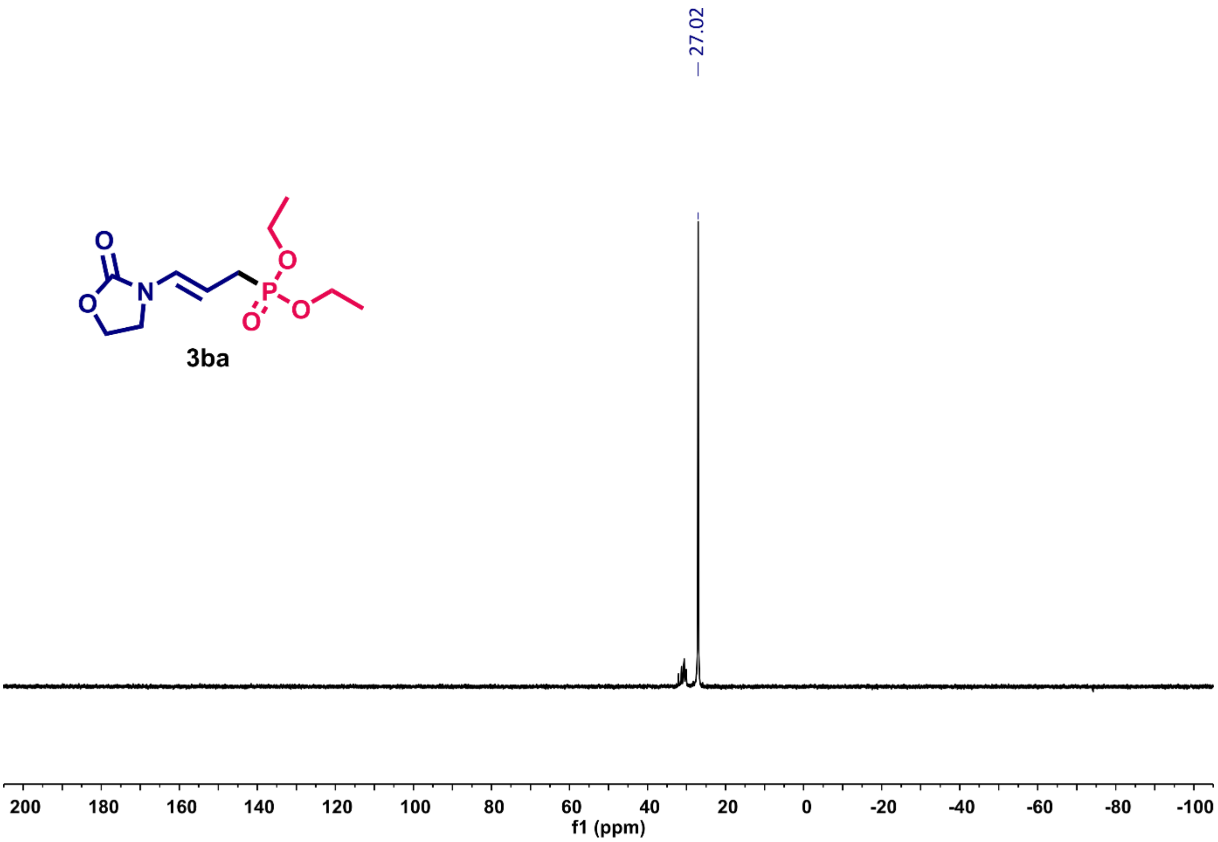

Figure S20. ^{31}P {^{1}H} NMR spectrum (162 MHz, $CDCl_3$) of *diethyl (E)-(3-(2-oxooxazolidin-3-yl)allyl)phosphonate* **3ba**.

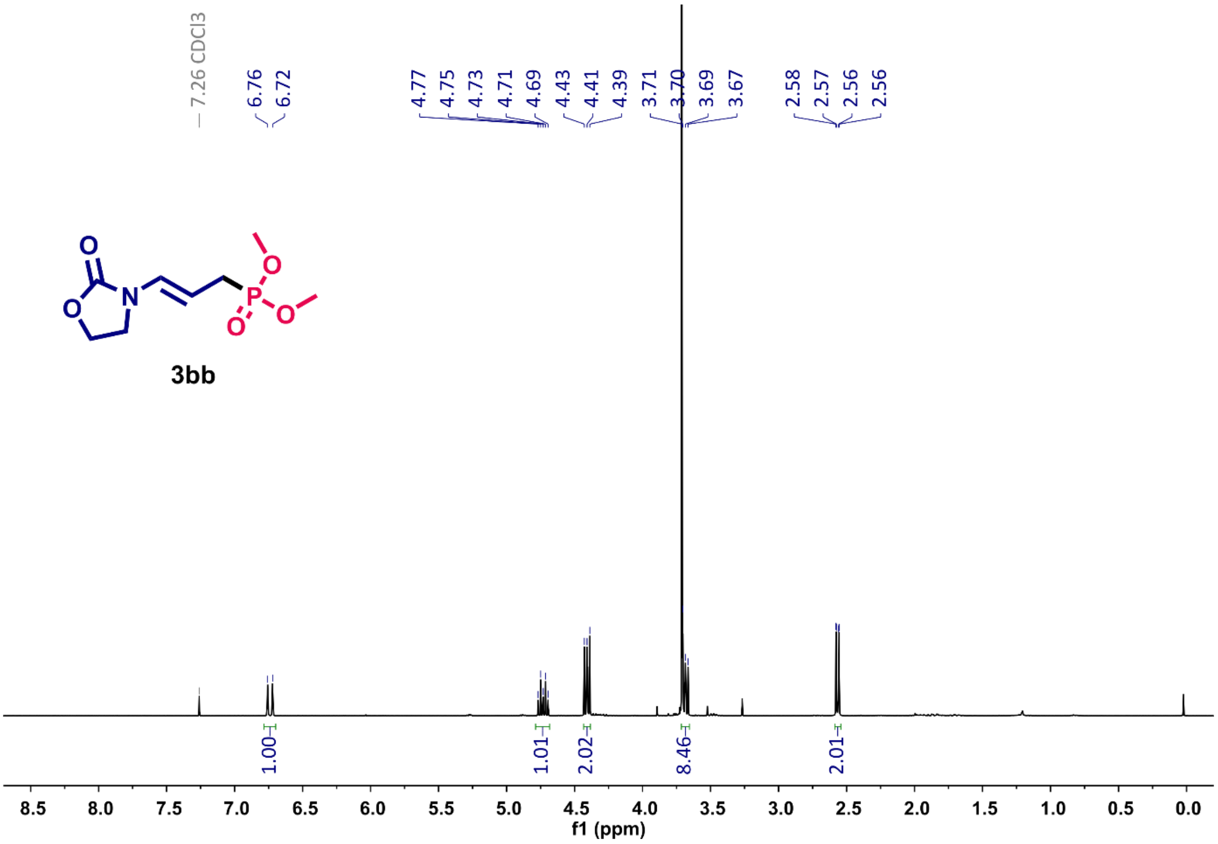

Figure S21. ^{1}H {^{31}P} NMR spectrum (400 MHz, $CDCl_3$) of *dimethyl (E)-(3-(2-oxooxazolidin-3-yl)allyl)phosphonate* **3bb**.

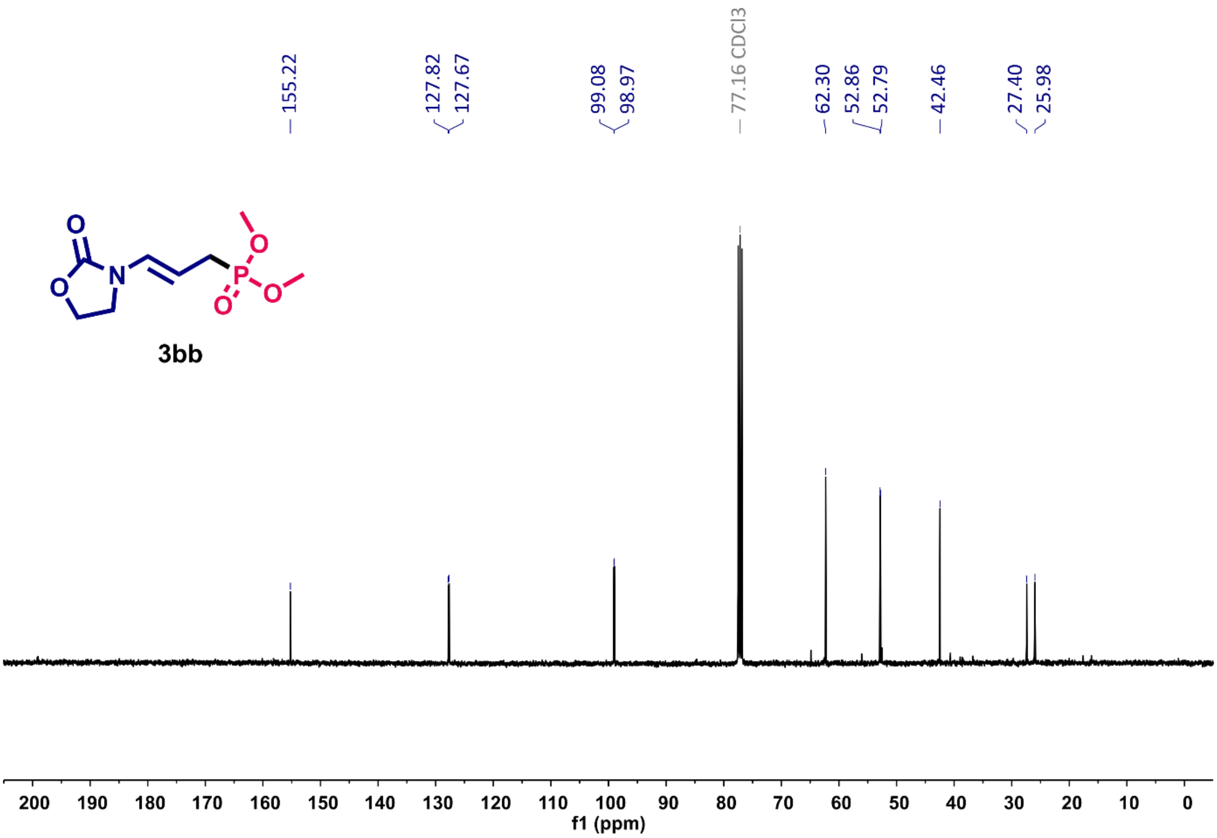

Figure S22. ^{13}C {^{1}H} NMR spectrum (101 MHz, $CDCl_3$) of *dimethyl (E)-(3-(2-oxooxazolidin-3-yl)allyl)phosphonate* **3bb**.

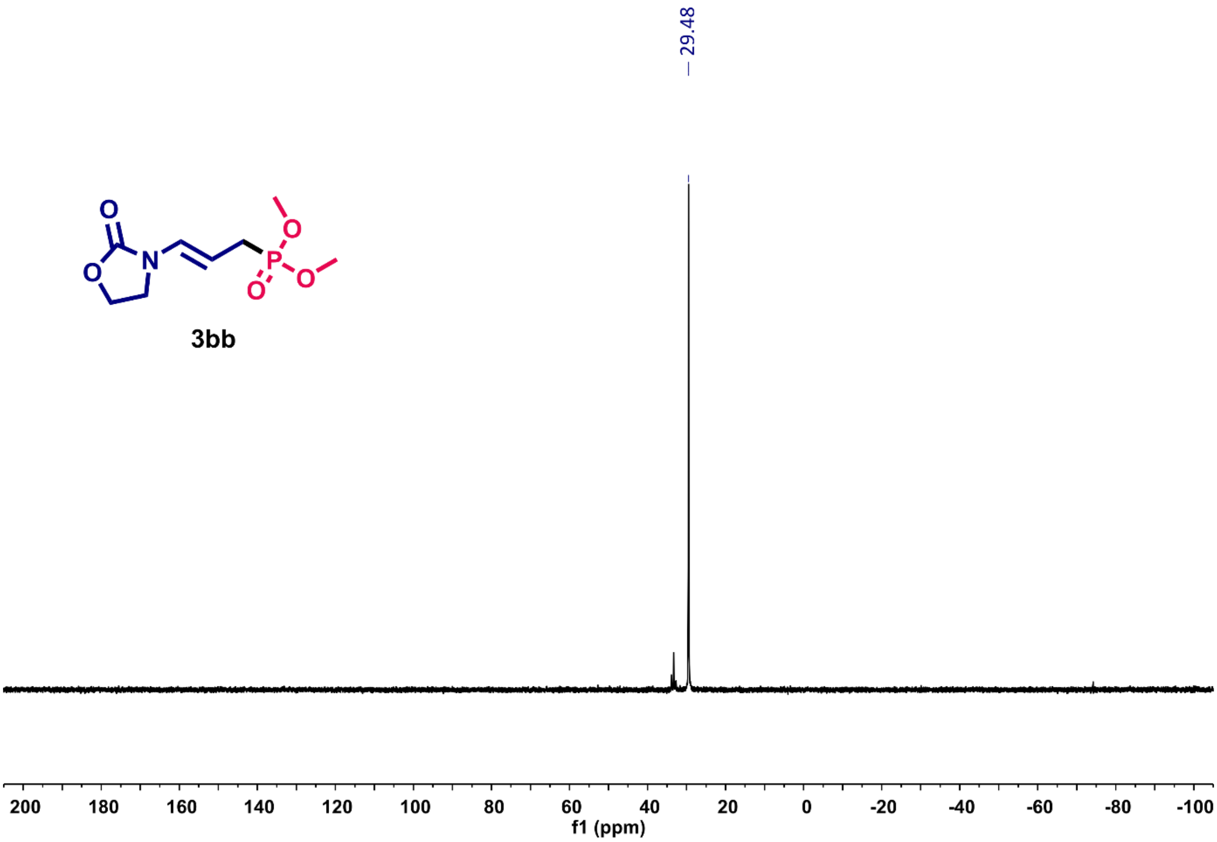

Figure S23. ^{31}P {^{1}H} NMR spectrum (162 MHz, $CDCl_3$) of *dimethyl (E)-(3-(2-oxooxazolidin-3-yl)allyl)phosphonate* **3bb**.

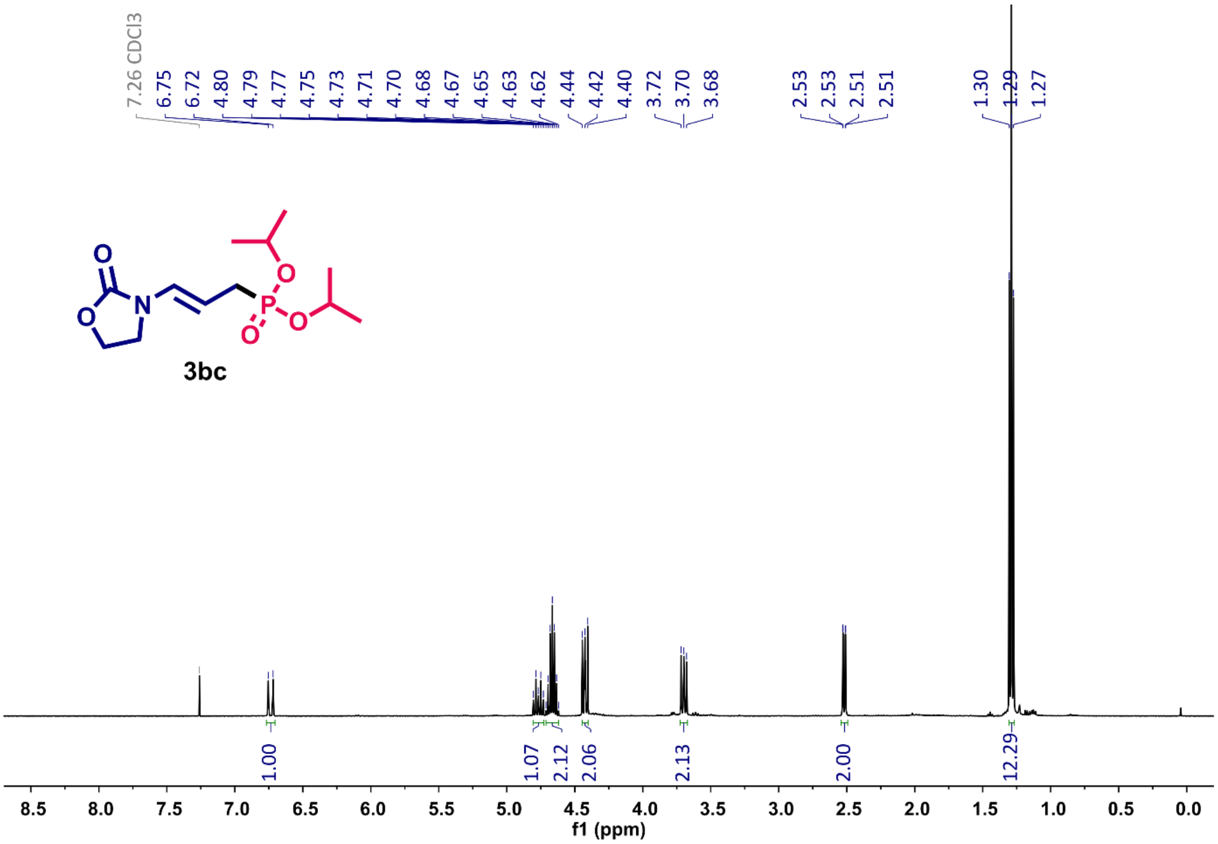

Figure S24. ^{1}H {^{31}P} NMR spectrum (400 MHz, $CDCl_3$) of *diisopropyl (E)-(3-(2-oxooxazolidin-3-yl)allyl)phosphonate* **3bc**.

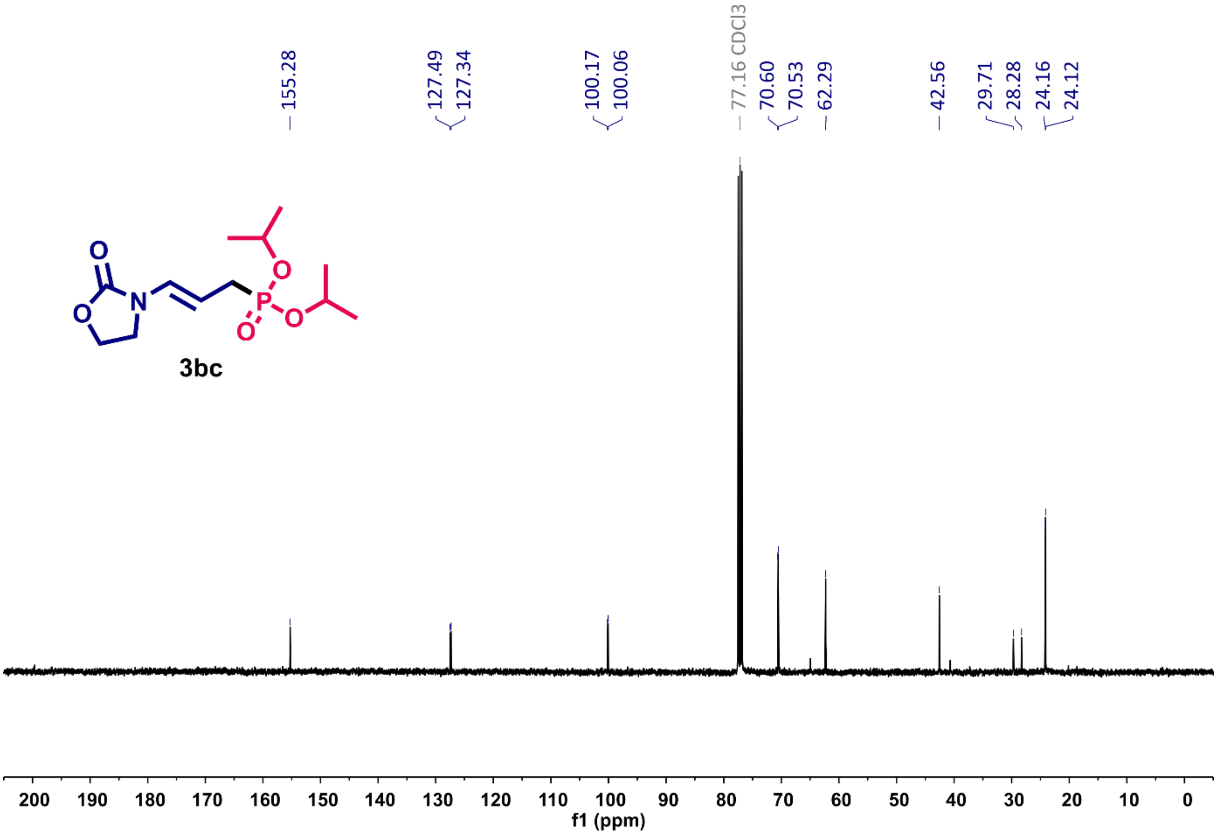

Figure S25. ^{13}C {^{1}H} NMR spectrum (101 MHz, $CDCl_3$) of *diisopropyl (E)-(3-(2-oxooxazolidin-3-yl)allyl)phosphonate* **3bc**.

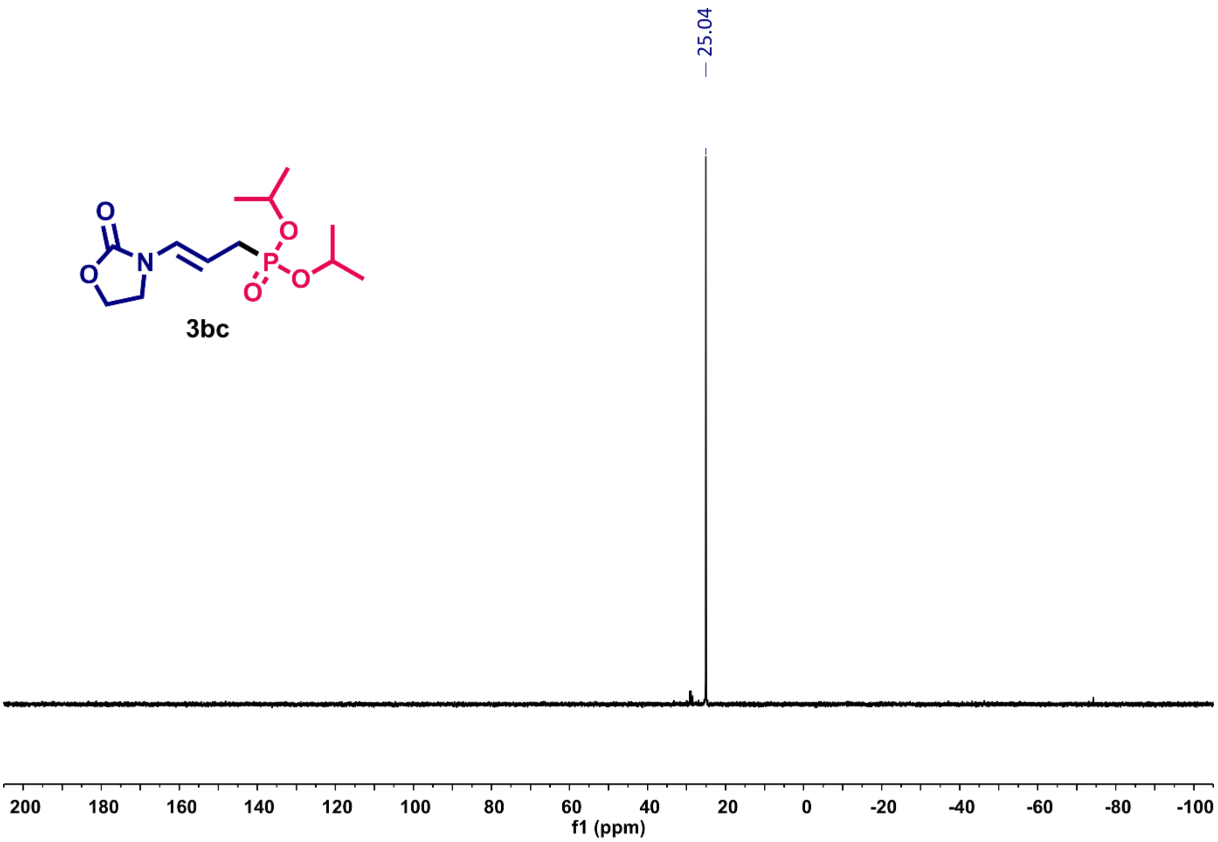

Figure S26. ^{31}P {^{1}H} NMR spectrum (162 MHz, $CDCl_3$) of *diisopropyl (E)-(3-(2-oxooxazolidin-3-yl)allyl)phosphonate* **3bc**.

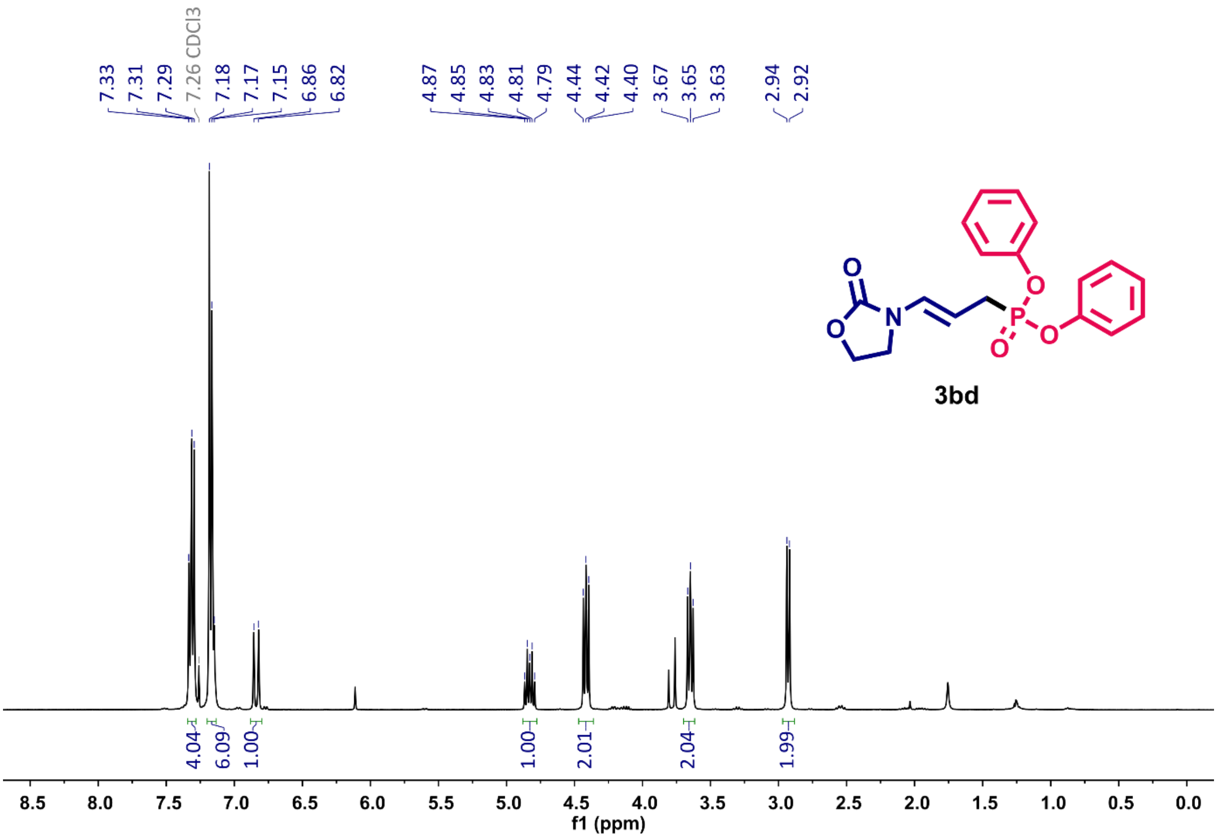

Figure S27. ^{1}H {^{31}P} NMR spectrum (400 MHz, $CDCl_3$) of *diphenyl (E)-(3-(2-oxooxazolidin-3-yl)allyl)phosphonate* **3bd**.

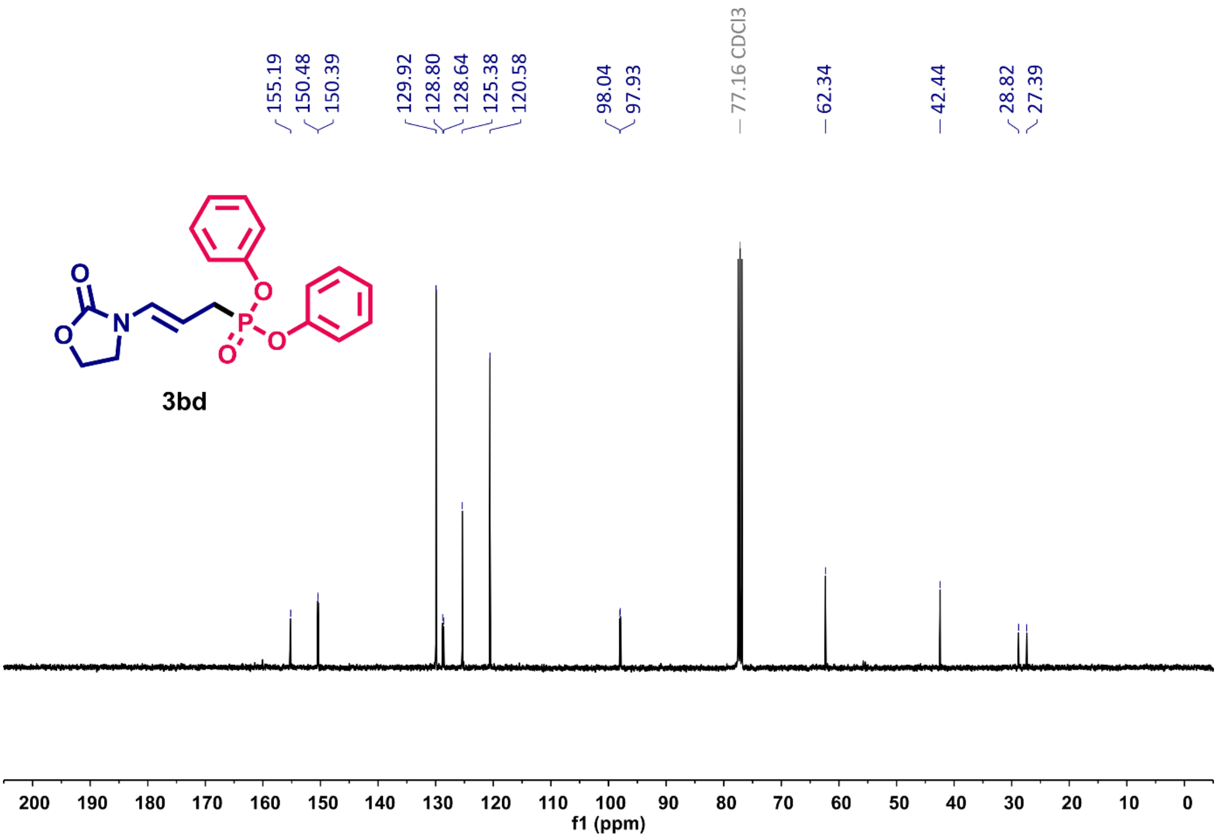

Figure S28. ^{13}C {^{1}H} NMR spectrum (101 MHz, $CDCl_3$) of *diphenyl (E)-(3-(2-oxooxazolidin-3-yl)allyl)phosphonate* **3bd**.

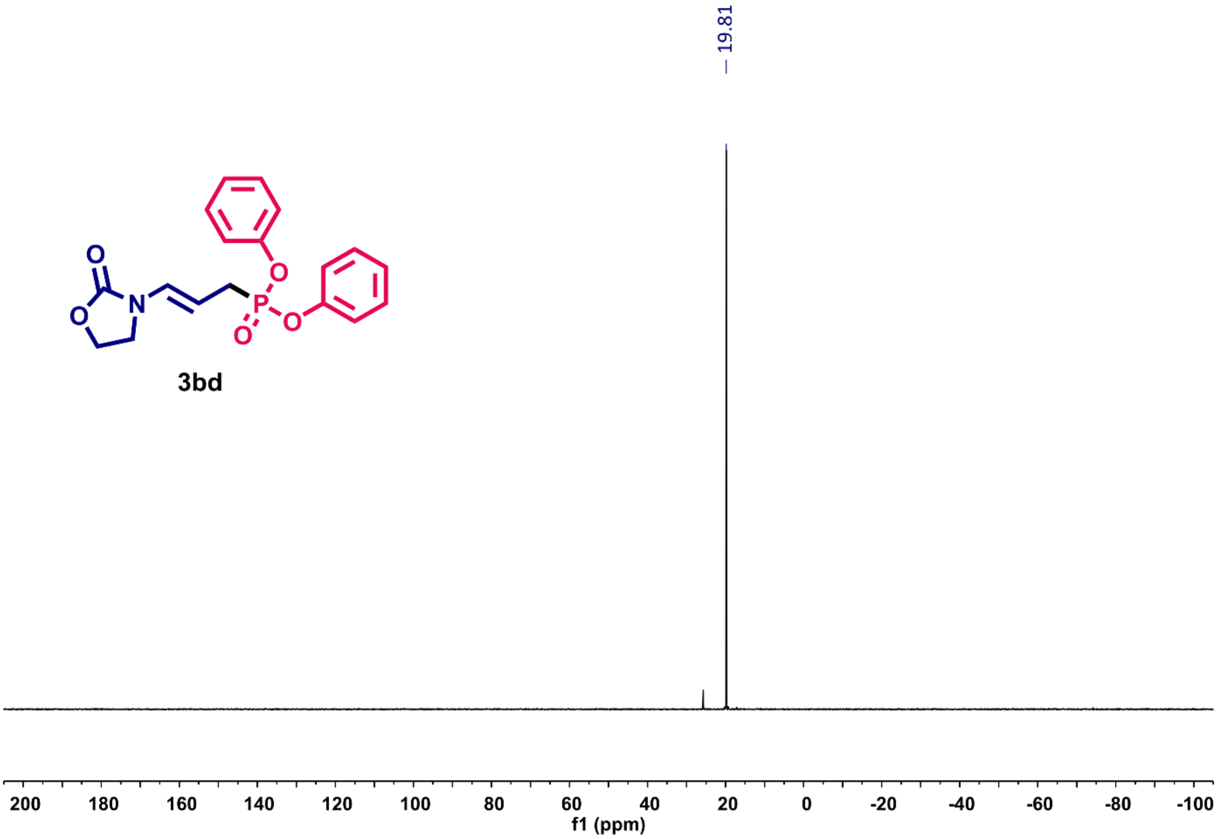

Figure S29. ^{31}P {^{1}H} NMR spectrum (162 MHz, $CDCl_3$) of *diphenyl (E)-(3-(2-oxooxazolidin-3-yl)allyl)phosphonate* **3bd**.

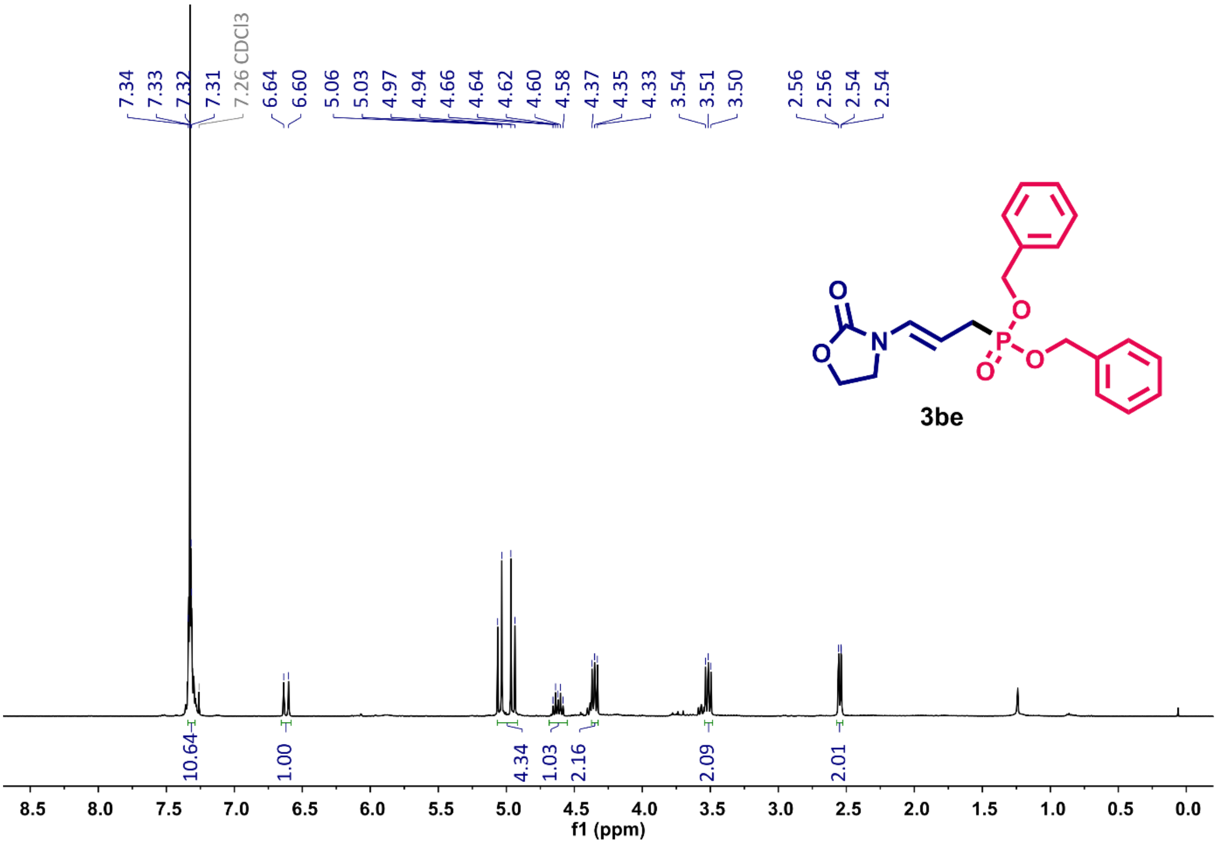

Figure S30. $^{1}H\ \{^{31}P\}$ NMR spectrum (400 MHz, $CDCl_3$) of *dibenzyl (E)-(3-(2-oxooxazolidin-3-yl)allyl)phosphonate* **3be**.

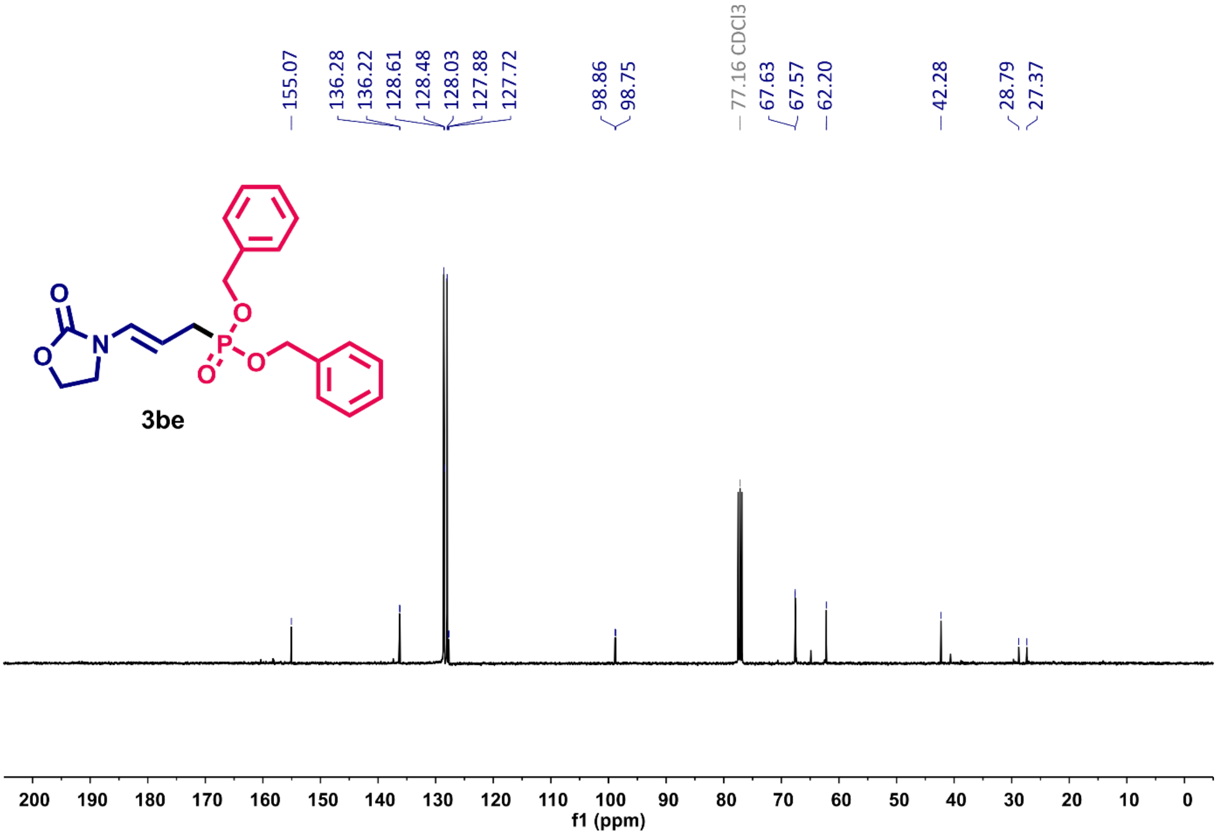

Figure S31. $^{13}C\ \{^{1}H\}$ NMR spectrum (101 MHz, $CDCl_3$) of *dibenzyl (E)-(3-(2-oxooxazolidin-3-yl)allyl)phosphonate* **3be**.

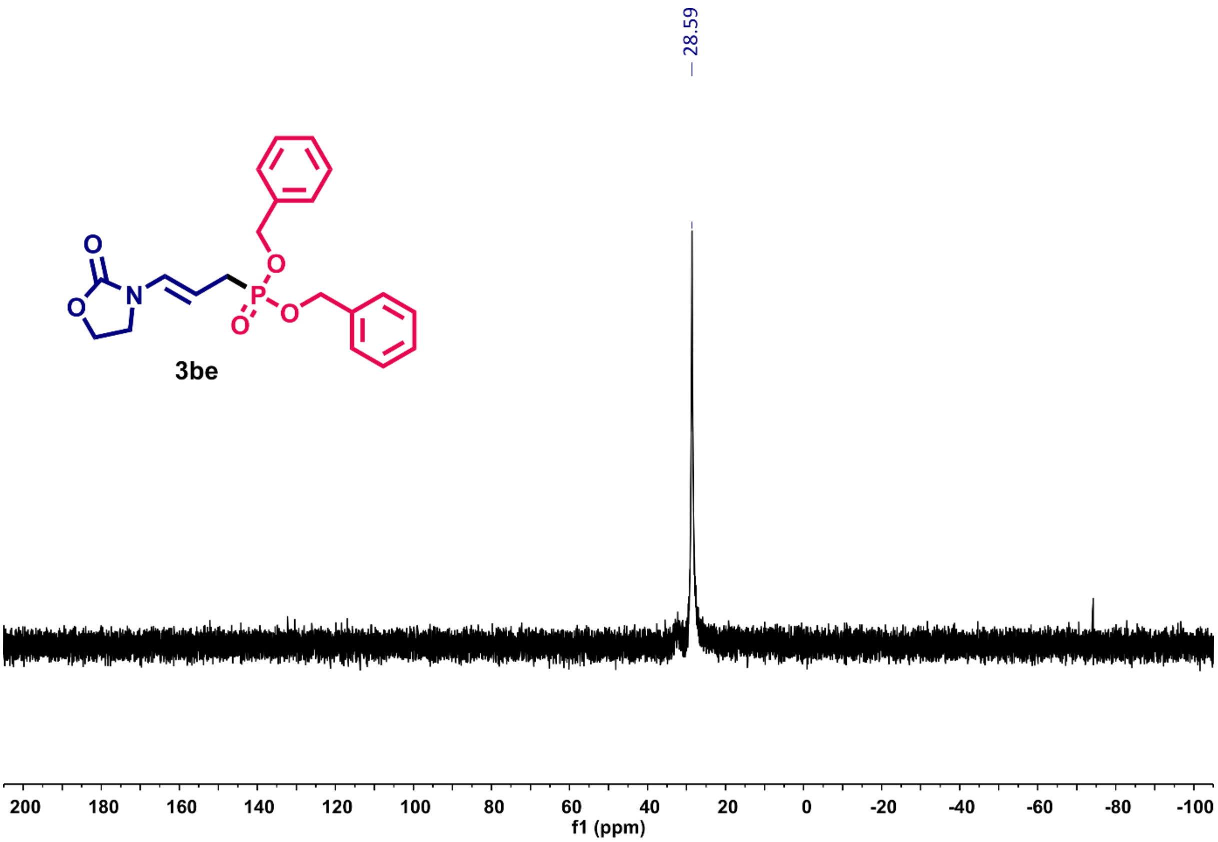

Figure S32. ^{31}P {^{1}H} NMR spectrum (162 MHz, $CDCl_3$) of *dibenzyl (E)-(3-(2-oxooxazolidin-3-yl)allyl)phosphonate* **3be**.

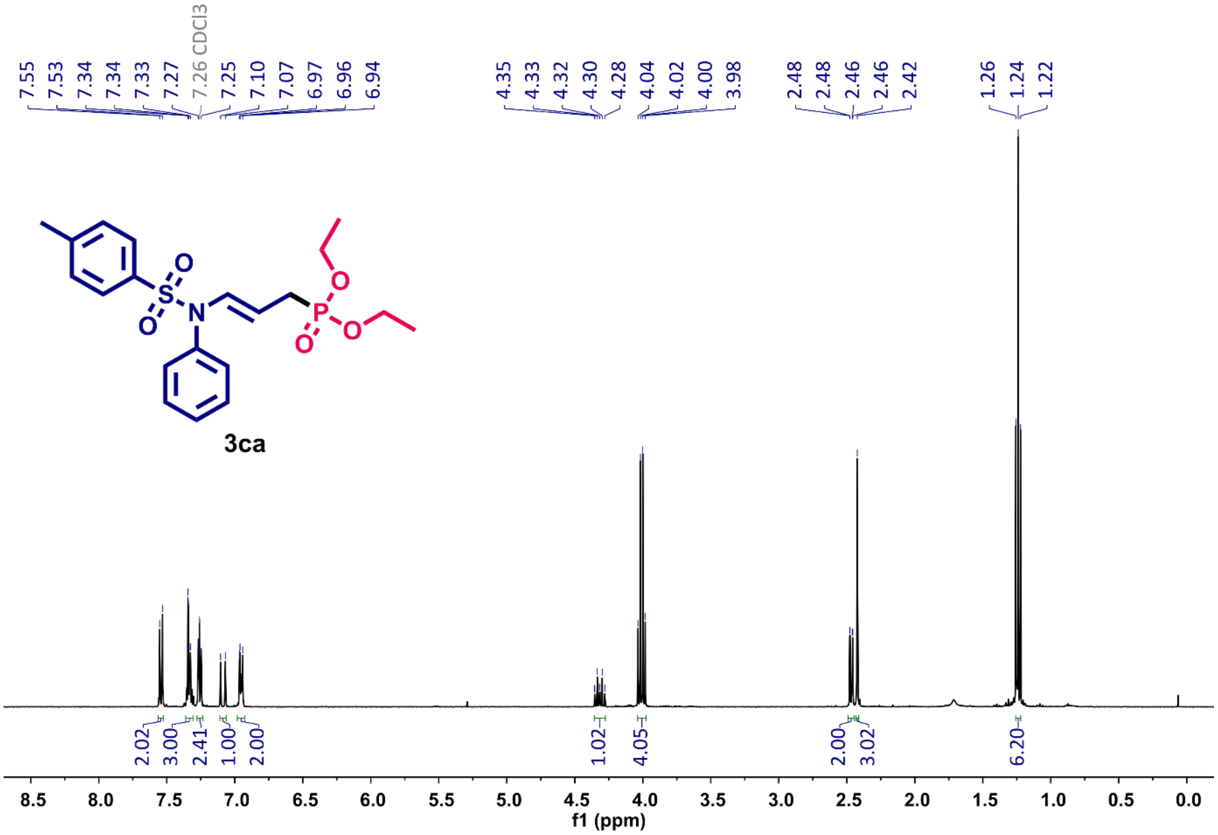

Figure S33. ^{1}H {^{31}P} NMR spectrum (400 MHz, $CDCl_3$) of *diethyl (E)-(3-((4-methyl-N-phenylphenyl)sulfonamido)allyl) phosphonate* **3ca**.

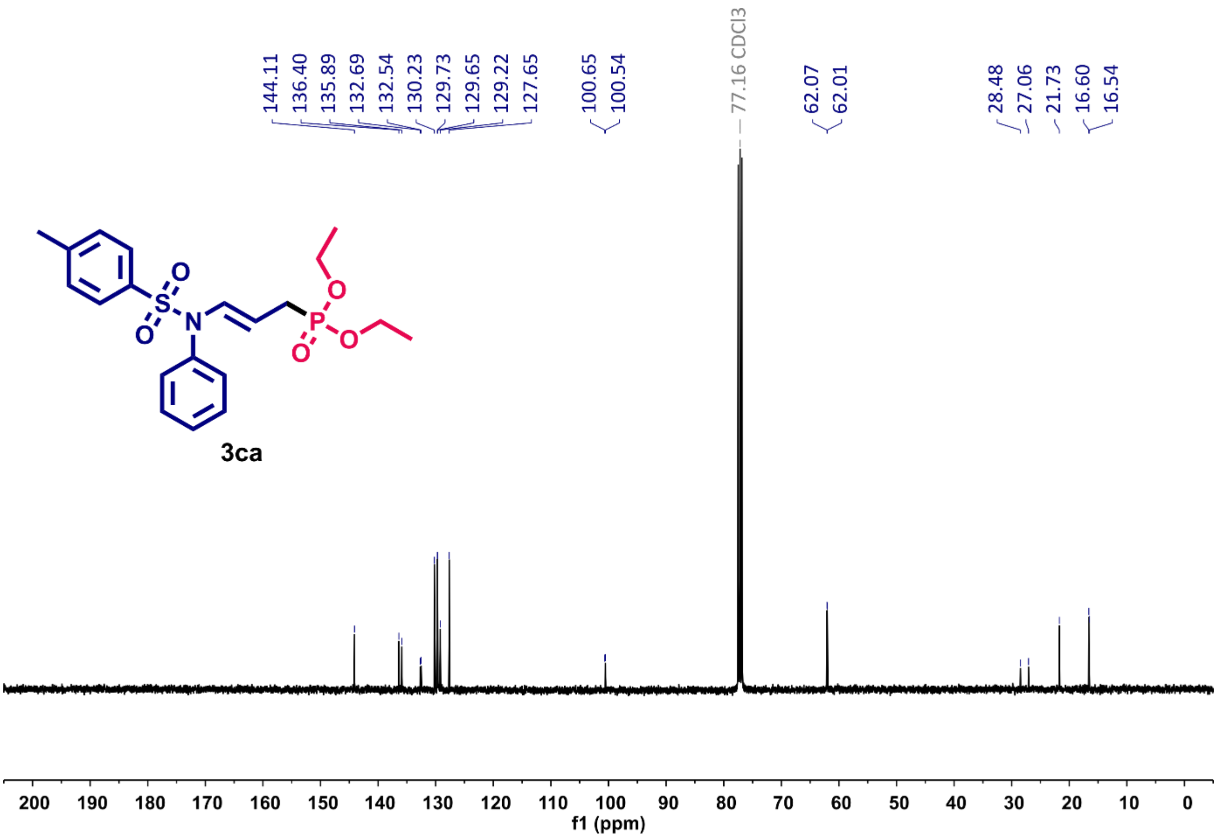

Figure S34. ^{13}C {^{1}H} NMR spectrum (101 MHz, $CDCl_3$) of *diethyl (E)-(3-((4-methyl-N-phenylphenyl)sulfonamido)allyl) phosphonate* **3ca**.

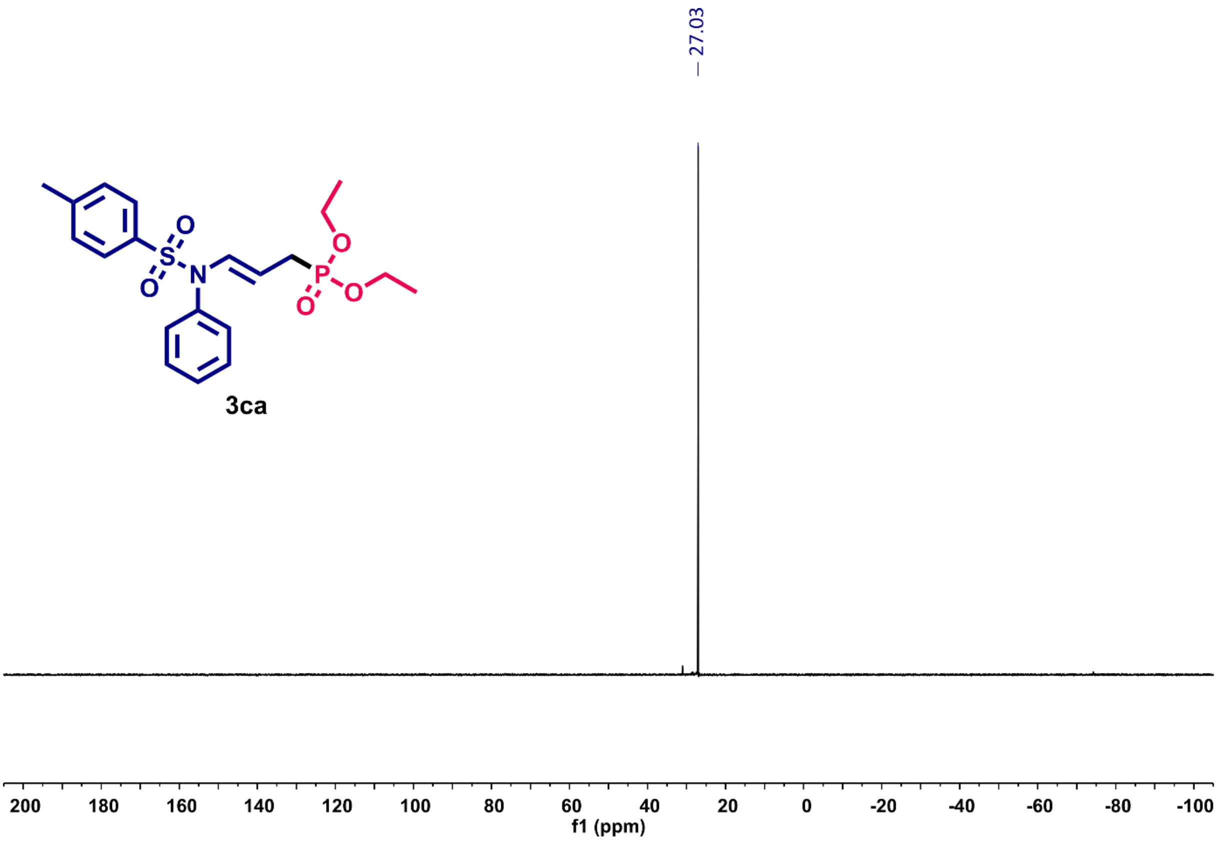

Figure S35. ^{31}P {^{1}H} NMR spectrum (162 MHz, $CDCl_3$) of *diethyl (E)-(3-((4-methyl-N-phenylphenyl)sulfonamido)allyl) phosphonate* **3ca**.

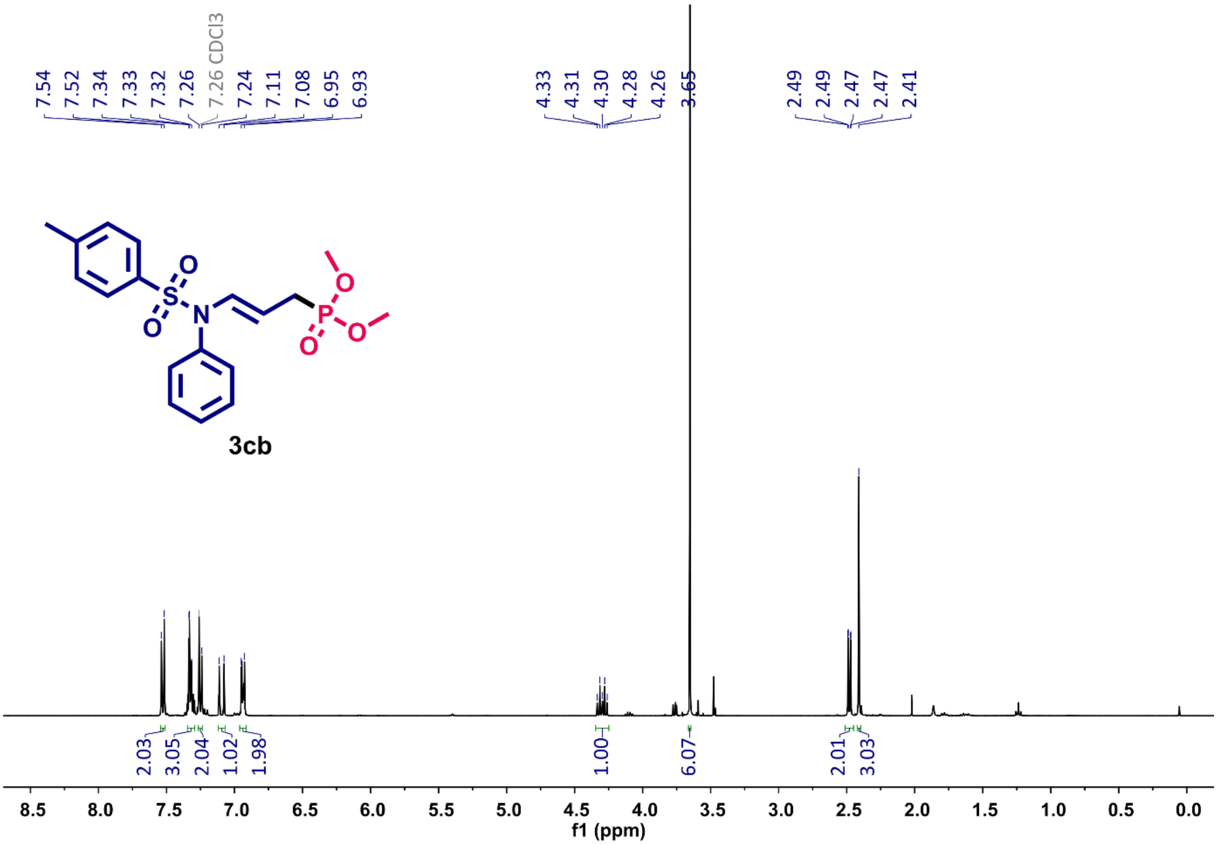

Figure S36. ^{1}H {^{31}P} NMR spectrum (400 MHz, $CDCl_3$) of *dimethyl (E)-(3-((4-methyl-N-phenylphenyl)sulfonamido)allyl) phosphonate* **3cb**.

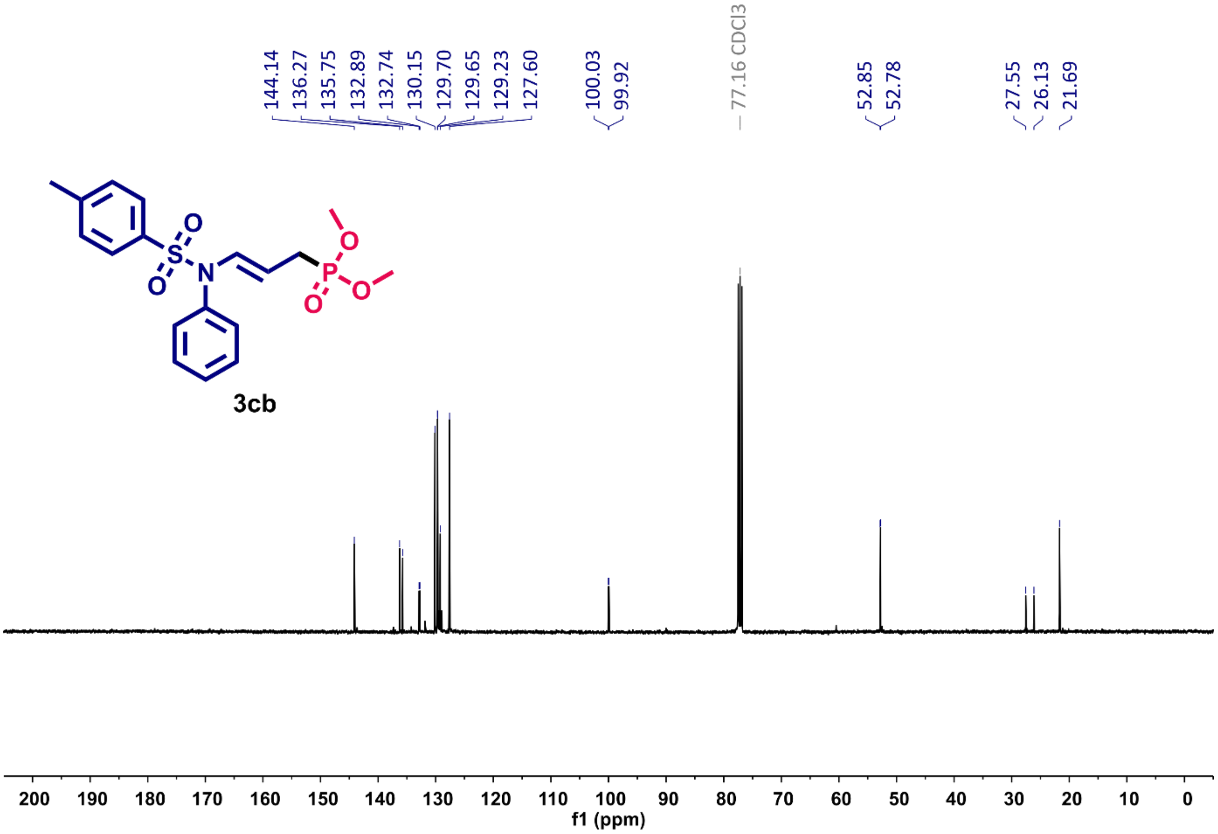

Figure S37. ^{13}C {^{1}H} NMR spectrum (101 MHz, $CDCl_3$) of *dimethyl (E)-(3-((4-methyl-N-phenylphenyl)sulfonamido)allyl) phosphonate* **3cb**.

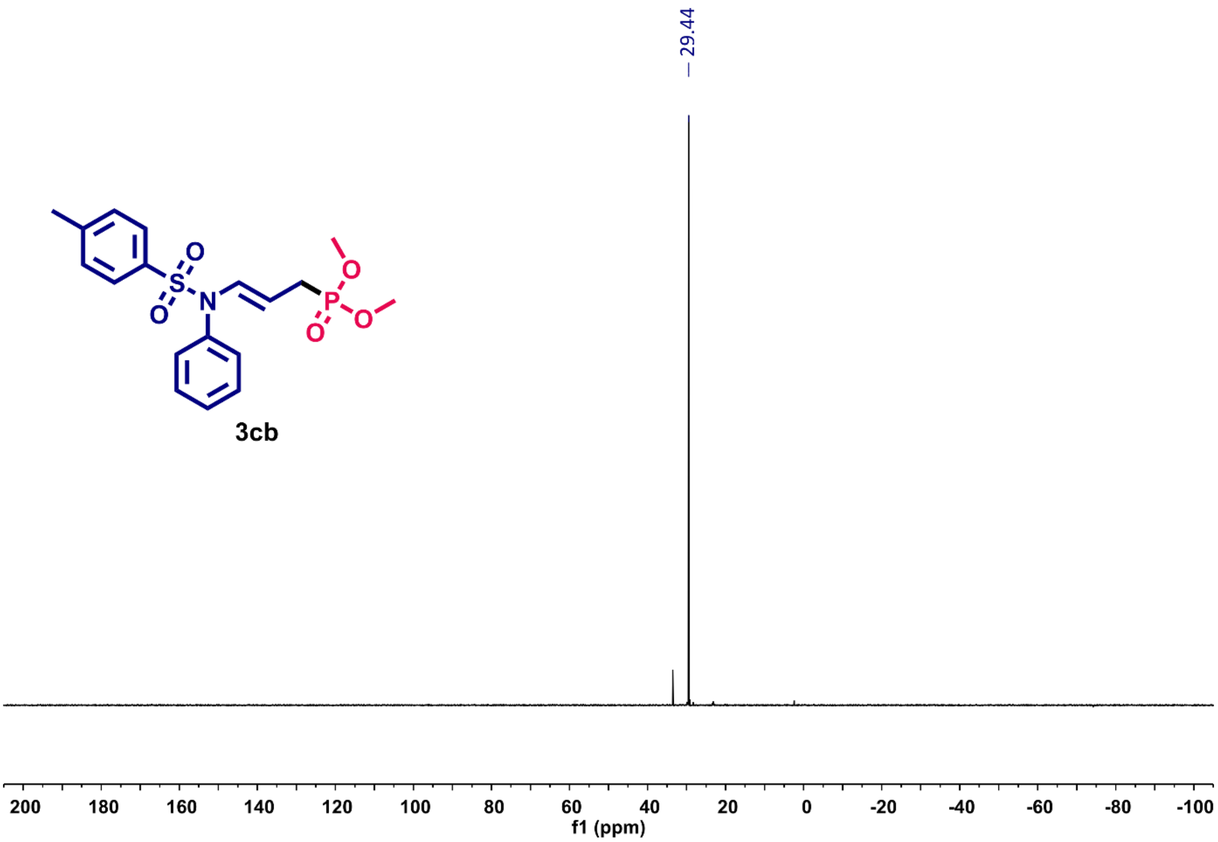

Figure S38. ^{31}P {^{1}H} NMR spectrum (162 MHz, $CDCl_3$) of *dimethyl (E)-(3-((4-methyl-N-phenylphenyl)sulfonamido)allyl) phosphonate* **3cb**.

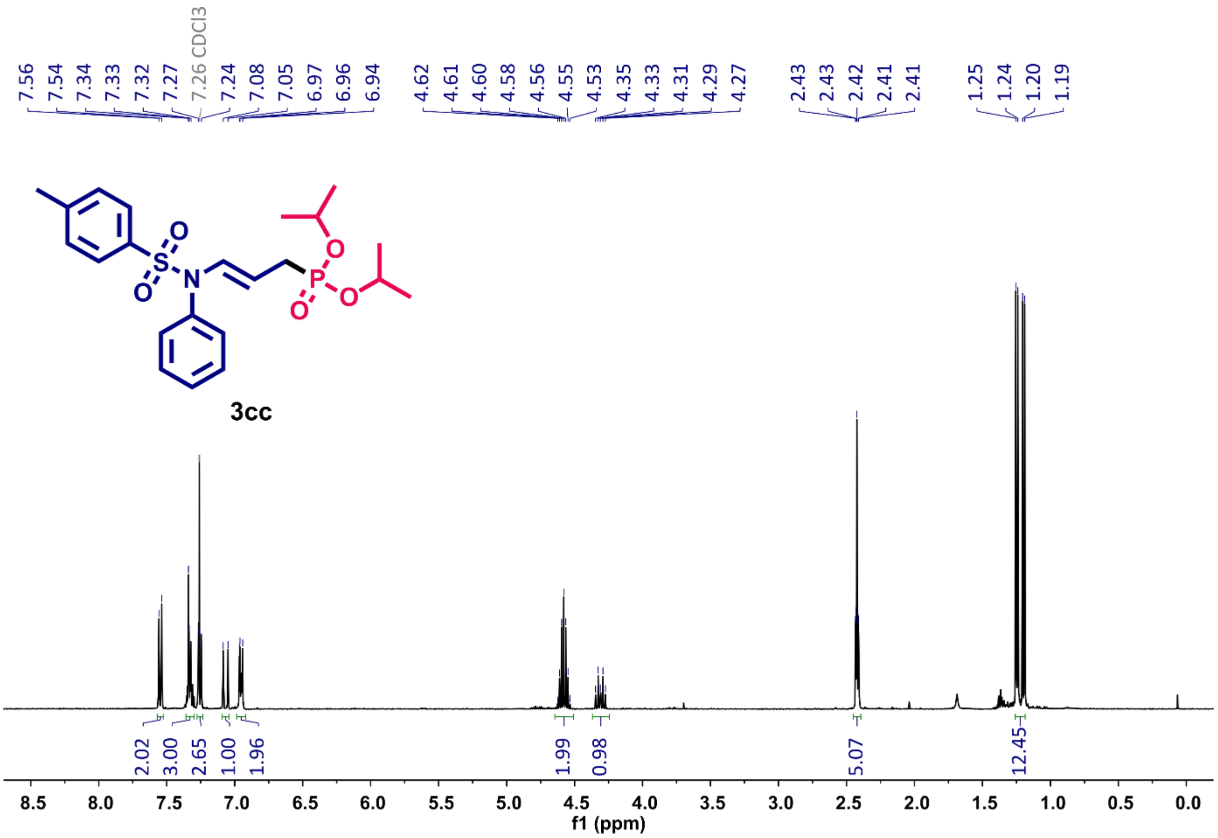

Figure S39. ^{1}H {^{31}P} NMR spectrum (400 MHz, $CDCl_3$) of *diisopropyl (E)-(3-((4-methyl-N-phenylphenyl)sulfonamido)allyl) phosphonate* **3cc**.

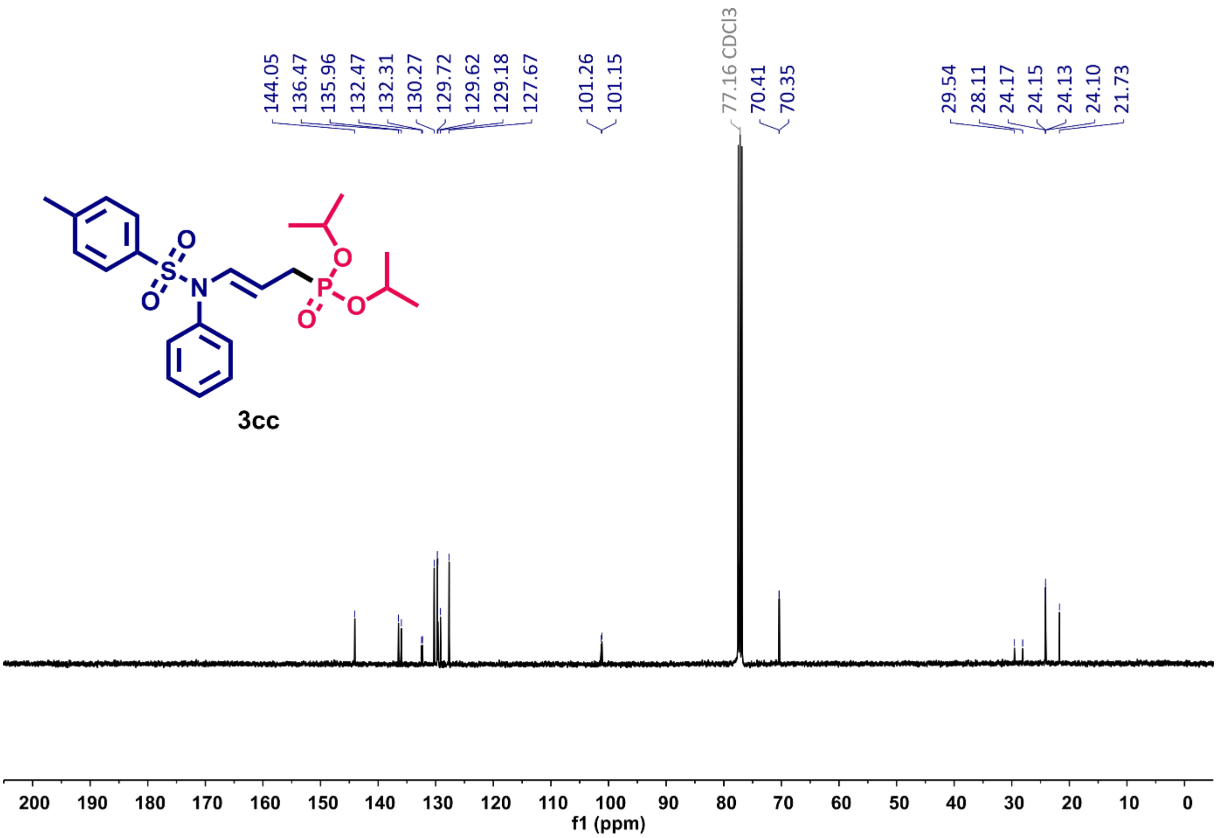

Figure S40. ^{13}C {^{1}H} NMR spectrum (101 MHz, $CDCl_3$) of *diisopropyl (E)-(3-((4-methyl-N-phenylphenyl)sulfonamido)allyl) phosphonate* **3cc**.

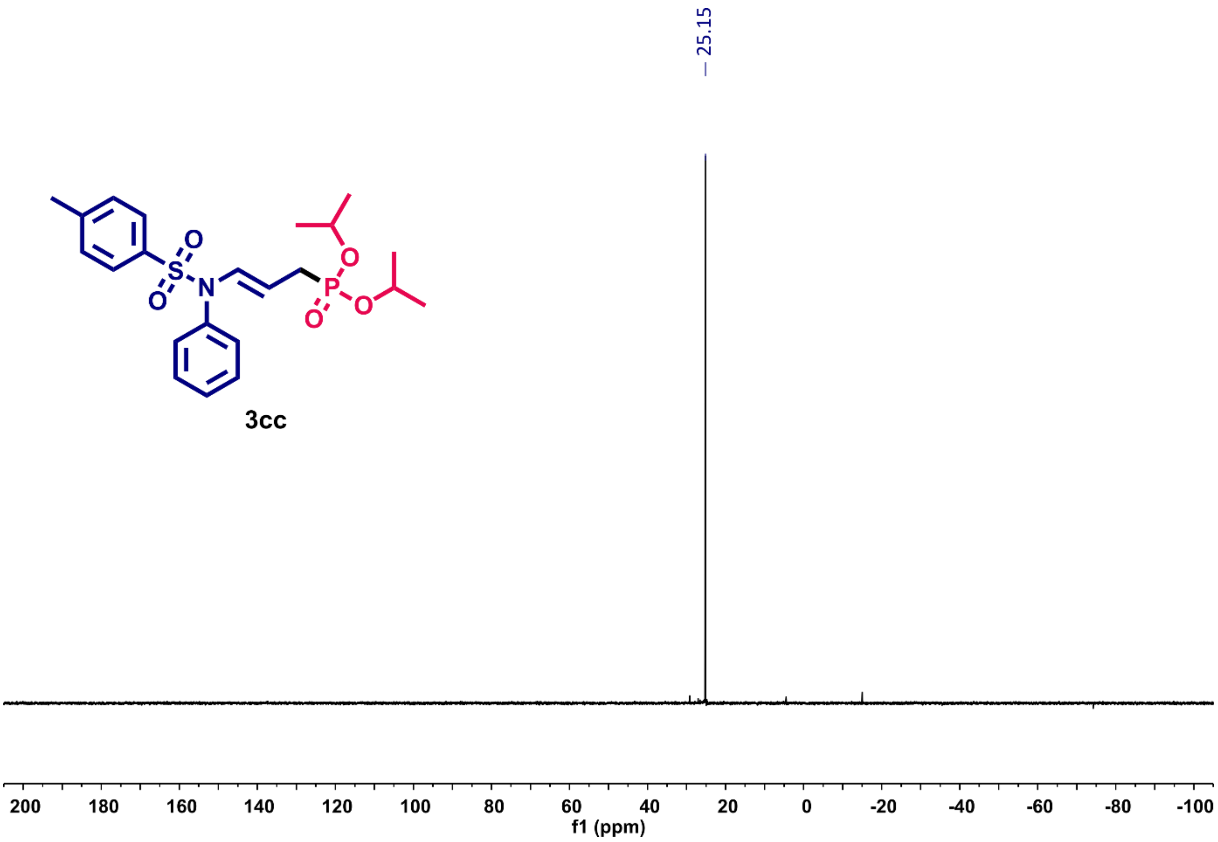

Figure S41. ^{31}P {^{1}H} NMR spectrum (162 MHz, $CDCl_3$) of *diisopropyl (E)-(3-((4-methyl-N-phenylphenyl)sulfonamido)allyl) phosphonate* **3cc**.

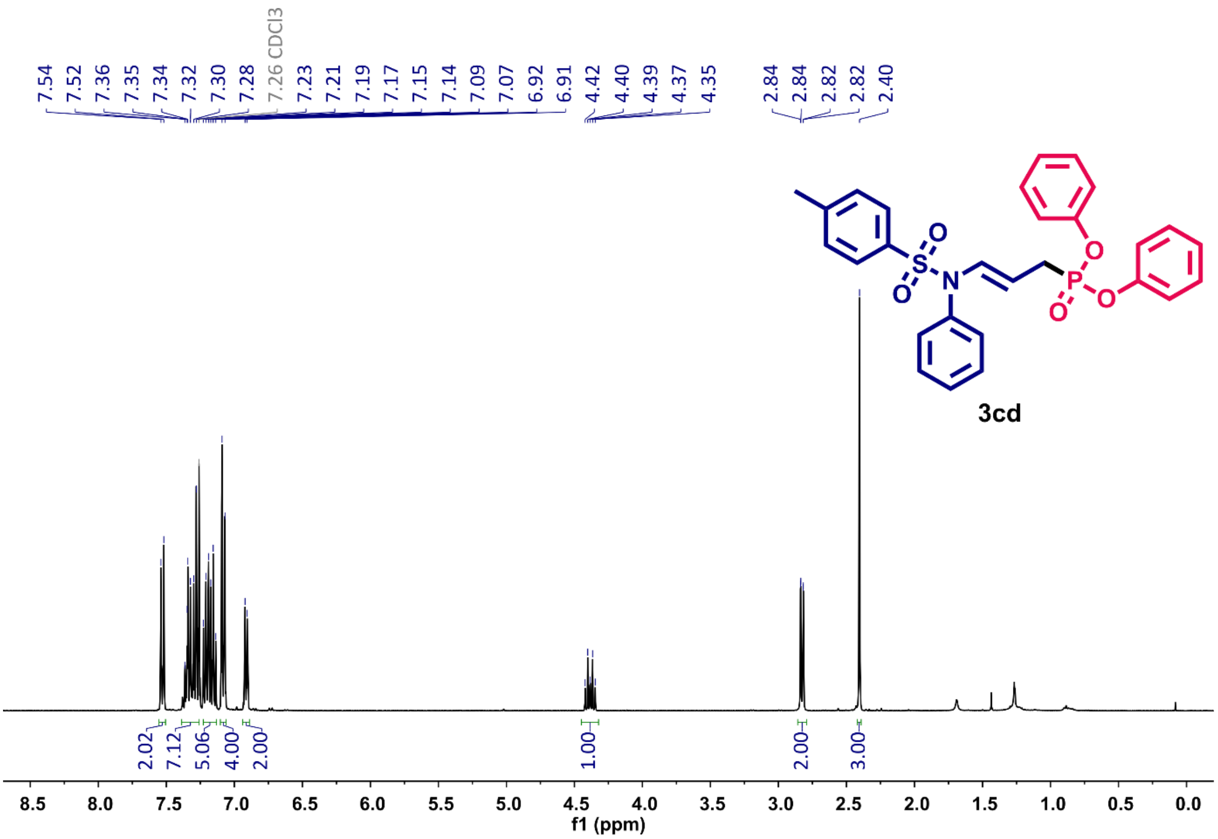

Figure S42. ^{1}H {^{31}P} NMR spectrum (400 MHz, $CDCl_3$) of *diphenyl (E)-(3-((4-methyl-N-phenylphenyl)sulfonamido)allyl) phosphonate* **3cd**.

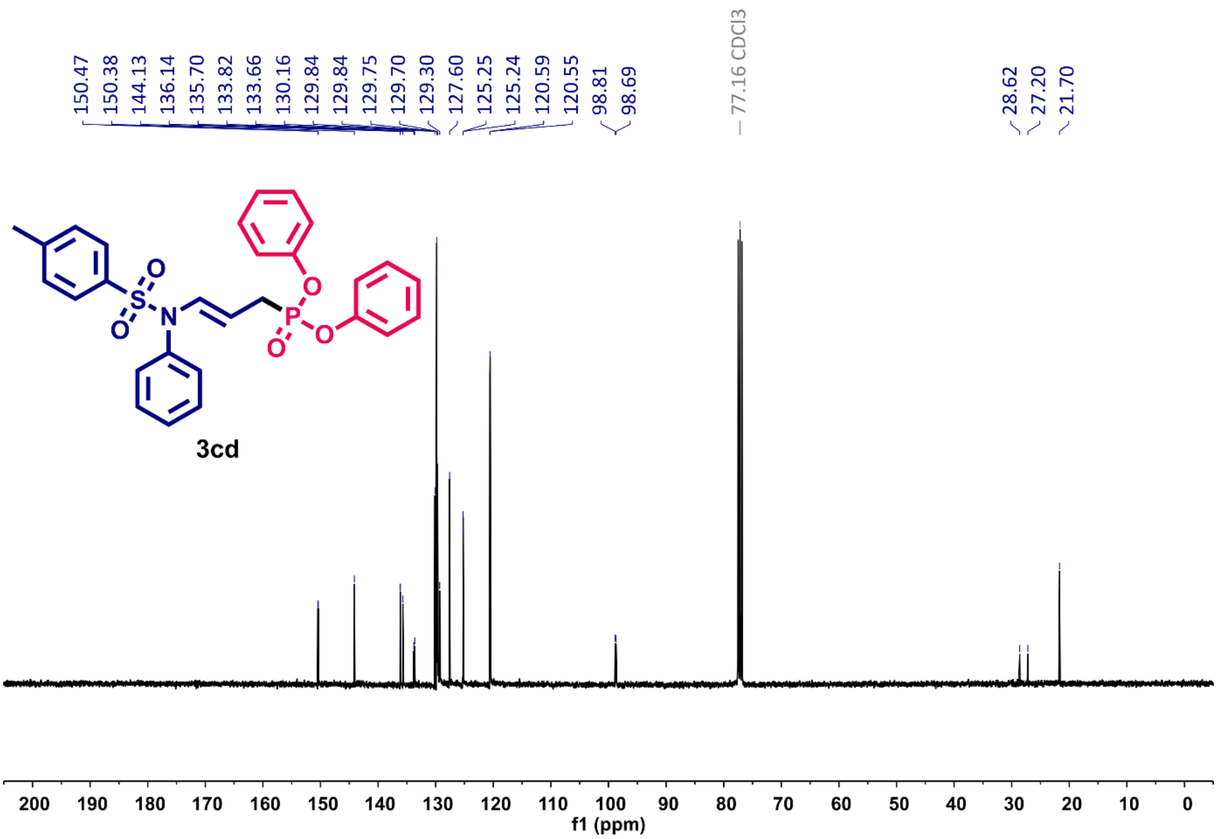

Figure S43. ^{13}C {^{1}H} NMR spectrum (101 MHz, $CDCl_3$) of *diphenyl (E)-(3-((4-methyl-N-phenylphenyl)sulfonamido)allyl) phosphonate* **3cd**.

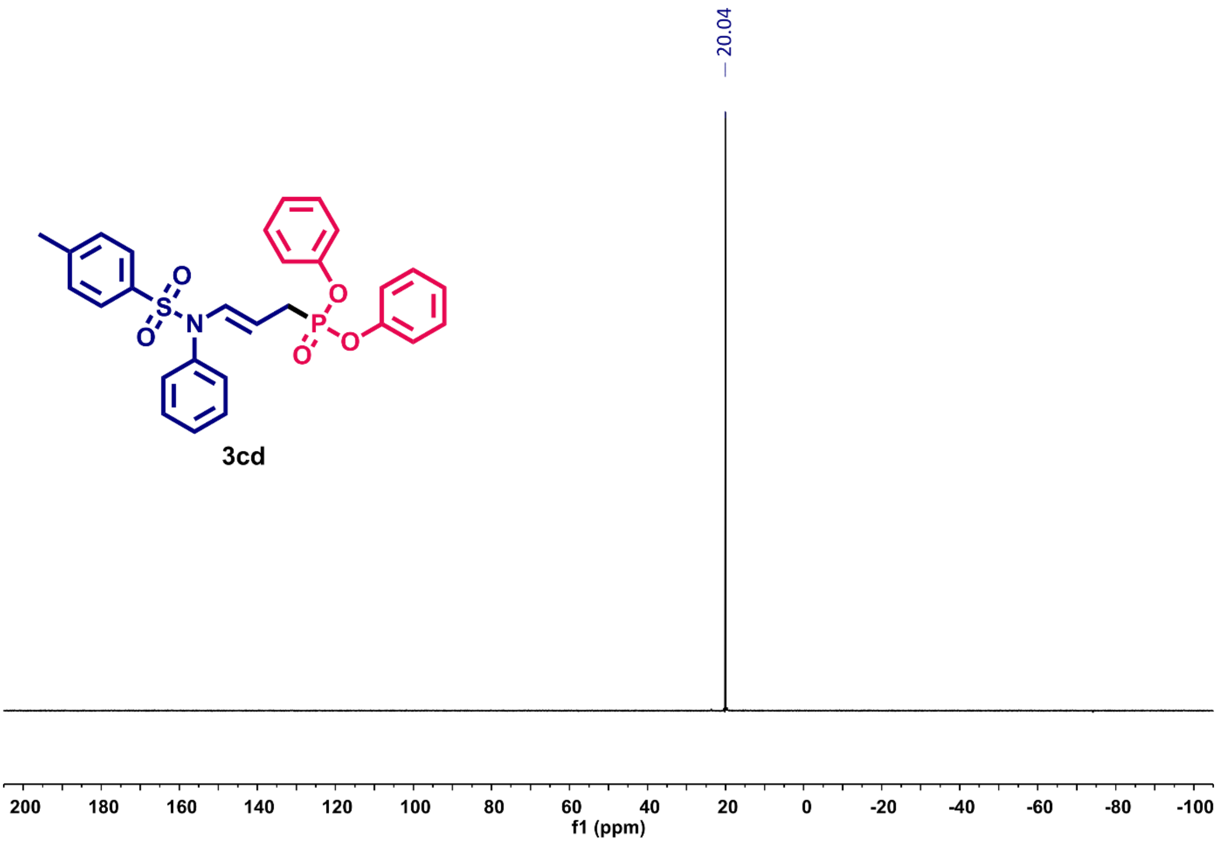

Figure S44. $^{31}P\{^{1}H\}$ NMR spectrum (162 MHz, $CDCl_3$) of *diphenyl (E)-(3-((4-methyl-N-phenylphenyl)sulfonamido)allyl) phosphonate* **3cd**.

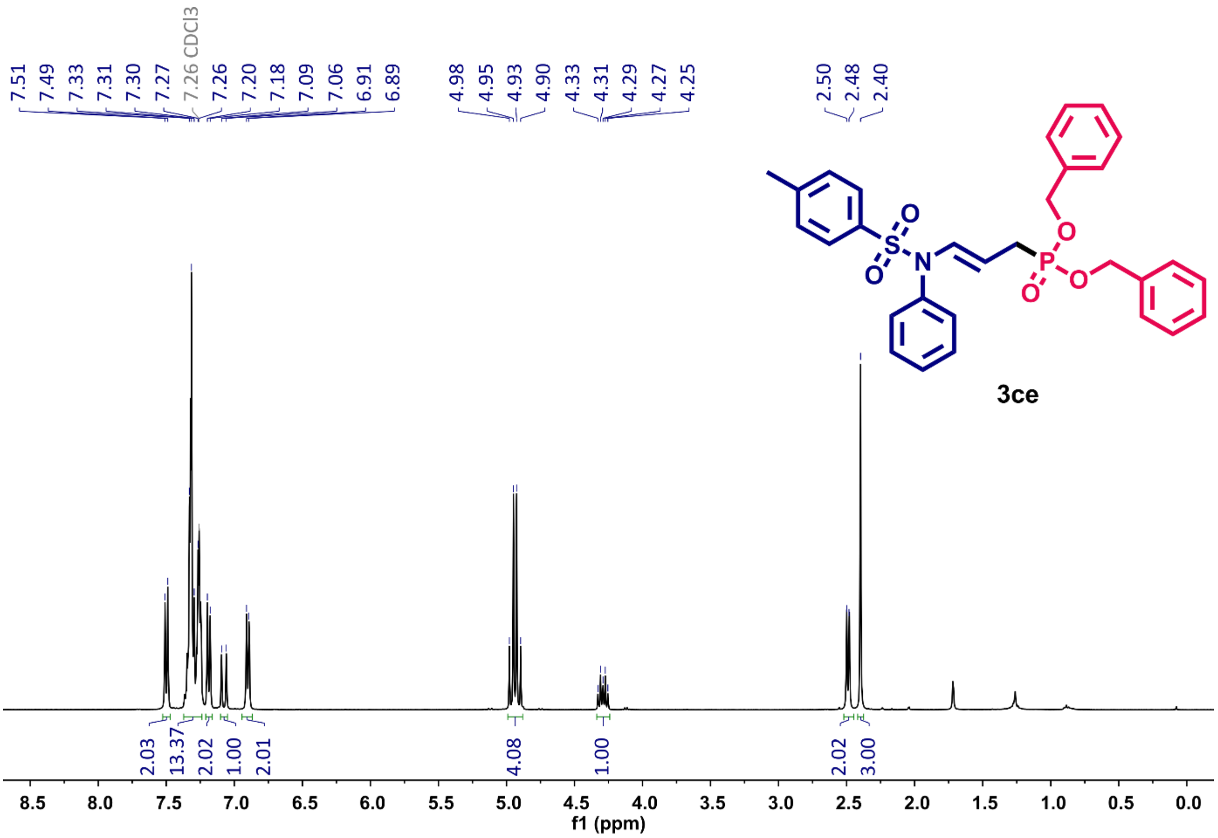

Figure S45. $^{1}H\{^{31}P\}$ NMR spectrum (400 MHz, $CDCl_3$) of *dibenzyl (E)-(3-((4-methyl-N-phenylphenyl)sulfonamido)allyl) phosphonate* **3ce**.

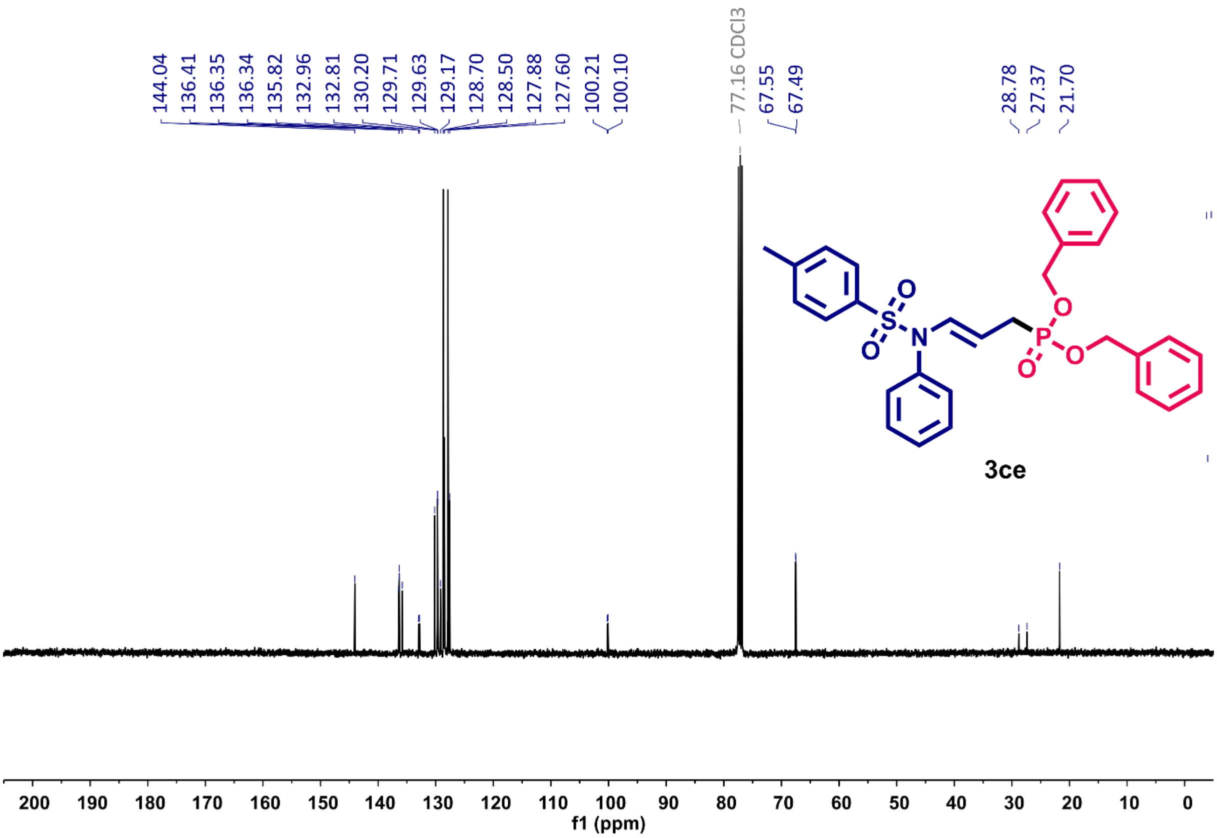

Figure S46. ^{13}C {^{1}H} NMR spectrum (101 MHz, $CDCl_3$) of *dibenzyl (E)-(3-((4-methyl-N-phenylphenyl)sulfonamido)allyl) phosphonate* **3ce**.

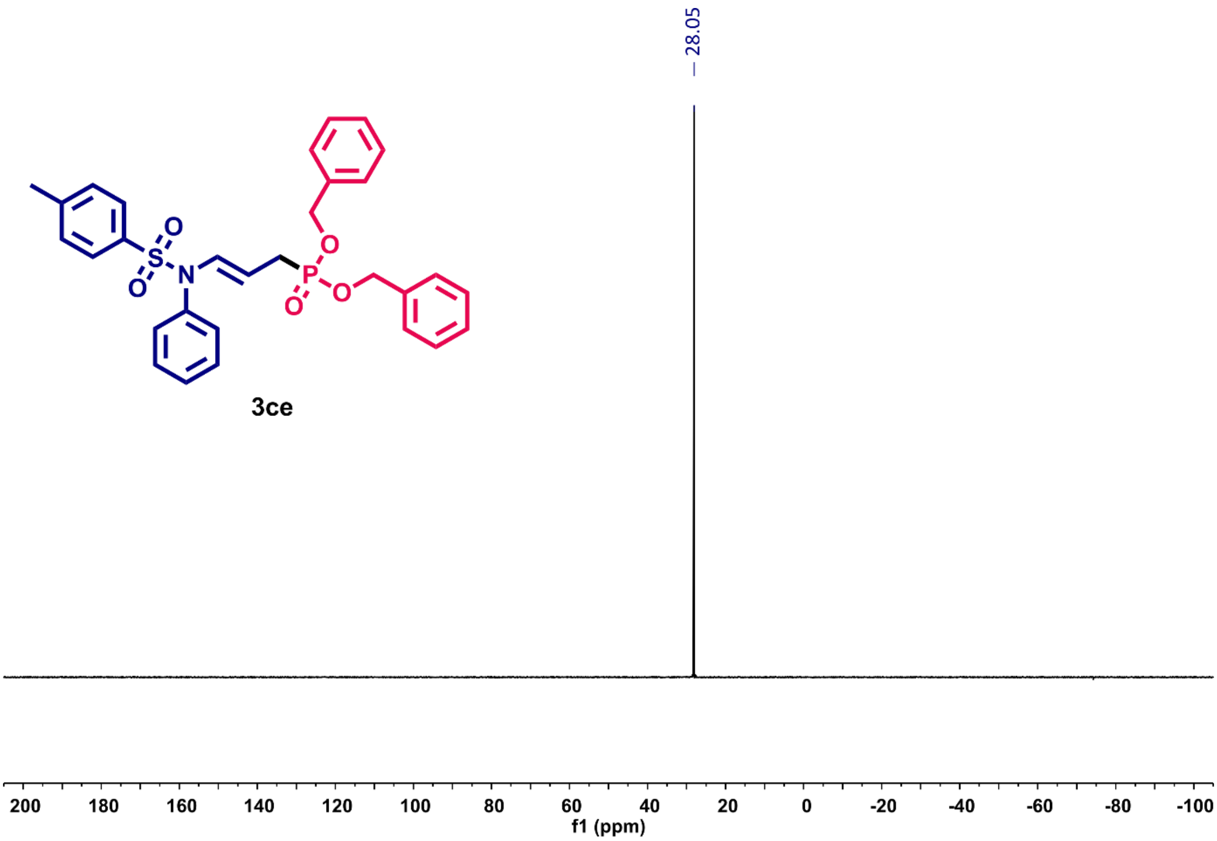

Figure S47. ^{31}P {^{1}H} NMR spectrum (162 MHz, $CDCl_3$) of *dibenzyl (E)-(3-((4-methyl-N-phenylphenyl)sulfonamido)allyl) phosphonate* **3ce**.

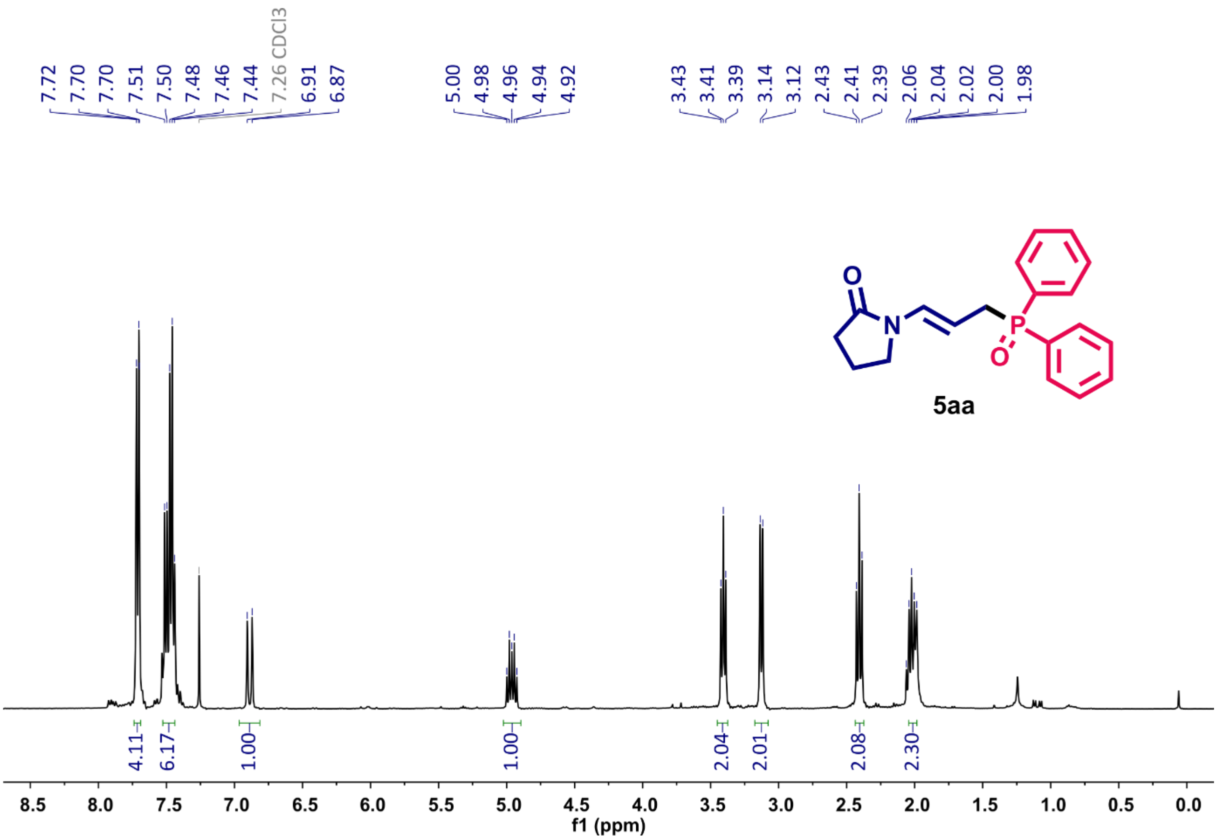

Figure S48. $^{1}H\{^{31}P\}$ NMR spectrum (400 MHz, $CDCl_3$) of *(E)-1-(3-(diphenylphosphoryl)prop-1-en-1-yl)pyrrolidin-2-one* **5aa**.

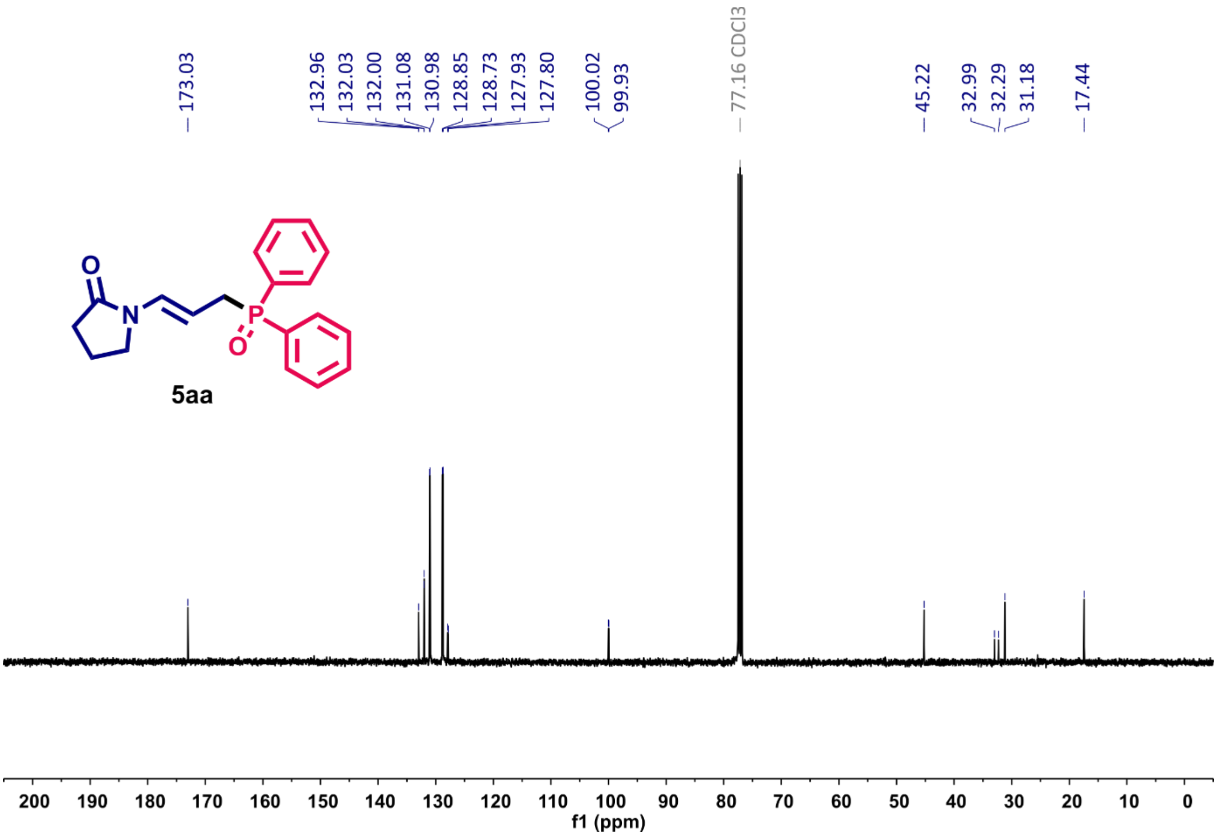

Figure S49. $^{13}C\{^{1}H\}$ NMR spectrum (101 MHz, $CDCl_3$) of *(E)-1-(3-(diphenylphosphoryl)prop-1-en-1-yl)pyrrolidin-2-one* **5aa**.

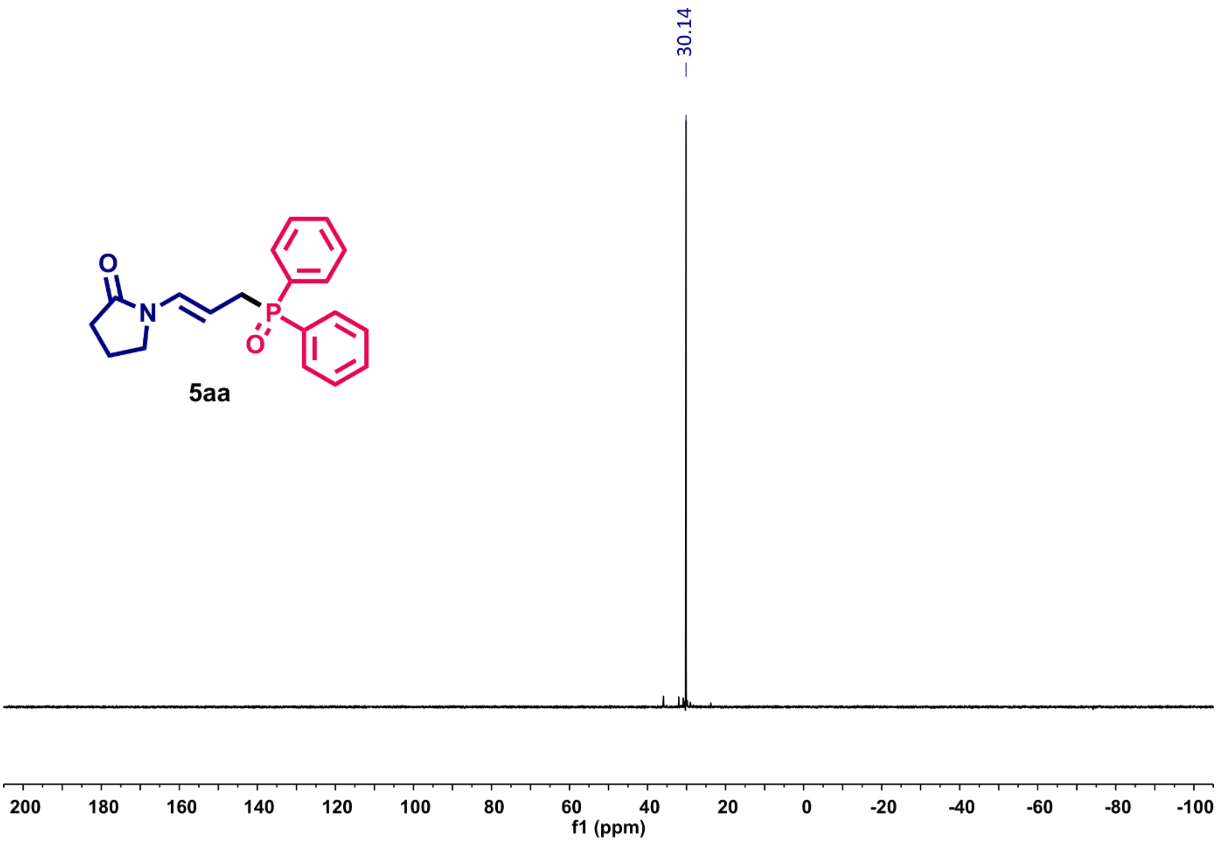

Figure S50. $^{31}P\ \{^{1}H\}$ NMR spectrum (162 MHz, $CDCl_3$) of *(E)-1-(3-(diphenylphosphoryl)prop-1-en-1-yl)pyrrolidin-2-one* **5aa**.

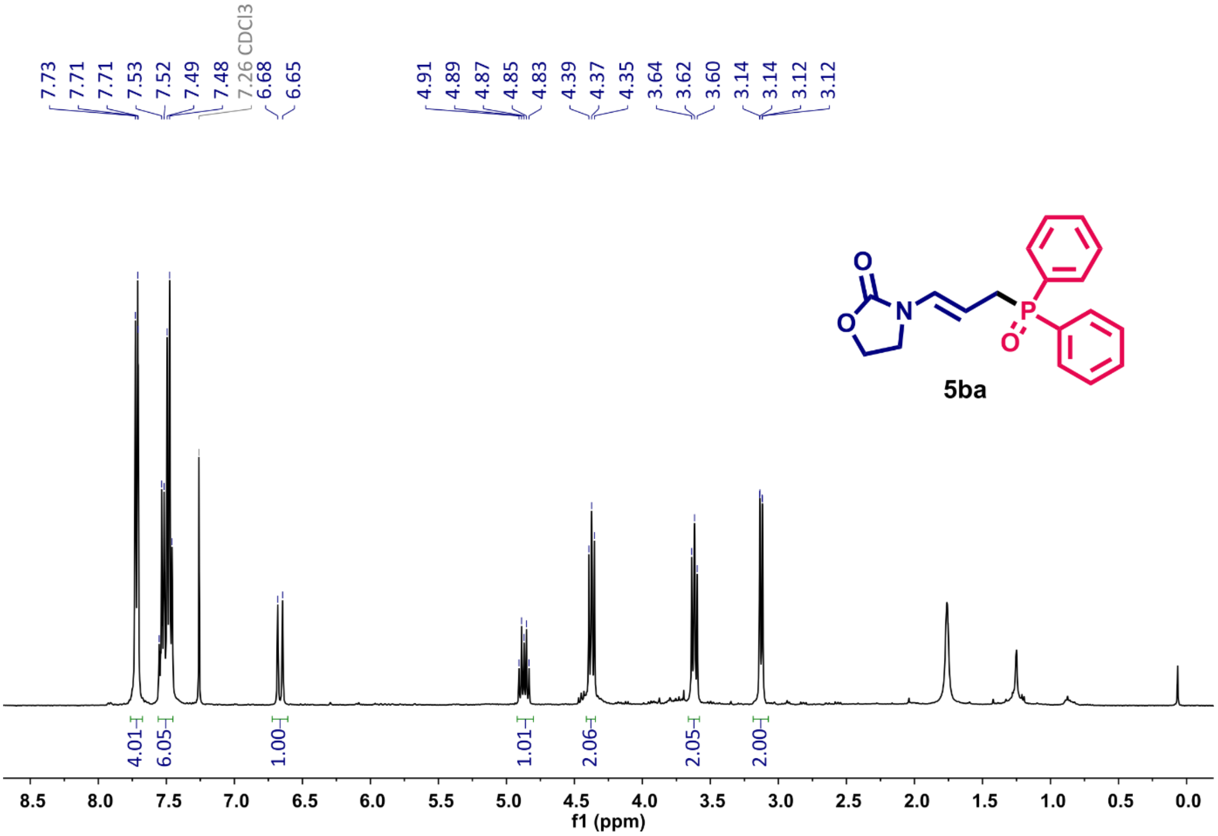

Figure S51. $^{1}H\ \{^{31}P\}$ NMR spectrum (400 MHz, $CDCl_3$) of *(E)-3-(3-(diphenylphosphoryl)prop-1-en-1-yl)oxazolidin-2-one* **5ba**.

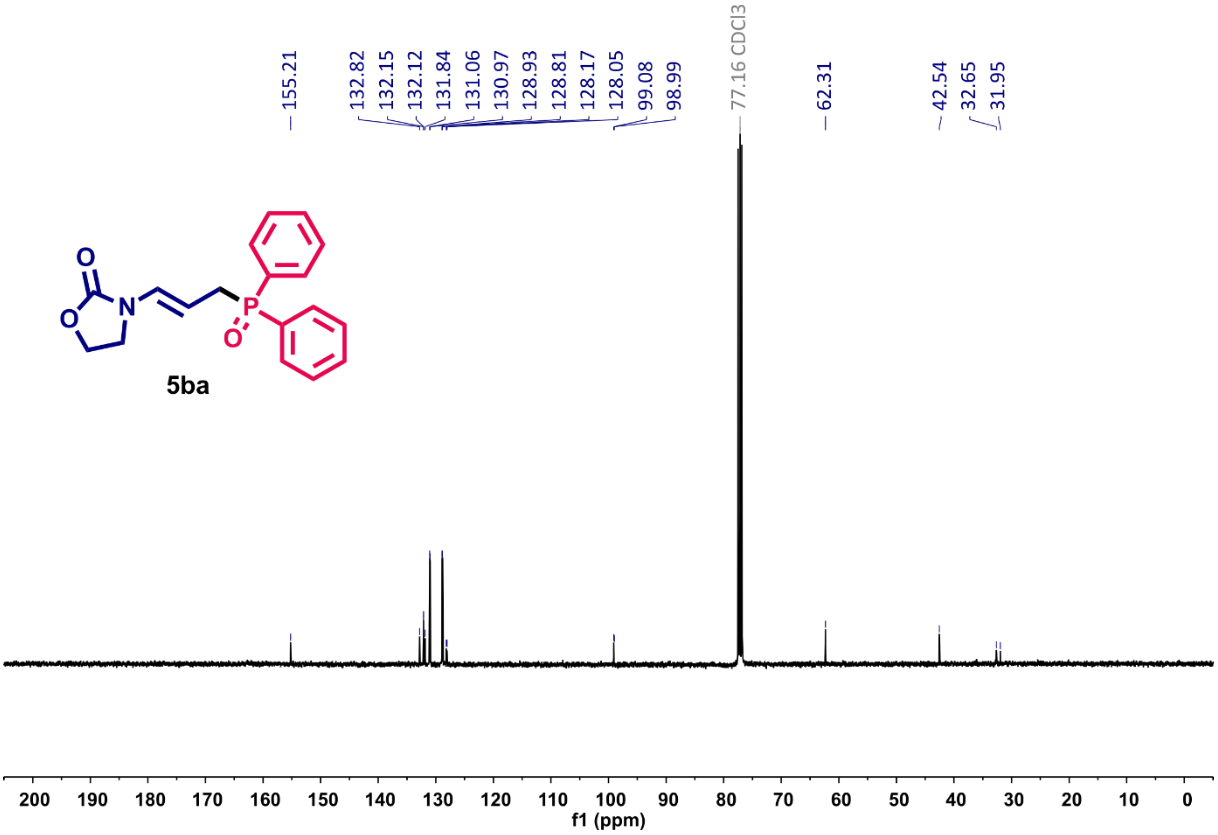

Figure S52. ^{13}C {^{1}H} NMR spectrum (101 MHz, $CDCl_3$) of *(E)-3-(3-(diphenylphosphoryl)prop-1-en-1-yl)oxazolidin-2-one* **5ba**.

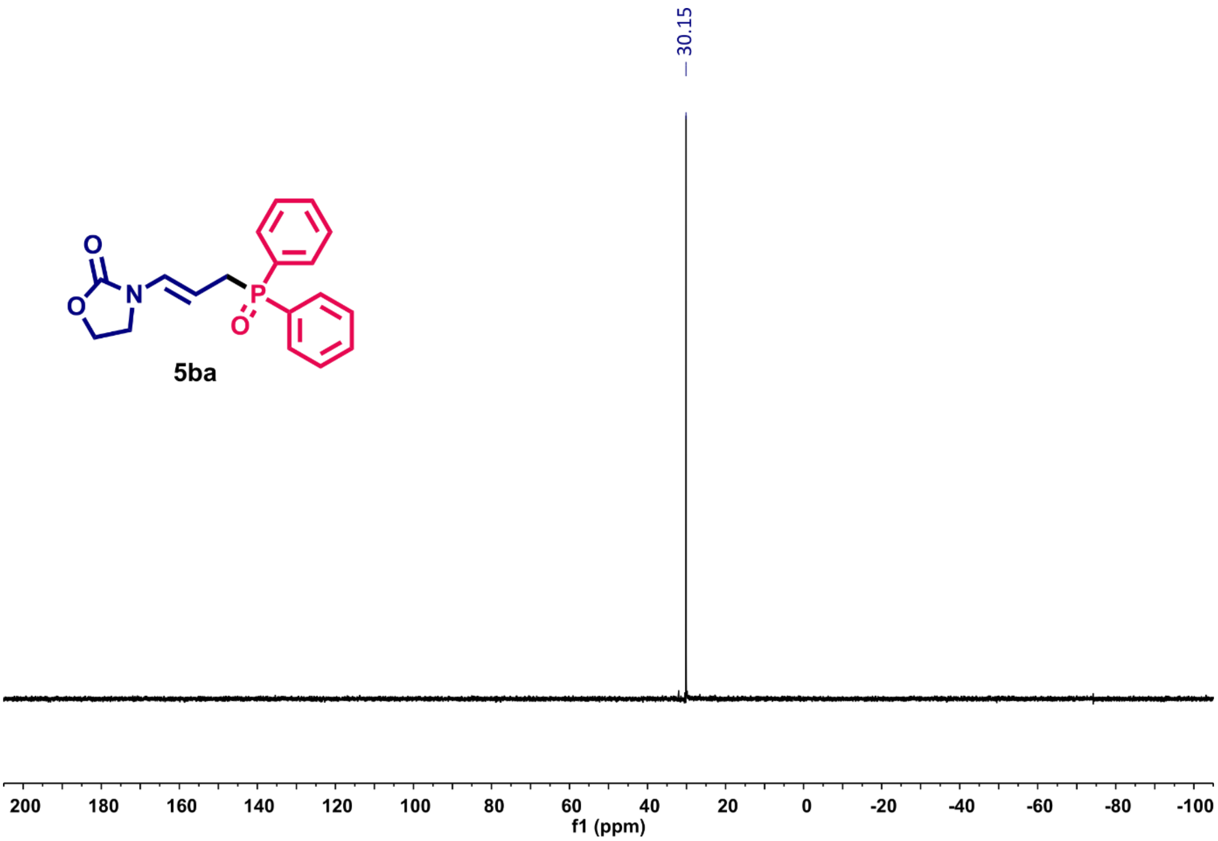

Figure S53. ^{31}P {^{1}H} NMR spectrum (162 MHz, $CDCl_3$) of *(E)-3-(3-(diphenylphosphoryl)prop-1-en-1-yl)oxazolidin-2-one* **5ba**.

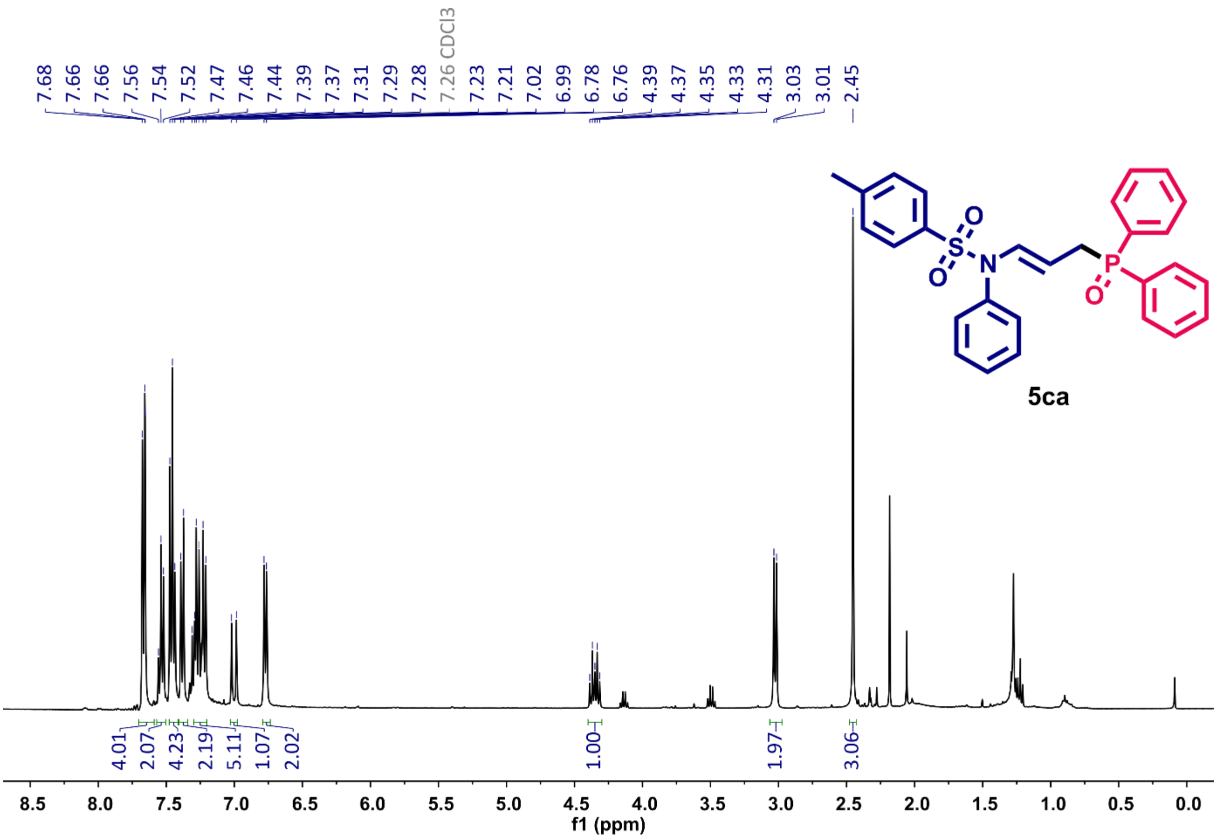

Figure S54. $^{1}H\ \{^{31}P\}$ NMR spectrum (400 MHz, $CDCl_3$) of *(E)-N-(3-(diphenylphosphoryl)prop-1-en-1-yl)-4-methyl-N-phenylbenzenesulfonamide* **5ca**.

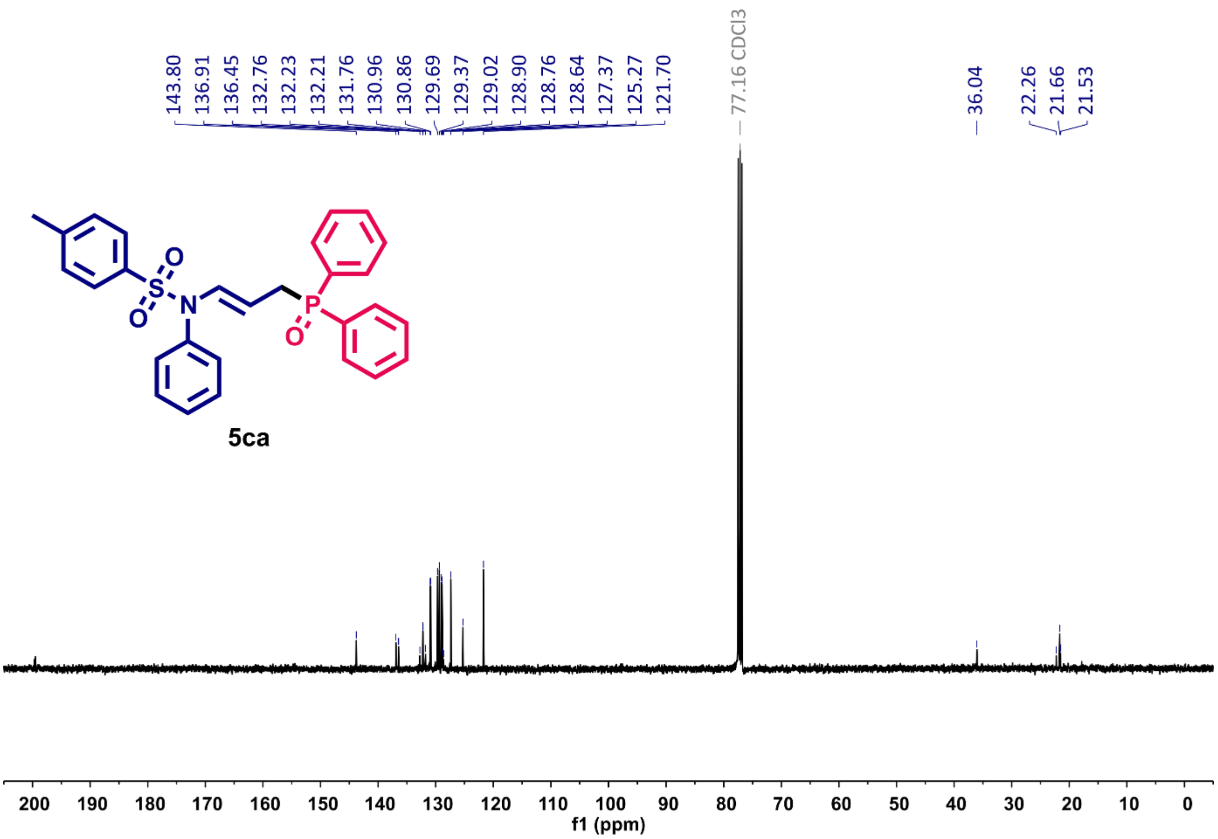

Figure S55. $^{13}C\ \{^{1}H\}$ NMR spectrum (101 MHz, $CDCl_3$) of *(E)-N-(3-(diphenylphosphoryl)prop-1-en-1-yl)-4-methyl-N-phenylbenzenesulfonamide* **5ca**.

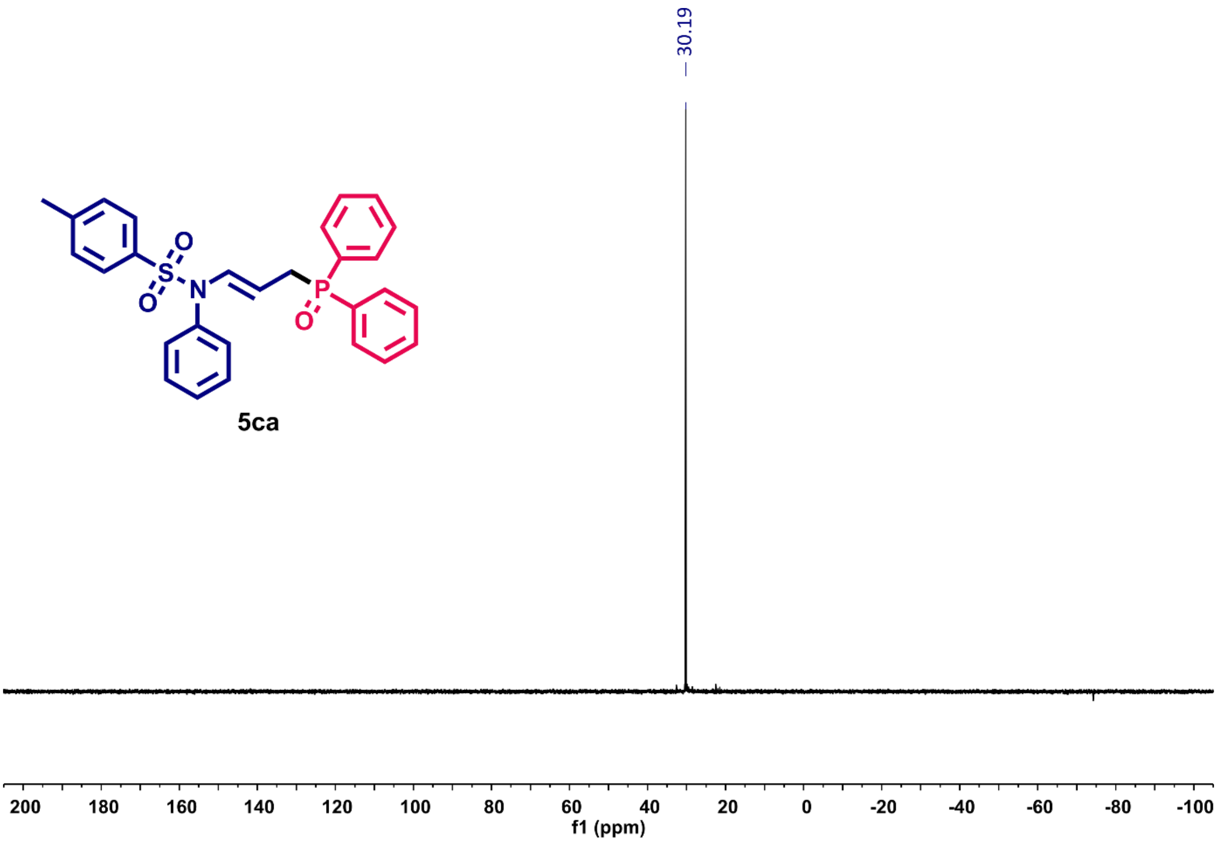

Figure S56. ^{31}P {^{1}H} NMR spectrum (162 MHz, $CDCl_3$) of *(E)-N-(3-(diphenylphosphoryl)prop-1-en-1-yl)-4-methyl-N-phenylbenzenesulfonamide* **5ca**.

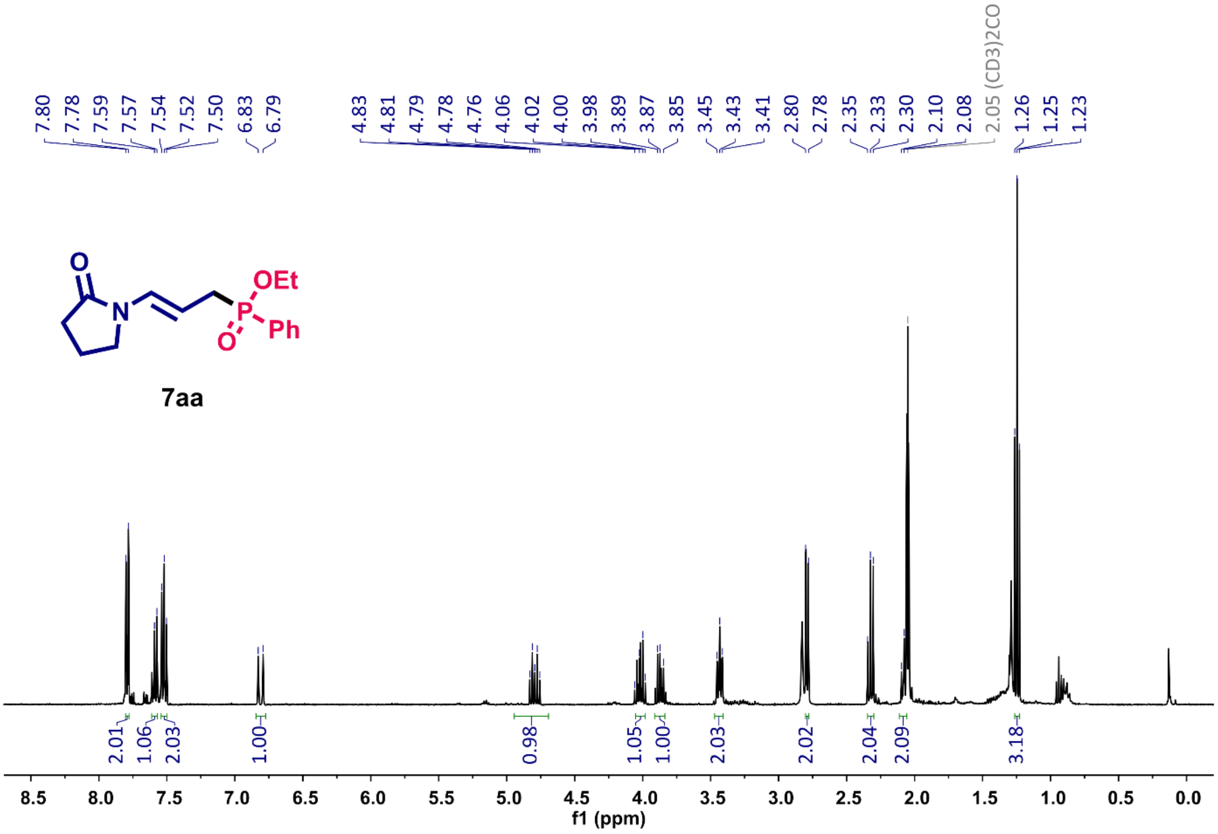

Figure S57. ^{1}H {^{31}P} NMR spectrum (400 MHz, acetone-d_6) of *ethyl (E)-(3-(2-oxopyrrolidin-1-yl)allyl)(phenyl)phosphinate* **7aa**.

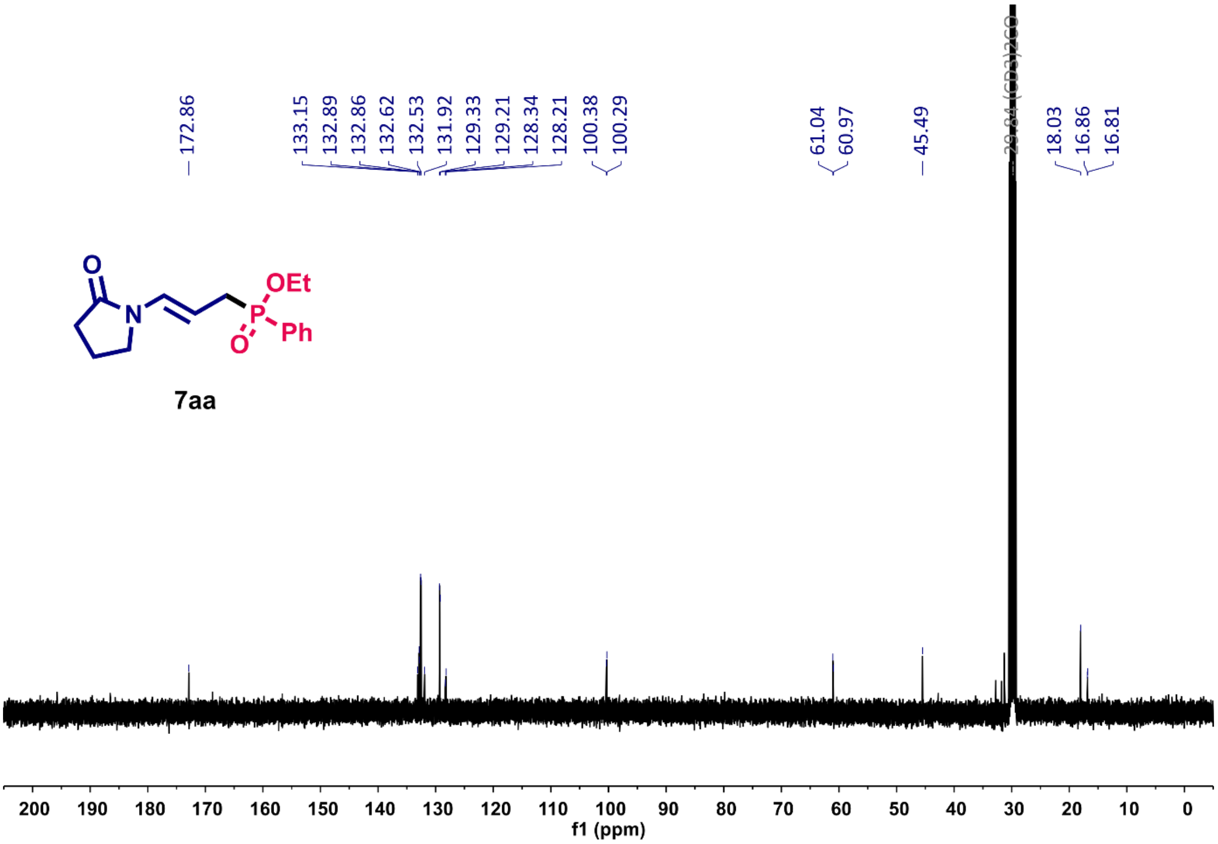

Figure S58. $^{13}C\{^{1}H\}$ NMR spectrum (101 MHz, $CDCl_3$) of *ethyl (E)-(3-(2-oxopyrrolidin-1-yl)allyl)(phenyl)phosphinate* **7aa**.

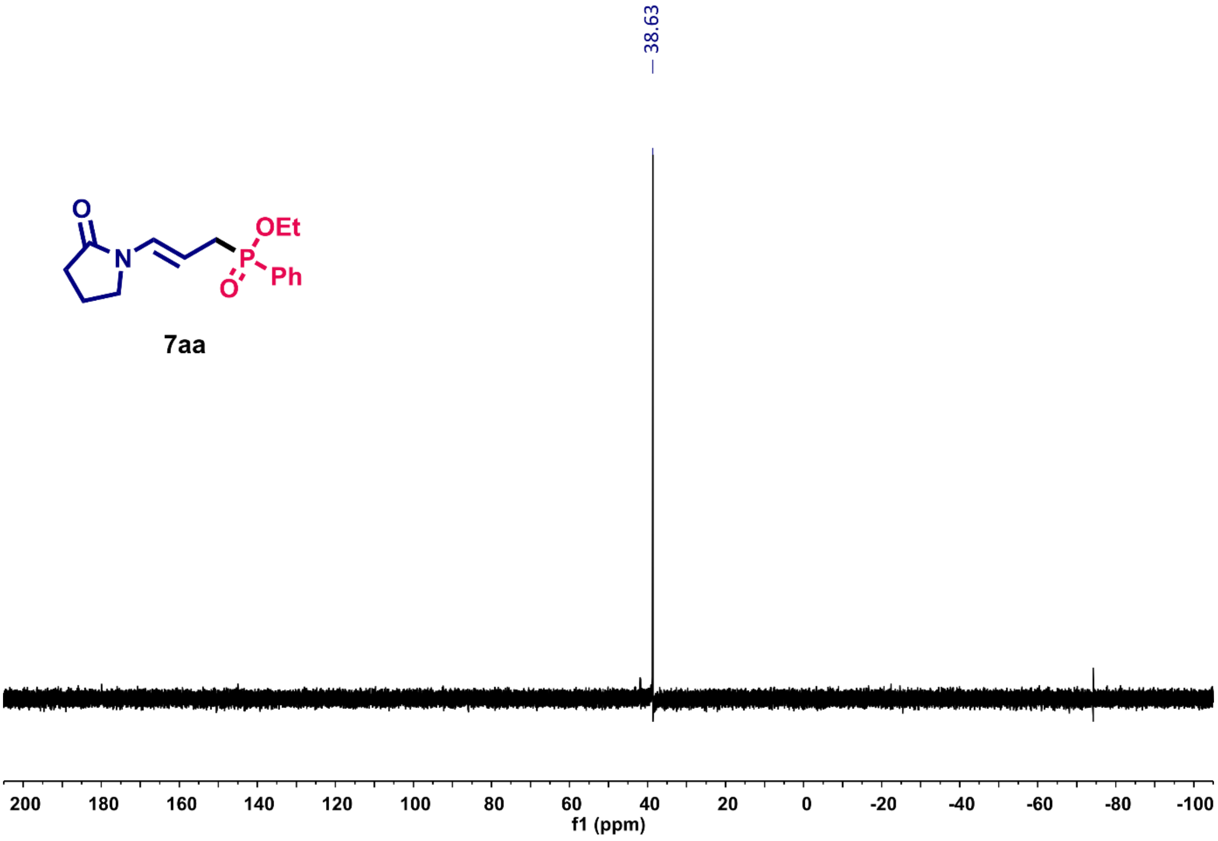

Figure S59. $^{31}P\{^{1}H\}$ NMR spectrum (162 MHz, acetone-d_6) of *ethyl (E)-(3-(2-oxopyrrolidin-1-yl)allyl)(phenyl)phosphinate* **7aa**.

10. References

1. E.J. Baerends, T. Ziegler, A.J. Atkins, J. Autschbach, O. Baseggio, D. Bashford, A. Bérces, F.M. Bickelhaupt, C. Bo, P.M. Boerrigter, L. Cavallo, C. Daul, D.P. Chong, D.V. Chulhai, L. Deng, R.M. Dickson, J.M. Dieterich, D.E. Ellis, M. van Faassen, L. Fan, T.H. Fischer, A. Förster, C. Fonseca Guerra, M. Franchini, A. Ghysels, A. Giammona, S.J.A. van Gisbergen, A. Goez, A.W. Götz, J.A. Groeneveld, O.V. Gritsenko, M. Grüning, S. Gusarov, F.E. Harris, P. van den Hoek, Z. Hu, C.R. Jacob, H. Jacobsen, L. Jensen, L. Joubert, J.W. Kaminski, G. van Kessel, C. König, F. Kootstra, A. Kovalenko, M.V. Krykunov, E. van Lenthe, D.A. McCormack, A. Michalak, M. Mitoraj, S.M. Morton, J. Neugebauer, V.P. Nicu, L. Noodleman, V.P. Osinga, S. Patchkovskii, M. Pavanello, C.A. Peeples, P.H.T. Philipsen, D. Post, C.C. Pye, H. Ramanantoanina, P. Ramos, W. Ravenek, J.I. Rodríguez, P. Ros, R. Rüger, P.R.T. Schipper, D. Schlüns, H. van Schoot, G. Schreckenbach, J.S. Seldenthuis, M. Seth, J.G. Snijders, M. Solà, M. Stener, M. Swart, D. Swerhone, V. Tognetti, G. te Velde, P. Vernooijs, L. Versluis, L. Visscher, O. Visser, F. Wang, T.A. Wesolowski, E.M. van Wezenbeek, G. Wiesenekker, S.K. Wolff, T.K. Woo, A.L. Yakovlev, ADF 2019.3, SCM, Theoretical Chemistry, Vrije Universiteit, Amsterdam, The Netherlands (2019)
2. G. te Velde, F. M. Bickelhaupt, E. J. Baerends, C. Fonseca Guerra, S. J. A. van Gisbergen, J. G. Snijders and T. Ziegler, Chemistry with ADF. J. Comput. Chem., 22, 931–967 (2001)
3. M. Swart and F. M. Bickelhaupt, QUantum-regions Interconnected by Local Descriptions. J. Comput. Chem., 29, 724–734 (2008)
4. E. van Lenthe and E. J. Baerends, Optimized Slater-type Basis Sets for the Elements 1–118. J. Comput. Chem., 24, 1142-1156 (2003)
5. D. P. Chong, E. van Lenthe, S. J. A. van Gisbergen and E. J. Baerends, Even-tempered Slater-type Orbitals Revisited: From Hydrogen to Krypton. J. Comput. Chem., 25, 1030–1036 (2004)
6. S. K. Wolff, Analytical Second Derivatives in the Amsterdam Density Functional Package. Int. J. Quantum Chem., 104, 645–659 (2005)
7. M. Swart, A new family of hybrid density functionals, Chem. Phys. Lett. 580, 166-171 (2013)
8. A. Klamt and G. Schüürmann, COSMO: A New Approach to Dielectric Screening in Solvents with Explicit Expressions for the Screening Energy and its Gradient. J. Chem. Soc. Perkin Trans. 2, 5, 799–805 (1993)
9. M. Swart, E. Rösler and F. M. Bickelhaupt, Proton Affinities in Water of Maingroup-Element Hydrides–Effects of Hydration and Methyl Substitution. Eur. J. Inorg. Chem., 23, 3646–3654 (2007)
10. B. B. Averkiev and D. G. Truhlar, Free Energy of Reaction by Density Functional Theory: Oxidative Addition of Ammonia by an Iridium Complex with PCP Pincer Ligands. Catal. Sci. Technol., 1, 1526–1529 (2011)
11. J. E. M. N. Klein, B. Dereli, L. Que Jr. and C. J. Cramer, Why Metal–oxos React with Dihydroanthracene and Cyclohexadiene at Comparable Rates, Despite Having Different C–H Bond Strengths. A Computational Study. Chem. Commun., 52, 10509–10512 (2016)
12. E. van Lenthe, E. J. Baerends and J. G. Snijders, Relativistic Regular Two-component Hamiltonians. J. Chem. Phys., 99, 4597–4610 (1993)
13. A. D. Becke, A multicenter numerical integration scheme for polyatomic molecules, J. Chem. Phys. 88, 2547-2553 (1988)
14. M. Franchini, P. H. T. Philipsen, L. Visscher, J. Comput. Chem. 34, 1819-1827 (2013)
15. M. D. Wilkinson, M. Dumontier, I. J. Aalbersberg, G. Appleton, M. Axton, A. Baak, N. Blomberg, J.-W. Boiten, L. B. da Silva Santos, P. E. Bourne, J. Bouwman, A. J. Brookes, T. Clark, M. Crosas, I. Dillo, O. Dumon, S. Edmunds, C. T. Evelo, R. Finkers, A. Gonzalez-Beltran, A. J. G. Gray, P. Groth, C. Goble, J. S. Grethe, J. Heringa, P. A. C. 't Hoen, R. Hooft, T. Kuhn, R. Kok, J. Kok, S. J. Lusher, M. E. Martone, A. Mons, A. L. Packer, B. Persson, P. Rocca-Serra, M. Roos, R. van Schaik, S.-A. Sansone, E. Schultes, T. Sengstag, T. Slater, G. Strawn, M. A. Swertz, M. Thompson, J. van der Lei, E. van Mulligen, J. Velterop, A. Waagmeester, P. Wittenburg, K. Wolstencroft, J. Zhao and B. Mons, Scientific Data, 3, 160018 (2016)
